# Integrative Bioinformatics and Functional Analyses of GEO, ENCODE, and TCGA Reveal *FADD* as a Direct Target of the Tumor Suppressor BRCA1

**DOI:** 10.3390/ijms19051458

**Published:** 2018-05-14

**Authors:** Dinh-Duc Nguyen, Dong Gyu Lee, Sinae Kim, Keunsoo Kang, Je-keun Rhee, Suhwan Chang

**Affiliations:** 1 Department of Biomedical Sciences, University of Ulsan School of Medicine, Asan Medical Center, Seoul 05505, Korea; michaelnguyen1986@gmail.com (D.-D.N.); que002@hanmail.net (D.G.L.); skim368@gmail.com (S.K.); 2 Department of Physiology, University of Ulsan School of Medicine, Asan Medical Center, Seoul 05505, Korea; 3 Department of Microbiology, College of Natural Sciences, Dankook University, Cheonan 31116, Korea; kangk1204@gmail.com; 4 Cancer Research Institute, Catholic University of Korea, Seoul 06591, Korea; jkrhee@catholic.ac.kr

**Keywords:** BRCA1, ENCODE, ChIP-seq, GEO, FADD

## Abstract

BRCA1 is a multifunctional tumor suppressor involved in several essential cellular processes. Although many of these functions are driven by or related to its transcriptional/epigenetic regulator activity, there has been no genome-wide study to reveal the transcriptional/epigenetic targets of BRCA1. Therefore, we conducted a comprehensive analysis of genomics/transcriptomics data to identify novel BRCA1 target genes. We first analyzed ENCODE data with BRCA1 chromatin immunoprecipitation (ChIP)-sequencing results and identified a set of genes with a promoter occupied by BRCA1. We collected 3085 loci with a BRCA1 ChIP signal from four cell lines and calculated the distance between the loci and the nearest gene transcription start site (TSS). Overall, 66.5% of the BRCA1-bound loci fell into a 2-kb region around the TSS, suggesting a role in transcriptional regulation. We selected 45 candidate genes based on gene expression correlation data, obtained from two GEO (Gene Expression Omnibus) datasets and TCGA data of human breast cancer, compared to BRCA1 expression levels. Among them, we further tested three genes (*MEIS2*, *CKS1B* and *FADD*) and verified *FADD* as a novel direct target of BRCA1 by ChIP, RT-PCR, and a luciferase reporter assay. Collectively, our data demonstrate genome-wide transcriptional regulation by BRCA1 and suggest target genes as biomarker candidates for BRCA1-associated breast cancer.

## 1. Introduction

Breast cancer type 1 (BRCA1) was identified more than 20 years ago, and is normally expressed in the breast and other tissues with multiple functions. Mutations in BRCA1 protein were first identified in familial breast cancer, and *BRCA1* has since been established as a strong breast and ovarian cancer susceptibility gene, responsible for approximately half of all inherited breast cancer cases [1]. The *BRCA1* gene is located on chromosome 17q21, and its mutations are associated with a high risk of breast cancer in women with a lifetime risk of 50–85% [2]. Since the discovery of BRCA1, there have been numerous studies aimed at fully understanding its biological function. BRCA1 is known to be involved in multiple cellular processes with various roles as a tumor suppressor or cell cycle regulator, contributing to its different vital functions such as in DNA damage repair, cell cycle checkpoint control, and ubiquitination [3,4,5,6]. In addition, BRCA1 also acts on gene promoters as a transcriptional regulator through C-terminal interaction to other transcription factors [7,8,9,10,11,12,13]. Thus, investigating the target genes regulated by BRCA1 would be an interesting approach for understanding the diverse functions of BRCA1.

Recent genome-wide research into the genetic elements involved in the regulation of transcription has provided massive information about several functional elements, including enhancers, promoters, and insulators. Identification of their location in the genome and their interactions with transcriptional factors are essential for understanding the mechanism of location-specific gene regulation. In particular, chromatin immunoprecipitation with massively parallel DNA sequencing (ChIP-seq) data generated by the Encyclopedia of DNA Elements (ENCODE) consortium have provided substantial amounts of regulatory information [14]. Moreover, the Gene Expression Omnibus (GEO) has collected a huge dataset of gene expression and other functional genomics data [15].

Here, we report the results from the combined analysis of BRCA1-dependent expression profiles and BRCA1 ChIP-seq data, which were verified in breast cancer cell lines. As examples, we demonstrate that the mRNA expression levels of three genes (*FADD*, *MEIS2*, *CKS1B*) have significant correlations to BRCA1 expression, and the promoters of the three genes interact with BRCA1. Furthermore, we found a positive correlation between BRCA1 and the three target genes in cancer cell lines and verified *FADD* as a novel direct target of BRCA1 by luciferase reporter, ChIP, and functional studies. Taken together, these data provide a comprehensive picture of the genome-wide transcriptional regulation by BRCA1 and suggest some of the identified target genes as biomarker candidates for BRCA1-associated breast cancer.

## 2. Results

### 2.1. Identification of Potential BRCA1 Target Genes from ENCODE, GEO, and TCGA Data Analysis

**Figure 1:**
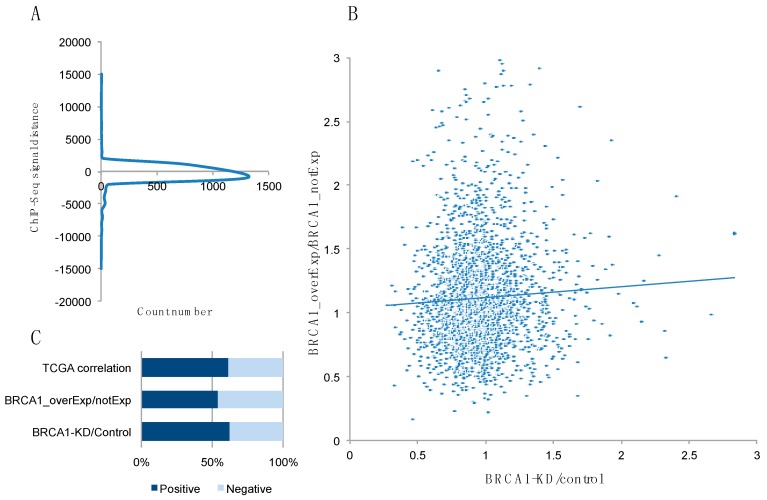
Integrated bioinformatics analysis to identify BRCA1 target genes. (**A**) Schematic visualization of the BRCA1 ChIP-seq signal from ENCODE data, showing the number of genes according to the distance between the site of the ChIP signal and transcription start site (TSS). Note that the peak of the graph is below the x-axis, indicating that more ChIP signals are derived from upstream of the TSS than downstream; (**B**) Dot plot showing the correlation between BRCA1 overexpression and knockdown; (**C**) Degree of expression correlation of BRCA1 with other genes in overexpression, knockdown of BRCA1, and TCGA datasets. Positive correlation means the expression level of a certain gene is increased by BRCA1 overexpression or decreased by BRCA1 knockdown.

To identify novel BRCA1 target genes, we analyzed ENCODE data by collecting the loci with a BRCA1 ChIP signal. We collected a total of 3086 loci found in all four cell lines in the database (hES, HeLa, HepG2, and GM12878), and calculated the distance from each locus to the nearest gene transcription start site (TSS). The raw data for the BRCA1 ChIP signal are provided in Supplementary Table S1. The results showed that most of the ChIP signals fell within 2 kb (in both directions) of its promoter (Figure 1A). Based on these data, the genes with a BRCA1-binding signal were compared with the BRCA1-dependent expression profile data from GEO datasets. The GSE30822 dataset contains expression profiles of HCC1937 cells, a BRCA1-deficient cell line, along with HCC1937 cells reconstituted with wild-type (WT) BRCA1. By contrast, the GSE22259 dataset provides expression profiles of MCF7 cells with BRCA1 knockdown. When we compared the two datasets, we found only a weak positive correlation (Figure 1B). Therefore, we added breast cancer expression profiles from The Cancer Genome Atlas (TCGA) database to select the genes showing highly correlated expression with BRCA1 (Supplementary Table S2). Figure 1C shows that there was a higher portion of genes positively correlated with BRCA1 when integrating the three datasets. We selected 45 genes that showed either consistent BRCA1-dependent expression change (in two GEO datasets) or high BRCA1 correlation based on the TCGA data (Supplementary Table S3). For further investigation, we selected three potential target genes, *FADD, MEIS2* and *CKS1B,* which have a potential functional relation to BRCA1 (Table 1). Among them, *MEIS2* was selected as a candidate long-range interacting target gene, as the BRCA1 ChIP signal was detected 521 kb upstream of the TSS of *MEIS2*, which was the closest annotated TSS. We confirmed a significant correlation between the expression levels of these genes and the BRCA1 level (Table 1).

### 2.2. BRCA1 Overexpression Positively Regulates FADD, MEIS2, and CKS1B Expression

**Figure 2:**
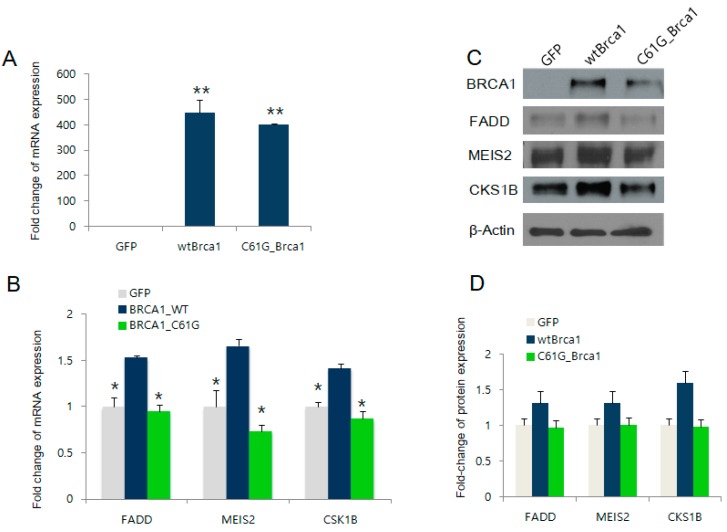
Identification of BRCA1 target genes. (**A**) Real-time PCR results of *BRCA1* in BRCA1- deficient MDA-MB-436 cells or cells transfected with WT BRCA1 or the C61G mutant BRCA1 construct; the GFP expression plasmid was used as a control; (**B**) mRNA levels of *FADD*, *CKS1B*, and *MEIS2* measured by real-time PCR after the expression of either WT or C61G BRCA1; (**C**) The same samples were analyzed by western blot to detect protein levels; (**D**) Densitometry measurement of the data in (**C**). * *p* < 0.05, ** *p* < 0.01.

Following the bioinformatics analysis, we examined the BRCA1-dependent expression of the four selected candidate target genes in BRCA1-deficient MDA-MB-436 cells, by the reconstitution of WT/C61G mutant BRCA1. The protein and mRNA levels of BRCA1 were obtained by western blotting and real-time polymerase chain reaction (PCR), respectively. The results in Figure 2A demonstrated comparable expression of WT BRCA1 or the C61G pathogenic mutant. We also observed significant upregulation of *FADD*, *CKS1B*, and *MEIS2* by WT BRCA1, consistent with the bioinformatics analysis as shown in Table 1 (Figure 2B). In contrast, the C61G pathogenic mutant BRCA1 failed to increase the expression of these three genes, indicating their dependency on functional BRCA1. These results were further confirmed by the western blot results (see Figure 2C,D for densitometry measurements).

### 2.3. BRCA1 Knockdown Negatively Regulates FADD, MEIS2, and CKS1B Expression

**Figure 3:**
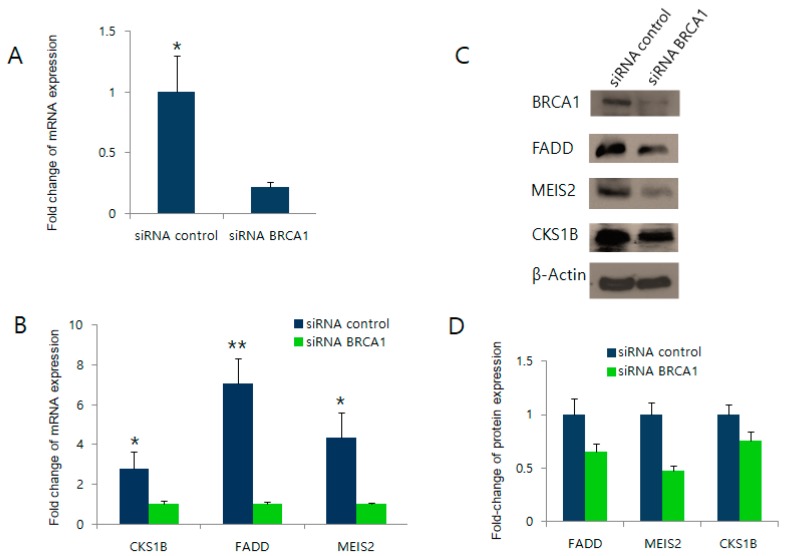
Knockdown of BRCA1 affects candidate target gene expression. (**A**) Real-time PCR results of *BRCA1* expression in HEK293 cells transfected with siRNA of BRCA1 or a scrambled control; (**B**) mRNA levels of *FADD*, *CKS1B*, and *MEIS2* measured by real-time PCR after the knockdown of BRCA1; (**C**) The same samples were analyzed by western blot to detect protein levels. β-Actin was used as a loading control; (**D**) Densitometry measurement of the western blotting data in (**C**). * *p* < 0.05, ** *p* < 0.01.

We next tested whether BRCA1 knockdown would also alter the expression of the three BRCA1 target candidate genes. Due to the poor efficiency of the small interfering RNA (siRNA) siRBA1 in MCF7 cells, we introduced the siRNA to HEK393T cells (Figure 3). As shown in Figure 3A, the siRNA-mediated knockdown of BRCA1 efficiently reduced BRCA1 expression. In the BRCA1 knockdown cells, the expression levels of the three candidate target genes were also significantly reduced at both the mRNA level (Figure 3B) and protein level (Figure 3C,D). Together, these data support that BRCA1 transcriptionally upregulates *FADD, MEIS2*, and *CKS1B* expression, as candidates selected from the bioinformatics analysis.

### 2.4. BRCA1 Activates the Promoter Region of FADD but Not the Other Target Genes

**Figure 4:**
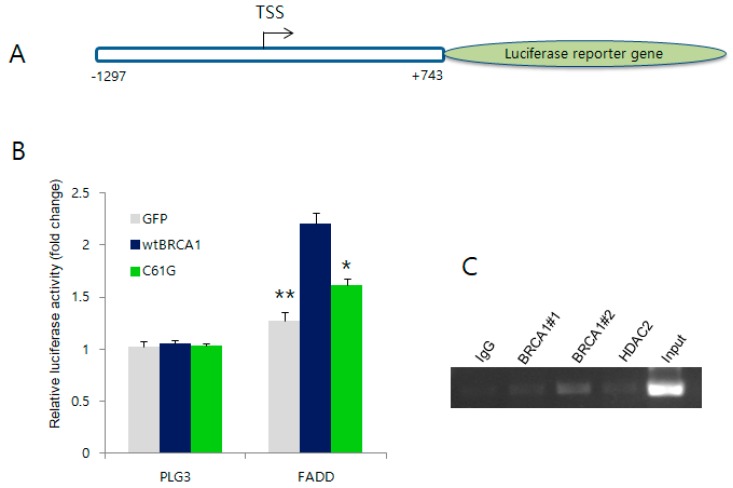
Activation of the *FADD* promoter by direct interaction of BRCA1. (**A**) Schematic presentation of the promoter construct of *FADD* used for the luciferase assay; (**B**) Results of the promoter assay. The *FADD* gene promoter was activated by WT BRCA1 (in deep blue) but was markedly reduced by the C61G mutant (in green). PGL3 vector only was used as a negative control; (**C**) ChIP assay indicating that BRCA1 physically associated with the *FADD* promoter. IgG was used as a negative control. Two different BRCA1 antibodies, #1 and #2, were used for ChIP [6]. * *p* < 0.05, ** *p* < 0.01.

Next, we explored the mechanism(s) by which BRCA1 transcriptionally upregulates the expression of the three candidate genes. As the ChIP signal from the ENCODE database suggested a putative BRCA1 interaction on the promoter region of each gene (except for *MEIS2*), we tested the effect of BRCA1 expression on activation of the candidate gene promoters. Each candidate promoter reporter construct was co-transfected with either the WT or C61G mutant BRCA1 expression vector into human breast carcinoma MDA-MB-436 cells, followed by a luciferase reporter assay. As a control, the cells were transfected with the PLG3 vector. Among the three candidates, *FADD* promoter reporter constructs (Figure 4A) showed significant activation following the expression of WT BRCA1, but not by the C61G mutant, in MDA-MB-436 cells (Figure 4B, and see Supplementary Figure S1 for the results of other candidates). To further clarify whether BRCA1 interacts with the *FADD* promoter, we performed ChIP analysis in MCF7 cells using two different BRCA1 antibodies and an antibody for HDAC2 that was previously shown to interact with BRCA1 in miR-155 regulation [6]. Figure 4C shows that only the #2 BRCA1 antibody detected a BRCA1 interaction on the *FADD* promoter. Taken together, these results indicate that induction of *FADD* mRNA occurs by BRCA1 through interaction on the *FADD* promoter with subsequent transcriptional activation.

### 2.5. Fas Ligand (FasL) Signaling and BRCA1 Expression Reduce Proliferation

**Figure 5:**
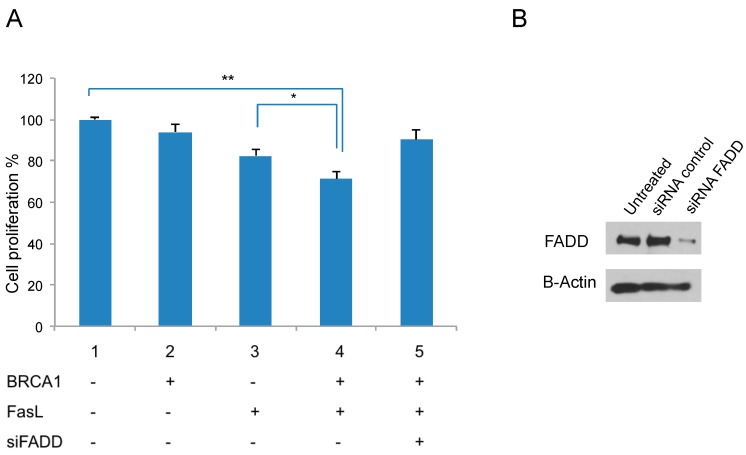
BRCA1 expression sensitizes cancer cells to FasL via FADD. (**A**) Proliferation assay of MDA-MB-436 cells treated with FasL in combination with BRCA1 and siRNA of *FADD*; (**B**) Western blot analysis of FADD to confirm efficient knockdown. β-Actin was used as a loading control. * *p* < 0.05, ** *p* < 0.01.

To understand how BRCA1 exerts its tumor-suppressive effects by regulating *FADD* in breast cancer cells, we next checked the effect of BRCA1 overexpression on FADD-related signaling. As shown in Table 1, FADD is an adaptor molecule involved in FasL receptor-mediated apoptosis. Therefore, we examined whether the BRCA1-mediated *FADD* upregulation can sensitize cells to FasL-mediated apoptosis. Toward this end, BRCA1 was over-expressed in MDA-MB-436 cells, in combination with FasL and siRNA of *FADD*. The cell proliferation assay showed that the FasL treatment inhibited cell growth only with expression of BRCA1, which was reversed by treatment with the *FADD* siRNA (Figure 5A). Western blot analysis confirmed increased FADD expression by BRCA1 expression and efficient knockdown of FADD by siRNA (Figure 5B). These data indicate that BRCA1 expression in MDA-MB-436 cells stimulates FasL-induced cell death signaling via increased FADD levels.

### 2.6. Low Expression of FADD is Correlated with Poor Survival of Triple-Negative Breast Cancer (TNBC) Patients, Whereas the Opposite Occurs in Estrogen Receptor (ER)-Positive Cases

**Figure 6:**
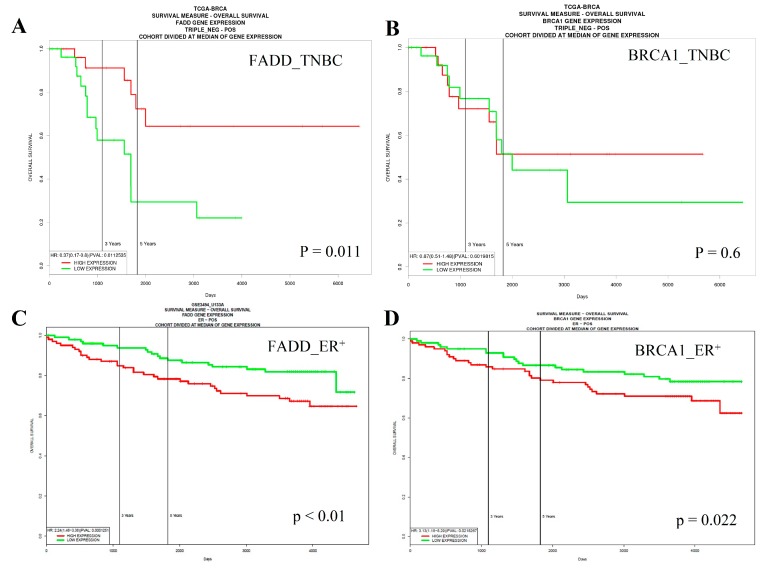
PROGeneV2 analysis showing that low *FADD* mRNA expression is significantly correlated with poor overall survival of TNBC patients. Overall survival curve of TNBC patients grouped according to the (**A**) *FADD* and (**B**) *BRCA1* expression level. Note that a low level (green line) of *FADD* and *BRCA1* expression was correlated with poorer survival, compared to higher level of FADD (red line). Overall survival curve of ER-positive breast cancer patients grouped according to (**C**) *FADD* and (**D**) *BRCA1* expression level. Note that a low level (green line) of *FADD* and *BRCA1* expression was significantly correlated with better survival for ER-positive cancer patients.

Based on our results showing *FADD* as a BRCA1 target, we further examined whether this finding is valid in breast cancer patients. To determine whether the expression level of FADD could be a biomarker for patient survival, we used PROGgeneV2 [16]. As BRCA1 is frequently inactivated in the TNBC subtype, we initially focused on TNBC cases. Figure 6A,B shows that low *FADD* mRNA expression (low FADD, *n* = 14; high FADD, *n* = 7; HR = 0.37, *p* = 0.011) was significantly correlated with low *BRCA1* expression (low BRCA1, *n* = 11; high BRCA1, *n* = 9; HR = 0.87, *p* = 0.60) and poor overall survival of TNBC patients, supporting our results that FADD is a mediator of BRCA1 function in suppressing tumor cell proliferation (Figure 5A). In contrast, the survival curve for ER-positive type breast cancers (Figure 6B) showed that low expression of FADD (low FADD, *n* = 18; high FADD, *n* = 31; HR = 2.24, *p* < 0.001) and BRCA1 (low BRCA1, *n* = 20; high BRCA1, *n* = 29; HR = 3.13, *p* = 0.021) are beneficial for survival, suggesting that the effect of FADD on the patient’s survival differs in a subtype-specific manner.

## 3. Discussion

Many studies have shown the important roles of the tumor suppressor BRCA1 in DNA repair, cell cycle progression, and transcriptional regulation. Thus, substantial effort has been put toward investigating the mechanism of how BRCA1 is involved in these important biological events. Previous reports have identified several genes targeted by BRCA1, but it was unclear whether these expression changes are due to the direct interaction of BRCA1 on the regulatory elements. In this regard, the integration of several genome-wide datasets of ENCODE, TCGA, and GEO can allow for identification of the BRCA1-associated genetic elements for development as putative prognostic markers for breast cancer. Through analysis of ENCODE ChIP-seq data combined with GEO and TCGA data, the present study suggests that BRCA1 expression may directly regulate the expression of a large number of genes.

Known as a multifunctional protein, BRCA1 suppresses tumor formation via a transcriptional co-activator or co-repressor function [17]. Consistent with this, BRCA1 displays a genome-wide impact on gene expression by forming BRCA1-containing complexes. For example, BRCA1 was reported to be associated with ERα protein and repress its signaling via ERα-dependent transcription [18,19]. Moreover, BRCA1 was shown to bind to HDAC2, a chromatin remodeling factor, suggesting it has a function in epigenetic control. Indeed, BRCA1 was reported to modulate the activity of various co-factors within promoter-bound complexes [20].

Three putative BRCA1 targets, *FADD, CKS1B*, and *MEIS2*, were selected among the candidate genes from raw data, along with a related literature review. These three genes play roles in different cell functions such as cell apoptosis, regulation of the cell cycle, and transcriptional regulation. The BRCA1 is known as a multifunctional that also gets involved in those cell functions. Each of the three selected targets is a representative candidate for those three functions. The low expression of these target genes has also been reported in different tumorigenesis events and is considered as important markers. In cell apoptosis, Fas-associated death domain (FADD) is an adapter protein that is recruited to the death-inducing signaling complex (DISC) during signaling via death receptors. Hence, FADD plays an important role in apoptosis in aged lymphocytes and has been reported as a co-tumor suppressor in cancer cell lines [21,22,23]. Previous study results indicate that lack/reduction of *BRCA1* expression may decrease ability in the regulation of apoptosis in breast and ovarian cancers [24]. Moreover, CKS1B is a protein with a well-documented role in cell-cycle regulation that binds to the catalytic subunit of cyclin-dependent kinases and is essential for their biological function. BRCA1 has been shown to have a function relevant to cyclin-dependent kinases elsewhere [25]. Additionally, CKS1B controls the MEK/ERK and JAK/STAT3 pathways [26]. It is worthwhile exploring the effect of BRCA1 on the CKS1B expression. MEIS2 is a homeobox protein; it binds to DNA-bound cofactors such as HOX or PBX proteins and is thought to have a role in transcriptional regulation. MEIS2 was identified in an array screening as one of the top 50 downregulated genes in prostate cancer [27]. Investigation of MEIS2 in low-grade prostate tumors suggested that it plays a critical function related to poor prognosis and might be a useful biomarker or therapeutic target in breast cancer. Most of ChIP signal from ENCODE data analyses fall into 2.5 kb upstream or downstream of the TSS, *MEIS2* was selected as a candidate long-range interacting target gene. In our study, BRCA1 was shown to bind to the region 521 kb upstream of the TSS of *MEIS2*, and, surprisingly, BRCA1 expression could increase the *MEIS2* mRNA level by about two-fold. As we could not observe the promoter activation of *CKS1B* and *MEIS2* or a positive ChIP signal of BRCA1, we suggest that altered post-transcriptional regulation by microRNA or other non-coding RNAs might cause the increase of *CKS1B* and *MEIS2* expression by BRCA1. Indeed, a set of microRNAs has been reported to be regulated by BRCA1 [17], which requires further examination for determination of the possible regulation on the two genes.

The reporter assay showed activation of FADD following the expression of BRCA1 in MDA-MB-436 cells. The overexpression of BRCA1 has been shown to increase Fas and FasL levels in MCF7 cells [28]. The same study showed that a dominant-negative form of FADD could inhibit BRCA1-induced apoptosis. In our study, however, we did not observe apoptosis from BRCA1 expression alone; it only occurred with co-treatment of FasL. We consider that this discrepancy is due to the fact that we used MDA-MB-436 cells, which differ from MCF7 cells in BRCA1 status. Several studies have suggested that BRCA1 may be involved in the processes of apoptosis [29,30,31,32]; however, to our knowledge, the role of FADD in these effects has not yet been reported. Therefore, our results add novel information for understanding the apoptosis mechanism via BRCA1.

Finally, the survival analysis demonstrated that poor prognosis of TNBC patients was associated with a low expression level of *FADD*. Similarly, a recent meta-analysis of head and neck cancer showed FADD as a prognostic marker [33]. Considering that the knockdown of BRCA1 caused reduction of the FADD level, we suggest that the loss of BRCA1 (or its inactivation) in a tumor can cause reduced levels of FADD that in turn desensitizes cells to a FasL-mediated anti-proliferative effect. Interestingly, the correlation was reversed in ER-positive or luminal-type breast tumors. At present, it is not clear why this difference occurs, but we speculate that the presence of BRCA1 in ER-positive or luminal tumors disables the ability to overcome FADD-induced cell death. Taken together, our study suggests a role of BRCA1 in transcriptional regulation, on a genome-wide scale, based on combining ChIP signals from ENCODE and expression data from TCGA and GEO. Further functional studies of putative BRCA1 target genes may help to fully understand the roles of this multifunctional tumor suppressor.

## 4. Materials and Methods

### 4.1. GEO, ENCODE, and TCGA Data Analysis

To verify the BRCA1 target genes, we identified BRCA1-binding sites using the BRCA1 ChIP-seq data of four cell lines (hES, HeLa, HepG2, and GM12878) and calculated the distance from the BRCA1-binding sites to the nearest gene TSS from ENCODE data. To obtain the BRCA1-dependent expression profile, two GEO datasets, GSE30822 [34] and GSE22259, were used. TCGA datasets containing human breast cancer expression profiles were used to calculate the expression correlation between BRCA1 and other genes.

### 4.2. ChIP Assay

ChIP was performed as previously described [6] with minor modifications. In brief, MDA-MB-468 and MCF7 cells were cross-linked with 1% formaldehyde for 10 min at room temperature. The cell pellets were lysed, re-suspended, and subjected to sonication on ice. Five microliters of sonicated chromatin was stored as a 5% input. The remaining samples were immunoprecipitated overnight at 4 °C on a rocking platform using two kinds of BRCA1 antibodies (#1 for Ab-1, MS110, Merck, Kenilworth, NJ, USA, and #2, which is home-made as previously described [6]). Following overnight incubation, ChIP-grade Protein A/G Plus agarose beads were incubated with the lysate for 2 h at 4 °C. The samples were washed with three different buffers and eluted in a buffer containing 5 M NaCl and 20 mg/mL Proteinase K. The immunoprecipitated and input samples were then reverse cross-linked at 65 °C for 40 min. Finally, the elutes were used to detect ChIP signals by PCR, with the primers listed in Supplementary Table S4.

### 4.3. DNA Constructs

Promoters were amplified from genomic DNA isolated from the total DNA of MDA-MB-468 and MCF7 human cell lines. Details of the promoters and primers used for amplification are presented in Supplementary Table S5. Amplified promoters were double-digested by appropriate restriction enzymes and inserted into the pGL3 enhancer vector (Promega, Madison, WI, USA). The selected clones, with the pGL3 enhancer vector alone, were grown in Luria-Bertani medium containing 50 μg/mL ampicillin to extract plasmid DNA. All resulting clones showed sequences that exactly matched with the corresponding NCBI GenBank sequences.

### 4.4. Transfection and Luciferase Assay

The MDA-MB-436 cells, a BRCA1-deficient cell line, were selected for the promoter assay. Transient transfections were performed in 24-well plates using Lipofectamine 2000 (Invitrogen, Carlsbad, CA, USA) in triplicates. As a control, the cells were transfected with 100 ng of pGL3 Basic Vector or pGL Promoter Vector (pGL3-PV) (Promega). To examine the BRCA1 dependency of each promoter, 100 ng of the GFP/WT BRCA1/BRCA1-C61G expression plasmid were co-transfected. At 24/36 h after transfection, the cells were lysed and the activities of *Renilla* and firefly luciferases were measured using Dual-Luciferase Reporter Assay System (Bio-Rad, Hercules, CA, USA). The obtained values of the luminescence for the firefly luciferase were normalized to the values for the *Renilla* luciferase, and the data were corrected for background luminescence, normalized by SV40 promoter activity, and averaged. For each candidate under study, at least three independent transfections were performed.

### 4.5. Knockdown Using siRNA

The BRCA1 or FADD-siRNA was chemically synthesized, along with the scrambled siRNA as a control (Genolution, Seoul, Korea). For siRNA treatments, subconfluent proliferating MCF7 cells were transfected with 50 nM of siRNA using the oligofectamine transfection reagent (Invitrogen, USA). After 24-h incubation, BRCA1 or FADD mRNA and protein levels were measured. To obtain maximal knockdown, we carried out 24-h, 48-h, and 72-h incubations with the siRNA (for sequences, BRCA1 siRNA: CAGCAGUUUAUUACUCACUAAA; FADD siRNA: GAACUCAAGCUGCGUUUAU).

### 4.6. Real-Time PCR Analysis

RNA extraction was performed using TRizol (Invitrogen, Carlsbad, CA, USA) following the manufacturer’s instructions. One microgram of total RNA was used for cDNA synthesis (Superscript First-Strand Synthesis System; Invitrogen) according to the manufacturer's protocol. The expression levels of *BRCA1, FADD, MEIS2* and *CKS1B* were measured by SYBR Green PCR Kit in LightCycler 480 II (Roche Applied Sciences, Indianapolis, IN, USA). Standard PCR conditions were as follows: 10 min at 95 °C followed by 40 cycles of 15-s denaturation at 94 °C, 30-s annealing at 60 °C, and 30-s extension at 70 °C. The primer sequences for PCR are shown in Supplementary Table S6. The human ribosomal protein gene *RPL13A* was used as an internal control gene. Relative quantification was calculated using the 2^−ΔΔ*C*t^ method.

### 4.7. Western Blot Analysis

Western blot analysis was performed as previously described [6]. In brief, cells were lysed in lysis buffer [150 mM NaCl, 1% Triton X-100, 1% sodium deoxycholate, 50 mM Tris-HCl (pH 7.5), 2 mM EDTA (pH 8.0), and 0.1% sodium dodecyl sulfate (SDS)]. Proteins (10–50 μg) were separated by SDS-polyacrylamide gel electrophoresis, transferred to a nitrocellulose membrane, and probed with anti-BRCA1/FADD/MEIS2/CKS1B or FADD downstream antibodies (1:1000, Cell Signaling, Danvers, MA, USA). Equal loading was confirmed with anti β-actin antibody (1:1000; Santa Cruz Biotechnology, CA, USA). Densitometric analysis was performed using the image J 1.47v system (NIH, Bethesda, MD, USA).

### 4.8. AlamarBlue^®^ Cell Proliferation Assay

We performed the cell proliferation assay with alamarBlue^®^ according to a previous study [35]. The cells in a 96-well plate were transfected with the control or BRCA1 overexpression vector and treated with siFADD plus Fas ligand (a gift from Han Seok Choi, University of Ulsan College of Medicine, Seoul, Korea). After 24 h, 1/10th the volume of the alamarBlue^®^ reagent (Invitrogen) was added directly to the culture media. The cells were incubated for an additional 1 h to measure viability, which was detected by a microplate fluorescence spectrophotometer (GenTeks Biosciences, Inc., San-Chong, Taiwan).

### 4.9. PROgeneV2 Analysis

We conducted survival analysis for the FADD signature whose downregulation was consistent with BRCA1 inactivated using PROGgen. We accessed PROGgeneV2 portal to check the patient’s survival. The FADD and BRCA1 survival analyses were inputted as the genes of interest and breast cancer type was selected to create plots. The bifurcation gene expression at median and overall survival measure by death were selected as parameters for analyses. The TNBC in the TCGA breast cancer dataset and ER-positive in GSE3494_U133A dataset were adjusted as the subtypes for the analyses. The statistical method and prognostic plots were calculated by backend scripts as described in the previous report [16].

### 4.10. Statistical Analysis

All data are presented as means ± standard error mean. Statistical significance between two groups was calculated using Student’s *t*-test. *p*-value less than 0.05 were considered to be significant.

## Figures and Tables

**Table 1:** Candidate BRCA1 target genes from ENCODE/GEO/TCGA data analysis.

Gene Name	Function	ENCODE Data (CHIP seq) ^a^	BRCA1 KD/Ctr ^b^	BRCA1 overExp/Ctr ^c^	TCGA Correlation ^d^
CKS1B	binding to the catalytic subunit of the cyclin-dependent kinases and is essential for their biological function	−319	0.66	1.90	0.29
FADD	an adaptor molecule that interacts with various cell surface receptors and mediates cell apoptotic signals	+152	1.06	2.10	0.24
MEIS2	binding to HOX or PBX proteins to form dimers, or to a DNA-bound dimer of these proteins and is thought to play a role in stabilization of the homeoprotein-DNA complex for transcriptional regulation	+521,171	0.77	1.01	−0.32

Note: ^a^ displays the site of ChIP-seq from TSS of targets via ENCODE data analyses; ^b^ shows the targets mRNA expression ratio of BRCA1 knockdown comparing to control from GEO datasets; ^c^ shows targets mRNA expression ratio of BRCA1 overexpressed comparing to control from GEO datasets; ^d^ shows correlation values from TCGA datasets analyses.

## Supplementary Materials

Supplementary materials can be found at http://www.mdpi.com/1422-0067/19/5/1458/s1.
