# Supplementary material for: Integrative Bioinformatics and Functional Analyses of GEO, ENCODE, and TCGA Reveal FADD as a Direct Target of the Tumor Suppressor BRCA1
Source: Int J Mol Sci. 2018 May 14;19(5):1458. doi: 10.3390/ijms19051458 (PMC5983697; doi:10.3390/ijms19051458)
Supplement: Supplementary file 1 [file ijms-19-01458-s001.pdf]

Supplementary Figure 1

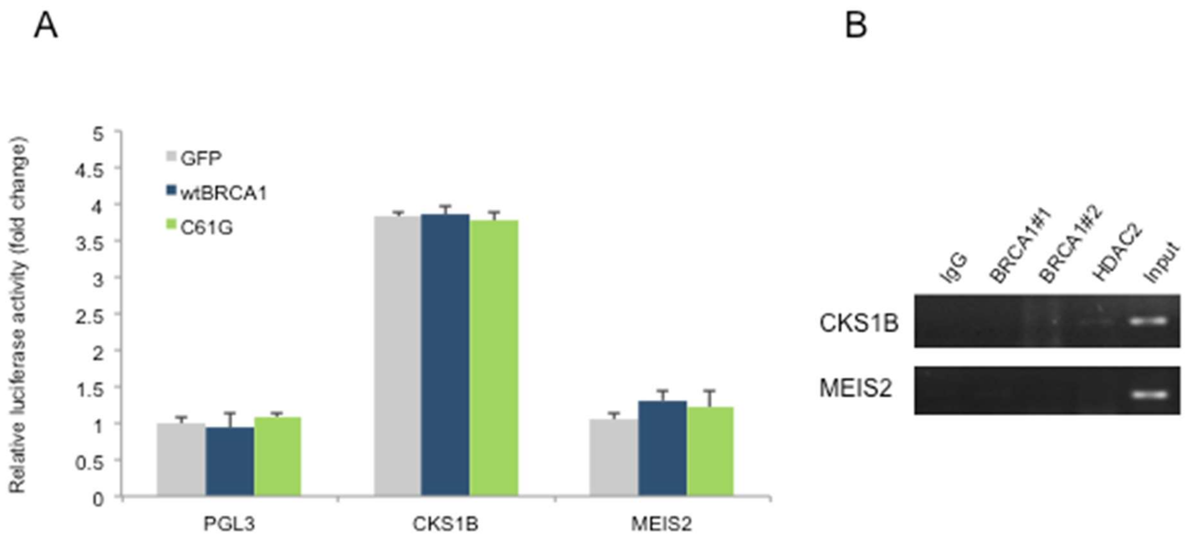

Promoter analysis for the putative BRCA1 target genes. (A) A graph for luciferase reporter assay of the CKS1B and MEIS2 promoter plasmids, in combination with wild type BRCA1 (wtBRCA1) or non-functional mutant (C61G). (B) ChIP results for CKS1B and MEIS2 promoter with two different BRCA1 antibodies and HDAC2. IgG and Input was used as a negative or positive control, respectively.

Supplementary Table 1. The BRCA1 ChIP signal from ENCODE database and BRCA1 dependent expression change from two GEO datasets

Gene Name	ENCODE data (BRCA1 CHIP seq)	GSE22259 Expression data	GSE30822 Expression data
ANPEP	unnamed (-79179)	#N/A	0.140046483
CENPE	unnamed (-76)	0.463340941	0.155474015
ID1	unnamed (-32379), unnamed (+7707)	1.020982905	0.214958903
PNN	unnamed (+161)	0.772059373	0.215856379
SUSD2	unnamed (-24295)	#N/A	0.230809307
LEMD1	unnamed (-63610)	#N/A	0.254516474
DLGAP5	unnamed (-79580)	0.914187251	0.281355563
DARS2	unnamed (-11)	0.9045215	0.289088038
SMC3	unnamed (-361)	1.021103204	0.309860113
TNNI3	unnamed (-3119)	#N/A	0.318065991
FUT8	unnamed (+1870)	0.992421144	0.322867733
MOB1B	unnamed (-154)	#N/A	0.328830217
HMGCS1	unnamed (-283)	1.677479154	0.338263784
CTH	unnamed (-163)	1.30602241	0.341207388
SEPT10	unnamed (+15)	1.04077455	0.345515568
SLC45A3	unnamed (-68696), unnamed (+49304)	#N/A	0.346195509
TAF1B	unnamed (-73)	0.797296768	0.348470135
OXCT1	unnamed (-33516)	0.996978985	0.353844424
KIF15	unnamed (+76)	0.678047851	0.356200772
FBP1	unnamed (-87049)	#N/A	0.36391027
C4orf21	unnamed (-234)	#N/A	0.367754534
HMGB2	unnamed (-1054)	1.137292779	0.368187682
PTGR2	unnamed (+147)	#N/A	0.375853006
IDI1	unnamed (-58)	0.760898277	0.376427911
NSRP1	unnamed (-20)	#N/A	0.378117286
FER	unnamed (+1060)	0.8294352	0.37987163
KIF18A	unnamed (-2078)	0.858023151	0.381624982
PHF3	unnamed (-73235)	0.589242724	0.385235959
CORO6	unnamed (-140127)	#N/A	0.394731959
TTC18	unnamed (-3)	#N/A	0.397570018
STAG1	unnamed (-149)	0.820095844	0.400692437
CUL4B	unnamed (+14495)	0.826892	0.401545178
SFR1	unnamed (+109822)	#N/A	0.404459958
CWC27	unnamed (+80)	0.91259454	0.405328964
FYTTD1	unnamed (-8)	0.566419036	0.410533966
FADS1	unnamed (+84)	1.113099392	0.410780339
CELSR3	unnamed (+8998)	#N/A	0.413508261
MRPS18B	unnamed (-1491)	0.859723503	0.415137504
AHSA2	unnamed (-352)	0.326957844	0.418477842
CREBZF	unnamed (-28)	0.793315308	0.421151257
AGBL2	unnamed (-51902)	#N/A	0.423998893
PCM1	unnamed (-54)	0.693233394	0.427891438
IER2	unnamed (+4941)	1.32968529	0.429742875
LGALS3	unnamed (-77469)	1.073609087	0.431220222
GJC1	unnamed (+55821)	0.765822073	0.431282472

PJA2	unnamed (+661092)	1.004277127	0.433109387
SMARCAD1	unnamed (+96)	0.830872651	0.433959047
STX16	unnamed (-124)	0.783611628	0.437318659
EIF5B	unnamed (-959)	0.546961619	0.44208339
SPOCD1	unnamed (+26565)	#N/A	0.44294245
ZBTB25	unnamed (-1052), unnamed (-23)	0.963847438	0.445669273
TMF1	unnamed (+2)	1.403000802	0.449739795
DARS	unnamed (-101)	0.996569457	0.451375361
MYSM1	unnamed (+137)	1.338821339	0.455914991
TUBA1B	unnamed (+1070)	1.035181245	0.456099714
EFHA1	unnamed (+123)	1.014915078	0.457187082
GNE	unnamed (+18507)	1.178785378	0.460751632
MRPS9	unnamed (+295)	0.957214321	0.46081088
YIPF3	unnamed (-39)	0.974015333	0.462831691
CFL2	unnamed (-158742)	1.071882131	0.465198096
TBC1D5	unnamed (-102)	0.63021023	0.465575013
WNT7A	unnamed (+229497)	#N/A	0.467512187
RANBP2	unnamed (-49), unnamed (+67189)	0.985749145	0.468284624
NEMF	unnamed (-10152)	#N/A	0.470033855
SMARCAL1	unnamed (-243)	1.258806314	0.470929141
DNTTIP2	unnamed (-30)	0.950002933	0.474349306
RBBP8	unnamed (+62)	1.258783826	0.474576323
TTC32	unnamed (+153)	0.83599461	0.4756728
MTHFD1	unnamed (-108)	0.992885456	0.478477648
DALRD3	unnamed (-3517), unnamed (-2835)	0.780682291	0.479377527
MYL12A	unnamed (+96)	0.715193136	0.482497558
DDX27	unnamed (+66)	1.066801662	0.482638644
WRN	unnamed (+453)	#N/A	0.482865316
FZD2	unnamed (+132439)	1.463979329	0.483434728
PIBF1	unnamed (-206)	0.837491244	0.483440865
MYL12B	unnamed (-82)	1.158012413	0.485099694
RPL26	unnamed (+199)	0.942067041	0.488757831
C8orf45	unnamed (+54811)	#N/A	0.489973284
ABHD15	unnamed (-1837)	1.35646249	0.490135984
PTCD2	unnamed (-103)	0.784423362	0.491987581
PIK3C2A	unnamed (-38084)	0.866073062	0.492322837
IFT74	unnamed (+22)	0.980687007	0.493561426
ZNF33B	unnamed (-125)	0.504812323	0.493840564
FKBP3	unnamed (+279)	0.928847661	0.494618435
USO1	unnamed (-431)	1.10033935	0.49992946
SREK1	unnamed (+180)	0.814338842	0.499997287
WDR6	unnamed (+136)	1.030953641	0.50139691
DZIP3	unnamed (+252)	#N/A	0.501446168
TRMT61B	unnamed (-17)	1.202193217	0.502075521
SIRT1	unnamed (-35136), unnamed (-182)	1.345268044	0.502874307
NCOR2	unnamed (-199732)	1.452910334	0.502914155
DHX29	unnamed (-336)	0.88383919	0.504268385
XPO1	unnamed (-108)	0.876640976	0.504425492
RPS6	unnamed (-13)	0.836769321	0.506523013
FANCM	unnamed (-1406)	#N/A	0.507235835
SKIV2L2	unnamed (+281)	0.82493928	0.507854399

MATR3	unnamed (-4)	0.648285837	0.50811514
MTIF2	unnamed (+151)	1.088390892	0.508573501
CCP110	unnamed (-1795)	#N/A	0.508672443
FAM174B	unnamed (-153470)	#N/A	0.508703969
FYCO1	unnamed (-89)	#N/A	0.508784893
ARL6IP6	unnamed (-370), unnamed (+611)	0.982441245	0.509097408
KIAA0196	unnamed (+10)	0.862717371	0.509895147
TMEM41B	unnamed (-137)	1.126248035	0.510039241
CCDC99	unnamed (+231)	1.116666597	0.51345111
METTL4	unnamed (+108)	1.099489708	0.513785132
RPGRIP1L	unnamed (-230)	0.829525311	0.516450805
MKRN2	unnamed (-125)	0.800701455	0.516583401
GCC2	unnamed (+17)	1.019550709	0.517152323
RAB42	unnamed (-10554)	1.34291915	0.518066868
CTR9	unnamed (-104)	0.954013154	0.518450282
DDIT4	unnamed (+61)	0.673959548	0.519209825
IPO11	unnamed (-6228)	0.823808048	0.519552137
TLE2	unnamed (-6539)	0.667176356	0.52176234
DBN1	unnamed (+19014)	0.939822984	0.522444304
NUF2	unnamed (-383)	0.772921169	0.523014843
TLCD2	unnamed (-6054)	0.657502608	0.523092656
ZDHHC3	unnamed (-68)	0.809660159	0.523816835
ARHGEF40	unnamed (+22309)	#N/A	0.524061266
CALM2	unnamed (-118)	0.992701546	0.524316755
COPS2	unnamed (-105)	0.593330326	0.524386166
SENP7	unnamed (-236)	#N/A	0.524436259
EIF1B	unnamed (-150)	0.805460345	0.524645688
KNTC1	unnamed (-477)	0.776829935	0.527817073
TAF1D	unnamed (+432)	0.897045176	0.528445346
COASY	unnamed (-12)	0.865192992	0.529293947
CDNF	unnamed (-204)	#N/A	0.529850135
ZZZ3	unnamed (-834), unnamed (+85)	0.852681176	0.530594634
CBR4	unnamed (+302)	0.884638731	0.532001901
RAB5A	unnamed (-287)	0.98126523	0.533440677
LIG4	unnamed (-2933)	0.997700723	0.533897573
DFNB59	unnamed (+194)	#N/A	0.534070662
CETN3	unnamed (+63)	0.977189771	0.534319884
ZBTB11	unnamed (-200)	0.870179964	0.534561207
GIN1	unnamed (+89)	0.711356152	0.534617816
ATG16L2	unnamed (+189)	#N/A	0.534757444
TGDS	unnamed (+151), unnamed (+46416)	1.225683706	0.534941987
MRPS31	unnamed (-47)	1.083926731	0.535181668
PRPF40A	unnamed (-1043), unnamed (-62)	0.957916058	0.537013979
PPIG	unnamed (+40)	0.966641871	0.537016537
RBBP7	unnamed (-960)	1.088827692	0.537933296
USP8	unnamed (-31)	0.419457672	0.539868384
DUSP13	unnamed (-2151)	#N/A	0.54056628
KIF18B	unnamed (-202)	1.259865988	0.541004972
C15orf23	unnamed (+161)	0.777861394	0.542959881
NCAPG	unnamed (+1)	0.787987203	0.543743932
PIM1	unnamed (-445)	1.359836913	0.544021585

THAP9	unnamed (+45)	1.535761887	0.545715611
RPL14	unnamed (-4366), unnamed (-57)	0.617296484	0.546698833
THUMPD3	unnamed (+113), unnamed (+33517)	0.976501203	0.546705312
TMEM55A	unnamed (-29186)	#N/A	0.547022319
SRSF2	unnamed (-145)	0.76059065	0.547461291
EIF3A	unnamed (+708)	0.85585189	0.550391746
APPL1	unnamed (-149)	0.727878269	0.550765559
KBTBD6	unnamed (-144)	1.010497121	0.552237117
CCNH	unnamed (+295)	0.853542732	0.552630847
NKRF	unnamed (-578)	0.861622649	0.552718387
HEXIM1	unnamed (+987)	1.043847281	0.553962082
OXNAD1	unnamed (-218)	1.323607751	0.554477506
ENOPH1	unnamed (-979), unnamed (-97)	0.903774006	0.555059749
HELLS	unnamed (-327)	0.572755837	0.556497987
PLCD3	unnamed (-663)	#N/A	0.55655551
ZMYM1	unnamed (+75)	0.839394238	0.558220882
DDX5	unnamed (-498)	0.976638597	0.559204873
ZNF518A	unnamed (+228)	1.324832806	0.559989713
RRAGA	unnamed (-103)	1.094485198	0.560586474
MRPS25	unnamed (-24)	0.634050069	0.561591513
C1D	unnamed (-1)	0.792077747	0.561828919
TTC7B	unnamed (+57801)	#N/A	0.563396662
ZC3H15	unnamed (-216)	1.03897568	0.563441268
GPBP1	unnamed (-39982)	0.738343719	0.56373824
MAK16	unnamed (-21)	0.903259655	0.565240025
C3orf23	unnamed (+137)	0.828773823	0.566276787
MNS1	unnamed (+99762)	#N/A	0.566494584
FAM110A	unnamed (+61)	#N/A	0.570633266
CCNJ	unnamed (-13)	1.230814738	0.570978648
CNOT7	unnamed (+1)	0.797735205	0.57499947
OAZ1	unnamed (+4281)	1.29717828	0.575054782
DLG1	unnamed (-211723)	0.670474221	0.575687737
RPL23A	unnamed (+124)	0.973659717	0.577102396
NKAP	unnamed (+43)	0.526640578	0.577392153
CCDC88A	unnamed (-43)	0.433928126	0.57853153
COX15	unnamed (+501)	1.448195544	0.57918922
GTPBP2	unnamed (-604)	1.312319649	0.580047729
RPL36AL	unnamed (-95)	0.983460761	0.581601462
DHX36	unnamed (+75)	0.805997171	0.581972825
PNPLA2	unnamed (+783)	#N/A	0.582135848
KDM1A	unnamed (-124)	0.988486644	0.582883237
H2AFX	unnamed (-101)	1.009357569	0.583110866
DNAAF2	unnamed (+789), unnamed (+1617)	#N/A	0.583373584
DNAJC28	unnamed (+11275)	#N/A	0.586136243
CD2AP	unnamed (-56)	0.955885708	0.586969339
NDUFAF3	unnamed (-222), unnamed (+460)	0.582777657	0.587688474
PARP2	unnamed (-34)	0.663952077	0.589039911
FANCD2	unnamed (-24)	0.705909016	0.589303745
SMC1B	unnamed (+40)	#N/A	0.589367358
CCNE1	unnamed (+33391)	0.829094241	0.590069121
BAZ1A	unnamed (+2082)	1.096976654	0.590344082

TSC22D2	unnamed (-909)	0.928908126	0.590864343
SLC4A3	unnamed (+15092)	#N/A	0.591589473
EIF3H	unnamed (-21)	0.920509219	0.591690767
YME1L1	unnamed (-771)	1.048962947	0.592033911
TKT	unnamed (+58970)	0.992639299	0.593178427
ARL8B	unnamed (-141771)	1.353606027	0.593832907
PPIA	unnamed (-314), unnamed (+884)	0.808171593	0.594921656
TMEM121	unnamed (-35745)	#N/A	0.59634397
PTGIS	unnamed (-43067)	#N/A	0.597117532
GDF9	unnamed (-1809)	#N/A	0.598510855
FZD3	unnamed (+128087)	1.042787866	0.598848377
RAB33B	unnamed (-297)	#N/A	0.599013402
DIS3	unnamed (+320)	1.033020824	0.599374346
RMI1	unnamed (-560)	1.344132005	0.601592815
MMGT1	unnamed (+144)	1.338074122	0.602556865
WSB1	unnamed (-52), unnamed (+39003)	1.075771861	0.604415648
SCFD1	unnamed (-131)	0.955379804	0.605214211
CTAGE6P	unnamed (-144337)	#N/A	0.605342128
ACTN1	unnamed (+162579)	1.131950295	0.605758568
CCDC51	unnamed (+4124)	1.678857295	0.606028992
TPR	unnamed (-326)	0.86860338	0.60608555
TPP2	unnamed (-77)	0.800438231	0.606498601
SMC4	unnamed (-265)	0.647499359	0.607947189
MED20	unnamed (-78)	0.99187481	0.60810807
CCT6A	unnamed (-216)	0.910893592	0.609587138
STT3B	unnamed (-1055)	0.815157048	0.609618745
SLTM	unnamed (+151)	1.200439542	0.60998837
MTR	unnamed (-113)	1.205728809	0.611910818
TCERG1	unnamed (-185)	0.515348289	0.612238228
CCT4	unnamed (-36)	0.924957993	0.614193183
ERI2	unnamed (-59)	1.020140738	0.617612119
CPEB3	unnamed (+79)	#N/A	0.619616105
HEXIM2	unnamed (+572)	0.995343322	0.62214994
CEP95	unnamed (-176)	#N/A	0.623114303
SECISBP2	unnamed (+170)	0.665380487	0.623200623
CUTC	unnamed (-36)	0.776496219	0.623917391
ATR	unnamed (+159)	0.775060481	0.625284115
UBP1	unnamed (-516)	0.851397972	0.625395107
ZNF512B	unnamed (-4098), unnamed (-269)	0.947688108	0.625483038
ABLIM1	unnamed (+131329)	0.886282382	0.626226914
TRAPPC2	unnamed (-190)	1.109132538	0.626391044
MAEA	unnamed (-49)	0.900394812	0.626542602
MRPS22	unnamed (-14412), unnamed (+312)	0.713699	0.626792172
TOMM6	unnamed (+828)	#N/A	0.627536885
CKAP2	unnamed (-4598)	0.959141237	0.628322782
C14orf101	unnamed (-171)	1.344593945	0.628737753
BTBD10	unnamed (-59)	1.493152415	0.629039536
NT5DC2	unnamed (-3005)	1.563907164	0.630846433
C15orf38-AP3S2	unnamed (+18971)	#N/A	0.632093122
DEPDC5	unnamed (-554)	#N/A	0.632877383

HMGCR	unnamed (-487)	0.766300437	0.633548335
MAP1B	unnamed (+200910)	#N/A	0.633601212
MELK	unnamed (-84952)	1.151924065	0.633743982
PPT2	unnamed (+597)	0.928034955	0.635585134
ATP13A3	unnamed (-115680)	0.958448333	0.635672863
RRM1	unnamed (+20)	1.162598329	0.636489608
SCRN2	unnamed (-12)	0.628601513	0.637193045
OXSR1	unnamed (-323)	2.326002947	0.63740867
ANKRD12	unnamed (-204), unnamed (+132)	1.038105395	0.63744039
NOC3L	unnamed (-130)	0.993260861	0.638039567
SHISA4	unnamed (+7427)	#N/A	0.638819871
SF3B14	unnamed (+51)	0.747271865	0.640743975
RALGPS2	unnamed (-182641)	0.775956294	0.641297763
HIST1H2BF	unnamed (-2558)	0.578671983	0.642024809
QRICH1	unnamed (-233)	0.845543866	0.642519068
COPS3	unnamed (-45)	0.822803016	0.643497734
DGUOK	unnamed (-120)	1.096228	0.64389799
TSGA10	unnamed (-235), unnamed (+13102)	#N/A	0.644092238
USP9X	unnamed (-747), unnamed (-58)	0.616951944	0.644890305
PDE12	unnamed (-80)	1.156165543	0.645589853
TNFAIP1	unnamed (+37)	1.065355556	0.645859569
LSM3	unnamed (+33)	0.817387834	0.646054973
WEE1	unnamed (-194)	1.114774759	0.646918906
WDR67	unnamed (+84577)	1.158682305	0.647508107
PRC1	unnamed (-341)	1.05124879	0.647535999
SMC5	unnamed (-15)	1.205583331	0.647686517
MOB3B	unnamed (-44153)	#N/A	0.649141145
PIGW	unnamed (-49)	0.523276006	0.649333671
GOLGA5	unnamed (-35)	1.500982224	0.650750643
FAM200B	unnamed (-23)	1.131760424	0.650889247
C10orf137	unnamed (+75)	0.995990441	0.651341065
RG9MTD1	unnamed (+66)	0.94515859	0.651888235
PAIP1	unnamed (+106)	0.794061182	0.652033
HSDL2	unnamed (-274)	0.751610415	0.652345908
USP39	unnamed (+120)	0.989834103	0.652501727
CPSF3	unnamed (-106)	1.083564013	0.653298843
ATAD2B	unnamed (-47)	0.976007865	0.65435685
TFRC	unnamed (-36)	1.12496452	0.655018462
PRPF38B	unnamed (-55)	0.788021718	0.655868399
YTHDF3	unnamed (-318)	0.926549516	0.656877047
RICTOR	unnamed (-195)	1.006324884	0.657486886
AQR	unnamed (-26)	0.900375297	0.65773268
RANBP9	unnamed (-567)	1.071720358	0.658305641
PRKRA	unnamed (-399)	0.818781832	0.658626273
ACADSB	unnamed (-181)	#N/A	0.65953191
DUSP1	unnamed (-1105)	1.162967827	0.659994809
RAD9B	unnamed (+62)	#N/A	0.660608681
SPG11	unnamed (-75)	1.585953351	0.661074211
DPP8	unnamed (+105)	0.66531666	0.662153577
HOMER1	unnamed (-627)	0.473162244	0.66291685
MTHFD2	unnamed (+24), unnamed (+710)	0.890156971	0.663807096

FOS	unnamed (-61)	1.125695958	0.664306331
SRSF5	unnamed (+339)	0.88460361	0.664418748
METTL15	unnamed (+2026)	#N/A	0.667563048
PCCA	unnamed (-41)	0.810081251	0.667578114
CUL4A	unnamed (-882)	1.004550659	0.668668571
H1F0	unnamed (-2724)	1.011708368	0.669233125
UTP18	unnamed (-368)	0.834016379	0.669452816
ZCWPW1	unnamed (-466)	#N/A	0.66962313
C16orf62	unnamed (+76)	#N/A	0.670638587
TSPYL4	unnamed (-279)	0.944539474	0.6707741
WDHD1	unnamed (-121)	0.943464551	0.67145587
TNFAIP8	unnamed (-367561), unnamed (-285000), unnamed (-87067)	0.715038231	0.672312154
PIP5K1B	unnamed (+74275)	1.070819593	0.672467676
TUBGCP5	unnamed (+179)	0.826154309	0.672533202
PPP2R3C	unnamed (+76095)	0.896417776	0.67312916
KIAA0020	unnamed (-143)	0.884422537	0.673920427
DCP1A	unnamed (-13)	1.144582323	0.673996204
DYNC1H1	unnamed (-16287)	0.973799161	0.67537544
SREBF1	unnamed (+599)	0.973143422	0.675548143
AKAP11	unnamed (-321)	1.084363358	0.676380637
BRIX1	unnamed (-108)	0.831950366	0.676443584
RNF141	unnamed (-67)	0.916754596	0.67672258
HERC1	unnamed (-123)	1.085043102	0.676816898
PRMT3	unnamed (-153)	0.809040239	0.677141901
DPH3	unnamed (+47)	0.860582583	0.677248679
ATAD2	unnamed (-76)	0.748256921	0.677252587
CYP2W1	unnamed (+10109), unnamed (+61060)	#N/A	0.677254935
HELQ	unnamed (-174)	0.641882316	0.677691218
ITGA2	unnamed (-188986)	1.065152207	0.678613083
ASTE1	unnamed (+154)	#N/A	0.680808997
CDKN2AIP	unnamed (-607), unnamed (-66)	1.071199285	0.680853944
ICT1	unnamed (+169), unnamed (+21419)	1.021770179	0.68136022
PLA2G12A	unnamed (+127)	0.612778341	0.68229331
SPC25	unnamed (-56)	0.981208542	0.682520188
KIAA0753	unnamed (+82)	#N/A	0.682621956
ZMYM5	unnamed (+16)	#N/A	0.682727145
PTPN4	unnamed (+59)	1.010328527	0.682786119
MGEA5	unnamed (-241)	0.786560431	0.684261753
ZNF23	unnamed (+21)	1.322691648	0.684432449
TK2	unnamed (-2259)	1.542362282	0.684584658
RPL27A	unnamed (+513)	1.006777096	0.684865893
PPIAL4A	unnamed (-568346)	#N/A	0.685063312
RPL37	unnamed (+181)	0.962108209	0.68665036
EIF1	unnamed (-227), unnamed (+692)	0.996583449	0.688106807
SLC4A1AP	unnamed (+362)	0.842669982	0.688472913
ZCCHC11	unnamed (-346)	0.939225483	0.688998969
FAM156A	unnamed (+60626), unnamed (+74318)	#N/A	0.68955012
BBS1	unnamed (-29)	0.648261067	0.690207484
MBD4	unnamed (-158)	1.077709662	0.690374697
ZNF133	unnamed (-94)	0.926834804	0.690872183

WDR36	unnamed (+94)	0.870261017	0.691114996
TROAP	unnamed (-100)	0.659847693	0.695567156
HNRNPH3	unnamed (+467)	0.998370421	0.696605365
TRAPPC10	unnamed (-73092)	0.69193472	0.697171421
DENR	unnamed (+11)	0.968193233	0.697290447
HAUS5	unnamed (-17)	0.884621392	0.697422094
RPLP2	unnamed (-98)	1.036210176	0.697527185
ZNF323	unnamed (+48)	0.903857365	0.697672633
PSAT1	unnamed (-94)	0.826933945	0.697910026
CRNKL1	unnamed (+3487)	1.061453972	0.698259478
CCBL2	unnamed (+301)	0.872062182	0.698679332
CDCA5	unnamed (-14)	1.390607443	0.699854173
TTC14	unnamed (-51)	0.790163814	0.700139248
TADA3	unnamed (+53)	0.505640214	0.700774039
GORASP2	unnamed (+445), unnamed (+232353), unnamed (+232918)	1.037286407	0.700939313
RAB6A	unnamed (-18440), unnamed (-271)	1.409060393	0.701582863
MRPL16	unnamed (+189)	0.766159174	0.702210055
DNAJB14	unnamed (-3707)	0.994678281	0.70237078
PRKRIR	unnamed (+61881)	1.007234544	0.702581044
COQ2	unnamed (+181)	0.787695839	0.703970364
ZNF444	unnamed (-147)	1.273113855	0.704133955
EIF4EBP1	unnamed (+453)	0.661263015	0.704703097
HCFC1R1	unnamed (+76)	0.936786116	0.705069437
FAM69B	unnamed (+15655)	#N/A	0.705455158
CANX	unnamed (-562)	1.263417225	0.70605614
MIPEP	unnamed (-49)	0.887791671	0.706629338
CUL3	unnamed (-154)	0.838752425	0.706936407
CEP76	unnamed (-351)	0.999761604	0.707142352
GLOD4	unnamed (-20)	0.914093964	0.707426111
GORASP1	unnamed (+8)	1.409488868	0.707641049
TLK1	unnamed (+69958), unnamed (+70523)	0.830690632	0.70779084
SMCR7	unnamed (-328)	#N/A	0.707978019
APBB2	unnamed (-2169)	0.802205114	0.708738856
DTNBP1	unnamed (+414203)	1.057845462	0.7087888
FANCC	unnamed (+313)	0.809432864	0.709387456
RPL4	unnamed (-248)	0.893478417	0.710377673
ZNF143	unnamed (-338)	0.979905036	0.710792572
INTS9	unnamed (-304)	1.114242708	0.711078735
MED4	unnamed (-79)	0.943732224	0.711275247
SMEK2	unnamed (+184)	0.431097971	0.71162745
VPRBP	unnamed (+43)	0.876278085	0.711955023
NLGN2	unnamed (-3536)	0.758746358	0.712118578
LOC81691	unnamed (+87)	#N/A	0.713772094
RPS29	unnamed (-140)	0.910742051	0.714123069
C20orf30	unnamed (-227)	1.559531692	0.714346765
CCNG1	unnamed (+351)	1.400324259	0.714523072
HACL1	unnamed (-326)	0.991150674	0.714681506
MRPL44	unnamed (-119)	0.714985654	0.715282037
MTMR6	unnamed (-135)	0.80076399	0.715500114
SPTY2D1	unnamed (+74)	0.52544815	0.715800426

MTMR4	unnamed (-627)	1.212202678	0.715848754
HNRNPD	unnamed (-498)	0.824295345	0.715882474
ARHGEF7	unnamed (+38290)	1.321688052	0.71622481
ABCE1	unnamed (+151)	0.773995771	0.716264835
LRR1	unnamed (+515)	#N/A	0.716768589
HAT1	unnamed (+4)	0.908108976	0.716860716
HAUS2	unnamed (+94)	0.746357467	0.717315952
FRMD6	unnamed (-314)	1.542497761	0.718164721
CDCA2	unnamed (+51)	0.774337984	0.718208836
PSMD10	unnamed (+196)	0.939936925	0.718353626
LIAS	unnamed (+4)	1.293453666	0.718865341
SNX5	unnamed (-420)	0.549081367	0.719680191
LBR	unnamed (-47024)	0.879310497	0.720411553
ARL6IP1	unnamed (-195)	1.079195648	0.720800617
B9D1	unnamed (-154)	0.627791004	0.721554856
B9D1	unnamed (-154)	0.627791004	0.721554856
DHX35	unnamed (+87912)	1.10280272	0.722278902
UBE2D3	unnamed (-351)	0.914077898	0.723466439
SOCS4	unnamed (+96), unnamed (+24622)	0.899222824	0.723571211
METTL8	unnamed (+44)	0.792996741	0.724334752
MUT	unnamed (-50)	1.011241805	0.724369543
GUF1	unnamed (-21)	0.765373382	0.724395116
MARCH7	unnamed (-106)	1.022073382	0.724450987
RNMT	unnamed (-56)	0.705129454	0.724974756
GPATCH8	unnamed (-61)	1.365143668	0.725019421
BDH1	unnamed (+45688)	#N/A	0.725092711
MED9	unnamed (-3)	0.856950572	0.725215438
PCNX	unnamed (+412821)	0.989476521	0.7260609
THBS1	unnamed (+201861)	1.617279264	0.726174589
ZDHHC4	unnamed (+20)	0.790844427	0.726761207
CRELD1	unnamed (-26)	#N/A	0.72718385
NUSAP1	unnamed (-345)	0.798975192	0.727273443
INSIG2	unnamed (-74132)	1.217498384	0.727675907
CDK12	unnamed (+14)	0.84156348	0.727896227
DCTN4	unnamed (+2)	0.858653012	0.728032624
SLC25A16	unnamed (-41)	0.690543074	0.728369597
TIPIN	unnamed (+75)	0.945324367	0.728505737
SRSF11	unnamed (+102)	0.758656963	0.729666252
BZW1	unnamed (-687)	1.429712162	0.730591939
TTLL5	unnamed (-65)	1.14874749	0.730931283
CCNB1	unnamed (+262)	0.979156753	0.731138728
MYH10	unnamed (-293)	0.694925651	0.73148526
ATG7	unnamed (+154)	0.802489099	0.731674515
XPC	unnamed (-89)	1.442735824	0.731909348
NOLC1	unnamed (+43)	0.57020477	0.732122888
NOD1	unnamed (+111)	#N/A	0.732396201
LOXL3	unnamed (-515)	#N/A	0.732683414
WBP4	unnamed (-101)	0.810310535	0.733387106
RAB28	unnamed (-228)	1.10507094	0.735420476
H3F3C	unnamed (+63116)	#N/A	0.73586205
COX11	unnamed (+148)	0.647184745	0.73626298

UTRN	unnamed (-75398)	0.605569626	0.736523444
TMEM107	unnamed (-21)	0.557681365	0.738846207
ZZEF1	unnamed (-706)	1.342918799	0.740534783
FAM173B	unnamed (-35)	0.726941229	0.740954488
STXBP4	unnamed (-210)	#N/A	0.741150116
ZRANB3	unnamed (+359)	#N/A	0.741556417
PXN	unnamed (-26099), unnamed (+36969)	1.536810816	0.741822397
AURKA	unnamed (-236)	0.815698387	0.742050734
SRP54	unnamed (+63320)	1.205938995	0.742618148
ATG14	unnamed (+140600)	1.081684579	0.742934579
CPSF2	unnamed (-152)	0.821757561	0.74336067
HIBADH	unnamed (-9)	0.986623131	0.743579705
CLTC	unnamed (-68)	1.067033329	0.744577421
TUFM	unnamed (-92)	0.987113292	0.744792965
KPNB1	unnamed (-106)	0.879461119	0.745379129
ANAPC4	unnamed (+25)	1.16469554	0.745403208
C20orf94	unnamed (-403)	#N/A	0.74551679
NOL11	unnamed (+75)	0.858623718	0.745780494
ST3GAL2	unnamed (+92267)	0.823414081	0.745831798
CAMK2B	unnamed (-165493)	#N/A	0.746044446
PSMG2	unnamed (+67)	1.009450581	0.74677701
TRAM1	unnamed (+20)	1.111627814	0.747176851
BRE	unnamed (+536)	1.148843706	0.747300577
TMSB10	unnamed (+9), unnamed (+20361)	0.994305583	0.747838693
SKA3	unnamed (+35)	0.904136208	0.748627508
PPM1B	unnamed (-645)	0.785777059	0.749263316
FAM114A2	unnamed (+181)	0.654749609	0.749691123
LIN54	unnamed (-2461)	#N/A	0.750965559
PRRT1	unnamed (-2106)	#N/A	0.751583313
ANKRD13C	unnamed (-79)	0.785319131	0.751792749
C5orf28	unnamed (-296)	0.960322652	0.751921337
NCAPG2	unnamed (-7)	0.501388226	0.75252153
GTF2H1	unnamed (-21)	0.84553698	0.752669202
CHUK	unnamed (-45)	1.17381483	0.7528108
DIP2C	unnamed (+178480)	#N/A	0.75303349
RFC5	unnamed (-43)	0.690231376	0.7533329
HSPA12B	unnamed (+35656)	#N/A	0.753375996
TMEM161B	unnamed (+33)	1.041462524	0.753665514
SIK2	unnamed (-263)	0.783522915	0.754126524
NMD3	unnamed (+62)	0.864871917	0.754363744
SEC62	unnamed (-126)	1.026519604	0.755264502
C9orf95	unnamed (+59618)	0.846169109	0.755440402
MAML2	unnamed (+99737)	#N/A	0.75606163
EIF5	unnamed (-178)	0.704567	0.756354962
C11orf58	unnamed (-247)	0.950711536	0.756373861
FBXW11	unnamed (-433)	0.971147089	0.756425788
SLC25A1	unnamed (+7172)	0.906071584	0.756613943
GGPS1	unnamed (+207)	0.836076184	0.757179069
LUC7L3	unnamed (+89)	0.77447924	0.75735017
KPNA2	unnamed (+42)	0.930319757	0.757417768
MTHFD2L	unnamed (-298)	0.526224393	0.757539691

FLII	unnamed (-2041)	0.76766339	0.757868489
ADAM11	unnamed (+15790)	#N/A	0.758551143
PLK4	unnamed (+426)	0.658920625	0.758648162
SLC39A13	unnamed (-64)	0.877163366	0.759269869
UPF3B	unnamed (-3)	1.146574505	0.759281673
ASPH	unnamed (-320)	0.589036449	0.759307961
PHF7	unnamed (-477)	#N/A	0.759362981
ECD	unnamed (+24)	1.07188261	0.759685201
TTC27	unnamed (-32)	0.978439359	0.759787934
RPP14	unnamed (+116)	1.157217633	0.760293627
RPP14	unnamed (+114)	1.157217633	0.760293627
SVIP	unnamed (-149)	0.550453612	0.761163448
RANBP1	unnamed (-268)	0.887198027	0.762814613
CGRRF1	unnamed (-74)	0.936473743	0.762899543
SNAPC3	unnamed (-75)	0.672063606	0.762910771
C9orf102	unnamed (+61)	#N/A	0.762919549
SCAMP5	unnamed (-73)	#N/A	0.762985774
PPA2	unnamed (+126)	0.986302788	0.763006445
MGAT2	unnamed (-45), unnamed (+12842)	1.308859633	0.76441095
KLF14	unnamed (-373421)	#N/A	0.765528705
MCM4	unnamed (+669)	1.024882289	0.765678528
ZCCHC9	unnamed (-84)	1.501232932	0.765788075
GABPA	unnamed (+170), unnamed (+978)	0.604322743	0.766336729
ARFIP2	unnamed (-9)	0.878648982	0.766731368
ANKFY1	unnamed (+42)	1.257752869	0.76705008
PRKCD	unnamed (+35937)	#N/A	0.76729994
SLC15A4	unnamed (-35)	1.206478627	0.767447209
PIM3	unnamed (+114702)	0.999521911	0.767841338
C1orf9	unnamed (-347)	1.247231553	0.768102479
CLK1	unnamed (+40)	#N/A	0.768464604
C10orf76	unnamed (-140)	0.838386974	0.768975267
SFXN2	unnamed (-683)	#N/A	0.769228864
HIRIP3	unnamed (-2)	#N/A	0.769310258
TRAPPC1	unnamed (-222)	1.021176878	0.769665422
DMTF1	unnamed (-137)	0.875571419	0.770347234
PLEKHH3	unnamed (-1537), unnamed (-187)	#N/A	0.771703297
FAM54B	unnamed (+269)	#N/A	0.771848563
SPATA7	unnamed (+236)	#N/A	0.772675271
HSPH1	unnamed (-449)	1.333074624	0.772688823
VPS36	unnamed (-134)	0.96245732	0.772866353
ZNF217	unnamed (-475900)	1.036030028	0.773009063
RAD52	unnamed (-318)	0.876017212	0.773609864
PPIL4	unnamed (-68)	0.900269541	0.773650645
EIF3L	unnamed (-4807), unnamed (-540)	0.842362533	0.773944124
DDX18	unnamed (+199663)	0.666775114	0.774303613
NKIRAS1	unnamed (-1)	1.078351429	0.774329596
TPT1	unnamed (-73)	0.975708885	0.774896954
ATP5E	unnamed (-271)	0.878283097	0.775980749
FCHSD2	unnamed (-386)	#N/A	0.776726525
HPS4	unnamed (-4423)	1.041416374	0.776813414
DDX50	unnamed (-465)	1.185213635	0.777356228

METTL14	unnamed (-93728)	0.993037541	0.777911134
UIMC1	unnamed (-62)	1.132316708	0.777918348
IARS	unnamed (-24)	0.995633088	0.777927294
WDR44	unnamed (-12)	1.444637379	0.778264392
GLI4	unnamed (+11466)	#N/A	0.778515555
AZI2	unnamed (+255)	0.499344839	0.778571057
RPL26L1	unnamed (-21)	0.789650312	0.778797698
MCCC2	unnamed (-149)	0.862461666	0.78041348
ZMYND11	unnamed (+331194)	0.406736863	0.78045211
FASN	unnamed (-114659)	0.702532467	0.780924338
STXBP3	unnamed (+10)	1.751816416	0.781257772
MKKS	unnamed (-682)	0.744636939	0.781495526
HIST2H2BE	unnamed (+1120)	1.658631727	0.782656796
USP47	unnamed (+476)	1.399529407	0.782931538
NKTR	unnamed (-188)	0.729318009	0.783146418
SLU7	unnamed (-129)	0.977384485	0.783213472
MRP63	unnamed (+334)	0.687952262	0.783605325
CYP51A1	unnamed (-354)	0.818688318	0.784173142
BCLAF1	unnamed (+217)	0.783739577	0.784330144
GSTO1	unnamed (-22717)	1.156517775	0.784439501
ADNP	unnamed (-723)	0.881751889	0.784667795
WAPAL	unnamed (+144375)	0.669776599	0.785875511
SNRPB	unnamed (-49)	1.080068614	0.785959455
ABCA7	unnamed (-20)	0.758955556	0.7863919
SEC13	unnamed (-3)	1.576229158	0.78651349
RPL23AP32	unnamed (+29189)	0.790316228	0.786899167
TOPORS	unnamed (+1646)	1.022618686	0.786999467
REV3L	unnamed (-606)	0.660124851	0.787017572
RPS15	unnamed (+29)	1.103779564	0.788530772
SMU1	unnamed (+38)	0.918036672	0.788565376
CENPO	unnamed (-332)	0.59827481	0.788687002
LIPT1	unnamed (+4)	0.890430288	0.789511113
ATP9B	unnamed (-169)	1.305226497	0.790389737
ZNF184	unnamed (+193)	#N/A	0.790730983
MRPS18C	unnamed (+81)	1.033334292	0.791525799
DNAJB11	unnamed (-2716), unnamed (+400)	1.038286695	0.791535147
GBAS	unnamed (+69672)	0.450347693	0.791773416
LPXN	unnamed (-2432)	0.516015225	0.791970106
SGSM2	unnamed (-940), unnamed (+62306)	0.735359684	0.792524792
SUPT7L	unnamed (-251)	1.082126949	0.792837955
ABHD13	unnamed (+52)	0.675672032	0.792913685
FBXO4	unnamed (-21049)	0.801011023	0.792994067
SREBF2	unnamed (-320)	0.996856742	0.793120821
DHODH	unnamed (+138)	0.799888742	0.793143522
TMEM111	unnamed (-15)	1.059396546	0.793257155
LOC100506012	unnamed (+350)	#N/A	0.793531498
POLR1B	unnamed (+419)	0.76083426	0.793795759
QKI	unnamed (+1629)	1.085574269	0.794376764
HDAC2	unnamed (-111), unnamed (+112044)	1.16961926	0.794596538
RNF103	unnamed (+60821)	1.314039779	0.794757865

C20orf29	unnamed (-24)	0.850212142	0.795816436
RALGAPB	unnamed (-37463)	0.809141881	0.795960896
AP4E1	unnamed (-77)	1.068734015	0.796824914
NUFIP1	unnamed (-88), unnamed (+71099)	0.820487468	0.796882789
RALA	unnamed (+109811)	1.524062506	0.797007246
RBM26	unnamed (-541)	0.778762603	0.797547952
ING1	unnamed (-1605)	1.091849361	0.798012443
RAD51B	unnamed (+973787)	#N/A	0.798986751
SIRT4	unnamed (-10451)	#N/A	0.801310611
EIF4A3	unnamed (-145)	1.620702783	0.801574168
SERP2	unnamed (-494278), unnamed (+61696)	#N/A	0.802810371
AP3B1	unnamed (-204)	0.822700336	0.802817707
CLK3	unnamed (-50)	1.090518179	0.803045925
TUBGCP6	unnamed (+21)	#N/A	0.803239629
ASH2L	unnamed (+51)	0.991968456	0.803538336
PSD2	unnamed (+173118)	#N/A	0.803861775
DPEP2	unnamed (+6009)	#N/A	0.803884178
CHMP1B	unnamed (-250)	0.817975385	0.803886249
HPDL	unnamed (-22839)	1.309290894	0.804139418
PAICS	unnamed (-307)	0.643779984	0.80469208
MAPK6	unnamed (-3)	0.685325375	0.80530789
DDX46	unnamed (+87032)	0.957473183	0.805702439
GMPPA	unnamed (+150)	1.453203398	0.806489579
KIAA0284	unnamed (-49189)	#N/A	0.807255821
RNFT2	unnamed (-42), unnamed (+81255)	#N/A	0.807354229
RPL10A	unnamed (+51)	0.755738985	0.807419837
SRRT	unnamed (-54), unnamed (+558)	0.894273245	0.807803463
UCHL3	unnamed (-261)	0.991832261	0.807858976
SCAF8	unnamed (-150)	#N/A	0.808362055
MGAT4A	unnamed (-410496)	#N/A	0.808460776
RANBP6	unnamed (-49)	0.769639349	0.809440105
TRAPPC6B	unnamed (-4914)	0.987129025	0.809837464
TBX6	unnamed (-48)	#N/A	0.810038992
SBK2	unnamed (+33063)	#N/A	0.810718894
IMPDH2	unnamed (+87)	0.802793187	0.810887822
HTRA2	unnamed (+424)	0.760044294	0.811369455
UBR3	unnamed (-2537), unnamed (-80)	0.924846441	0.811790587
HSPA9	unnamed (-116)	1.099248913	0.811921644
FTO	unnamed (+126)	0.629206784	0.812251453
RAB11FIP5	unnamed (-64464)	1.294943538	0.812776221
IKBKAP	unnamed (+223)	0.9129211	0.813544646
KIAA1033	unnamed (-107)	1.442516702	0.814113182
OSBPL11	unnamed (+121)	1.46061204	0.814140287
TMEM66	unnamed (+122)	0.933963	0.814305639
SKP2	unnamed (+296)	0.869667648	0.81461694
SMNDC1	unnamed (-57), unnamed (+93716)	0.984532855	0.815338331
CSTF1	unnamed (+160)	0.800661087	0.815494867
TRIM37	unnamed (-114)	1.41710845	0.815509723
TTC23	unnamed (-1610)	1.094566067	0.815544922
CYB5B	unnamed (-47)	0.993038894	0.815548391
DUSP10	unnamed (-723480)	1.918064863	0.815557541

MKL2	unnamed (-78)	0.701696582	0.815675134
KCTD9	unnamed (-580)	0.707047415	0.815921757
MAPK7	unnamed (-671)	#N/A	0.816477028
UHRF2	unnamed (-50)	1.447092267	0.818198746
RBM39	unnamed (+131)	0.687465385	0.818430815
GLRX3	unnamed (-25565)	0.756868154	0.818479568
ZNF623	unnamed (-13865)	#N/A	0.818655801
CEP57	unnamed (-264)	0.620283604	0.818672077
GGA3	unnamed (-50)	#N/A	0.819353319
TXNDC5	unnamed (-216)	#N/A	0.819379862
SC5DL	unnamed (-167)	0.541361978	0.819661114
EBF3	unnamed (-146983)	#N/A	0.819992178
UBE2A	unnamed (-9062)	0.940783488	0.820057734
NDUFV2	unnamed (+62)	1.23758697	0.820205257
PARD6B	unnamed (+686)	0.963500511	0.820248805
MEF2A	unnamed (-59)	0.654473727	0.821993332
YTHDF2	unnamed (-305)	1.054985111	0.822673392
XPO7	unnamed (+90639)	1.023346242	0.823161709
KRIT1	unnamed (+161)	0.928568416	0.823465885
PARN	unnamed (-2422)	1.617651508	0.824207905
BAZ1B	unnamed (-157), unnamed (+495)	1.004679669	0.824271599
RPL22L1	unnamed (+332)	0.365631039	0.824303963
NEIL3	unnamed (+107)	0.968159883	0.826070589
LARP7	unnamed (-227)	0.941979979	0.82607527
C17orf61	unnamed (-516)	0.741312325	0.826143495
BOD1L	unnamed (-178)	1.122694352	0.826210861
C14orf133	unnamed (-195)	#N/A	0.82645034
TRNT1	unnamed (-47)	0.923216818	0.826469456
RPA3	unnamed (+77836)	0.879300097	0.826522341
PER1	unnamed (-1896), unnamed (+13300)	0.718623252	0.827206708
KCNAB3	unnamed (-2736)	#N/A	0.827309298
HPS5	unnamed (-74)	1.204908436	0.82781341
PIDD	unnamed (-4588)	#N/A	0.828488908
ACTB	unnamed (-323), unnamed (+526), unnamed (+4467)	1.161459779	0.828678428
PPAT	unnamed (-160)	0.895385882	0.829513814
ATP11A	unnamed (-83800)	0.883199049	0.829868056
IFT80	unnamed (+155)	0.800270754	0.83008283
C11orf51	unnamed (-31)	0.729741	0.830455668
NUDT18	unnamed (+360)	#N/A	0.830974855
TRIO	unnamed (+1111), unnamed (+520535)	0.994853519	0.831907488
MBNL1	unnamed (-30189)	0.813370006	0.831913483
CDC26	unnamed (+45)	1.051349968	0.832100835
PCID2	unnamed (-20)	0.906974247	0.832770564
POLR2B	unnamed (-1214)	1.067610158	0.833202286
EML6	unnamed (-166601)	0.795015666	0.833249791
DUT	unnamed (+641)	0.668240752	0.833268229
TIA1	unnamed (+50), unnamed (+105964)	0.829398251	0.833420471
DSE	unnamed (-155)	#N/A	0.833763129
NUP54	unnamed (+4)	0.89575	0.833850658
MIER3	unnamed (-221965)	1.094902939	0.833946614

TMEM89	unnamed (-41794)	#N/A	0.834297964
AGBL5	unnamed (-597)	0.758549572	0.834871872
VRK1	unnamed (-44)	1.098289205	0.834938058
GALNT9	unnamed (+267297)	#N/A	0.834956505
AP1G1	unnamed (+9)	0.883013627	0.835074288
C12orf73	unnamed (-104)	0.379867182	0.835147926
RGMA	unnamed (+169708)	#N/A	0.835185657
CHMP7	unnamed (+2954)	0.656235589	0.835232687
TMPRSS4	unnamed (+68442)	#N/A	0.835451615
PRKDC	unnamed (-689)	0.847463669	0.835866662
NHEJ1	unnamed (+565)	1.012328946	0.836762974
ELL2	unnamed (-211)	1.060748844	0.83700977
AGL	unnamed (-1)	0.927256252	0.837915972
HIGD2A	unnamed (-79)	0.964499077	0.837922448
FDXACB1	unnamed (+304)	#N/A	0.838108304
RAPGEF6	unnamed (-53)	0.956256021	0.83825787
TSEN34	unnamed (-871)	1.041708091	0.838365612
SETD3	unnamed (-558)	0.901695641	0.838825569
CD5	unnamed (+27530)	#N/A	0.83899354
NUDT15	unnamed (+455)	1.025906942	0.839083107
NDC80	unnamed (-129)	1.009715112	0.839169432
CLCN2	unnamed (-1854)	#N/A	0.83949065
RCOR1	unnamed (-467)	0.881179415	0.840208998
SOX12	unnamed (-340)	#N/A	0.840411084
LMBRD1	unnamed (+175)	1.079037736	0.84070906
TAF13	unnamed (-107)	#N/A	0.840988337
COMMD6	unnamed (-278)	1.323545305	0.841028703
NUP153	unnamed (-319)	1.063693782	0.84140076
ATP5J	unnamed (-342), unnamed (+466)	0.905934341	0.841934503
RAB34	unnamed (-1838)	0.855407662	0.841973171
NRBF2	unnamed (-153)	1.085659512	0.841982348
SHPRH	unnamed (-187)	1.081841721	0.842354128
CIAO1	unnamed (-106)	0.944377808	0.842404723
TSC1	unnamed (-83)	1.196538443	0.842764899
ZNF286A	unnamed (-8)	#N/A	0.842842757
CSTF3	unnamed (-13)	0.538659618	0.844914939
LRPPRC	unnamed (-79)	0.91622557	0.845042365
KBTBD7	unnamed (+3)	0.771425227	0.845163516
UQCRC2	unnamed (-129)	0.73452194	0.845491105
RNF167	unnamed (-28)	0.875651248	0.845673657
NCOA4	unnamed (+30)	1.179030993	0.845727015
UBB	unnamed (-32), unnamed (+495)	0.963277571	0.846037414
SFI1	unnamed (+177)	2.317379627	0.846048497
MARCH5	unnamed (-124)	1.117197451	0.846587309
QTRTD1	unnamed (-10)	1.113213643	0.846907083
CETP	unnamed (+2786)	#N/A	0.846920664
BIRC2	unnamed (-176)	1.590082161	0.847332128
KIF24	unnamed (-9)	#N/A	0.847517888
C12orf49	unnamed (-211)	0.517450437	0.848188346
C15orf24	unnamed (-148)	1.001594349	0.848447314
RPIA	unnamed (-93)	1.095040485	0.848511449

ENDOV	unnamed (+39490)	#N/A	0.848615516
PDCD10	unnamed (-999)	0.755498682	0.849009231
MST1P9	unnamed (-140404), unnamed (+120013)	#N/A	0.849398078
NUCB2	unnamed (-68849)	0.844711767	0.849402775
TFB2M	unnamed (+13)	0.806469898	0.84945477
FRS3	unnamed (-159)	#N/A	0.850464231
C6orf57	unnamed (-153887)	#N/A	0.851050883
NUP98	unnamed (-82)	0.624761594	0.851107372
CAMK2G	unnamed (+94)	0.626843276	0.851214752
C11orf54	unnamed (-522)	1.388044293	0.851309826
HHLA3	unnamed (+3)	1.00880746	0.851422998
NRAS	unnamed (+115)	0.594167771	0.851988145
TMEM101	unnamed (-71)	1.003616735	0.852462319
ING3	unnamed (+264)	0.462712083	0.852732186
MBTD1	unnamed (-102)	0.796826351	0.852793328
CRBN	unnamed (+21)	0.69276057	0.853383668
INO80	unnamed (-499)	0.829662039	0.853548038
CHRAC1	unnamed (-46783), unnamed (-120), unnamed (+885)	0.537878158	0.85362075
C7orf11	unnamed (-76)	0.910415733	0.854191311
GLB1L	unnamed (-91)	#N/A	0.854450643
TRIP4	unnamed (-32)	1.226400806	0.854638123
KIAA0232	unnamed (-164)	1.210812495	0.854679622
HHIPL2	unnamed (+82448)	#N/A	0.854886565
MTMR14	unnamed (-135)	1.077433764	0.854910329
ATP5G2	unnamed (+192)	0.95476575	0.855046447
CTSH	unnamed (+71226)	#N/A	0.855133734
YWHAQ	unnamed (-179)	0.841797377	0.855288428
GNPTG	unnamed (+53)	0.928376434	0.855422912
USPL1	unnamed (-80)	0.841364274	0.855888221
GOLGA1	unnamed (-31)	1.309172651	0.855990593
UNK	unnamed (+330)	0.919856277	0.856362604
C3orf75	unnamed (+65)	0.871591478	0.857408299
TAS2R42	unnamed (+15247)	#N/A	0.857839173
CCNL1	unnamed (-14492), unnamed (+133)	1.043310458	0.857845793
MASTL	unnamed (+367)	1.416621785	0.857864987
HDDC3	unnamed (-40)	1.146313004	0.85845181
TRIM33	unnamed (-187)	0.880543756	0.858469713
ZNF426	unnamed (-21)	#N/A	0.858684762
DAK	unnamed (+340)	0.867758396	0.858732555
ZNF721	unnamed (+444)	0.601204408	0.858825411
ANKH	unnamed (+207523), unnamed (+726947)	1.000734801	0.859799593
RNF169	unnamed (+618)	0.939246284	0.859857593
ANO8	unnamed (-3)	#N/A	0.860377045
COPB2	unnamed (-10)	1.204877122	0.861117093
PRKAB1	unnamed (-140)	0.701073286	0.861288793
BDP1	unnamed (+11)	0.759150083	0.861809078
BMPR1A	unnamed (-518)	0.579348057	0.862007751
NEURL2	unnamed (+2)	#N/A	0.862142663
CACNA1A	unnamed (+351051)	#N/A	0.862298921
DNAJC25	unnamed (-179)	1.106182625	0.862406375

FRYL	unnamed (+570)	0.70331358	0.862680289
PPM1G	unnamed (-317)	0.974668021	0.862741387
IGSF6	unnamed (+53158)	#N/A	0.863242944
CYB5D2	unnamed (+497)	0.837001994	0.863333198
PREPL	unnamed (+43)	0.511965032	0.863548848
MICAL3	unnamed (+22986)	1.415984471	0.863894138
NSUN3	unnamed (+120)	0.993133339	0.864269186
PPP1CC	unnamed (-159)	0.883578347	0.86431334
CASC4	unnamed (-339)	1.046575458	0.864903377
DOK1	unnamed (+65)	#N/A	0.864918907
ARF6	unnamed (-30045), unnamed (+320)	0.989676884	0.865050697
NOA1	unnamed (-69)	#N/A	0.865701217
MDH1	unnamed (-414)	0.886335559	0.865933228
CEP350	unnamed (-94)	1.023883511	0.866260337
IFT20	unnamed (-90)	1.099604413	0.8665988
MYO1D	unnamed (+54243)	#N/A	0.868111351
SSR1	unnamed (-51)	0.900400128	0.868174741
H2AFV	unnamed (-376), unnamed (+487)	0.657525278	0.868225154
ATE1	unnamed (-90)	1.082937084	0.868231516
ZNF639	unnamed (-714)	0.63855001	0.868240444
PEBP1	unnamed (+18)	1.048557359	0.868723813
PFKM	unnamed (+416)	0.78112578	0.869009249
NARG2	unnamed (+181)	0.824331327	0.869089004
PPP1R10	unnamed (+1025)	1.017370579	0.869229229
KIAA1191	unnamed (+275)	1.208702009	0.869889838
MRPL19	unnamed (+69)	0.765582764	0.869907406
ANKIB1	unnamed (-295)	1.061624491	0.869991571
CRLF3	unnamed (+164)	1.202746855	0.870648565
VAMP1	unnamed (-122)	0.763976281	0.870734145
JRK	unnamed (-15)	0.835422771	0.871237333
PTPMT1	unnamed (-12088), unnamed (+130)	0.83477478	0.871345525
SLC18A2	unnamed (-24309)	#N/A	0.87139712
NGDN	unnamed (-225)	1.145947711	0.871589678
SMC1A	unnamed (+315)	1.225432271	0.871733165
NFATC2IP	unnamed (-251)	0.853086082	0.872013352
CCT5	unnamed (-233)	0.926196375	0.872018008
ARL3	unnamed (+575)	0.79414663	0.872025439
PIP4K2B	unnamed (-25594), unnamed (-102)	0.825640827	0.87212687
TFG	unnamed (-195)	1.179090358	0.872200562
MYBPC2	unnamed (-1162)	#N/A	0.872371743
APPBP2	unnamed (+12)	1.052510641	0.87248333
ZNF652	unnamed (+369)	1.421674572	0.872712337
BANF1	unnamed (+162)	0.986035403	0.873102284
SREK1IP1	unnamed (-339)	0.896473963	0.873215613
TUBGCP3	unnamed (-18362)	0.832997824	0.873624792
RBM22	unnamed (+8)	1.095618656	0.874008056
SBNO1	unnamed (-15076)	0.689952097	0.874059685
DDHD2	unnamed (-397)	0.876776453	0.874103432
ZKSCAN5	unnamed (-4416), unnamed (-39), unnamed (+530)	0.716664675	0.875095803
ITGB1BP1	unnamed (-119)	0.842523119	0.875635188

TRMT5	unnamed (+20)	0.715858875	0.875960688
INPP5A	unnamed (-496)	1.143528357	0.876818653
SPSB2	unnamed (-17890), unnamed (+94)	0.984152624	0.877516847
HIST1H1C	unnamed (+1153)	0.334632591	0.877816395
SLC6A19	unnamed (-27072)	#N/A	0.878105918
TMEM143	unnamed (-368), unnamed (+647)	#N/A	0.878651798
SIRT5	unnamed (-446)	0.788065563	0.87871635
PSMA3	unnamed (+270)	0.977041251	0.878788728
HSPA4L	unnamed (+267)	0.821649914	0.878863081
UBE2R2	unnamed (-198), unnamed (+232100)	1.37341177	0.879213465
PDGFB	unnamed (-66138)	#N/A	0.879361561
NCBP1	unnamed (-221)	1.134076416	0.879636283
PPP2R5C	unnamed (+186443)	0.581219958	0.879750929
TMEM146	unnamed (-221)	#N/A	0.8800372
TSPYL1	unnamed (+152)	0.775495819	0.880102462
EXTL3	unnamed (-79344)	#N/A	0.880544596
PHACTR2	unnamed (+165146)	0.770543517	0.881010032
CAMKMT	unnamed (-85)	#N/A	0.881393534
TAOK1	unnamed (-644)	#N/A	0.881690369
CEP63	unnamed (-138)	0.972959278	0.881998318
DDX20	unnamed (+141)	0.954971405	0.882220874
BUB1B	unnamed (+176)	1.282035011	0.882314332
KAZALD1	unnamed (-18174), unnamed (-673), unnamed (+6800)	0.909831537	0.882562593
MVK	unnamed (-311)	0.977690051	0.882868162
GRID1	unnamed (-10916)	#N/A	0.88299414
BAHCC1	unnamed (+77920)	1.432014393	0.883372093
COMMD8	unnamed (-60)	0.599420363	0.884000058
SHISA5	unnamed (+469)	1.1917332	0.884164019
BYSL	unnamed (-10)	1.015204051	0.884331375
KLF6	unnamed (-203)	1.199829404	0.885012118
RBM15B	unnamed (-103)	0.982746356	0.885904399
PRDM10	unnamed (+176)	0.983282065	0.885947971
EGR1	unnamed (-633)	#N/A	0.88629925
NUP50	unnamed (-53)	0.795729784	0.886479843
DRG2	unnamed (-70)	0.953348847	0.886603631
R3HCC1	unnamed (-41508)	1.110560195	0.886656383
RPL24	unnamed (-8)	0.906295244	0.887642719
CCBL1	unnamed (+29)	0.93077435	0.888022779
ANKAR	unnamed (-1361)	#N/A	0.888059832
LRRC23	unnamed (-13558)	#N/A	0.888067095
YY1	unnamed (-45625), unnamed (+31)	0.68535214	0.888216619
LHFPL4	unnamed (+157252)	#N/A	0.888246918
EIF2A	unnamed (-175)	0.886905632	0.889123727
NMUR1	unnamed (-82185)	#N/A	0.889275388
PUF60	unnamed (+138)	1.06508027	0.889674698
NAT10	unnamed (-194), unnamed (+69003)	0.711020531	0.890017591
CCDC90B	unnamed (+197)	1.099111014	0.890088321
TXLNG	unnamed (-125)	0.930658897	0.890442632
MCM3AP	unnamed (-1353)	1.049274734	0.890457075
C15orf44	unnamed (-13)	1.290416256	0.891768098

DDX41	unnamed (-186)	0.693174292	0.891889921
SSB	unnamed (+184)	0.918464569	0.892427242
CAB39	unnamed (-192)	1.142919996	0.89277771
PRDM14	unnamed (-330987)	#N/A	0.892932145
BRIP1	unnamed (+12)	1.132455079	0.89319974
MBLAC2	unnamed (+396)	0.47713516	0.893455817
PCBP1	unnamed (-887), unnamed (-217), unnamed (+55230)	1.002368562	0.894326571
MMS22L	unnamed (-123)	#N/A	0.894394655
DAND5	unnamed (-4106)	#N/A	0.8950759
RPL12	unnamed (-77)	0.89023784	0.895373498
EEF1E1	unnamed (0), unnamed (+38062)	0.880061756	0.895487338
PGP	unnamed (-443)	0.896798838	0.89610629
MYLK2	unnamed (+3626)	#N/A	0.896751474
ZNF589	unnamed (+164)	#N/A	0.897085717
C3orf78	unnamed (+177)	1.017069437	0.897256427
SELPLG	unnamed (+27892)	#N/A	0.897592194
ZC3H3	unnamed (-74)	#N/A	0.898296035
EXOSC3	unnamed (+41)	0.686079061	0.898514425
CDKL3	unnamed (+141067)	#N/A	0.898604332
PHF17	unnamed (+1101)	0.530444946	0.89885518
DDX42	unnamed (-528)	0.716091802	0.89940909
CAPN7	unnamed (-161)	0.808966968	0.899843901
THAP5	unnamed (+32)	0.804144694	0.899992579
SEPT7	unnamed (-244)	0.996762215	0.900843395
ABI3	unnamed (-17820)	#N/A	0.901447838
PRDM4	unnamed (-207)	1.396798278	0.901773188
PANK3	unnamed (-105)	0.577083038	0.902078092
CCNI	unnamed (-329)	1.502995373	0.90208406
TP53	unnamed (+273)	1.043846758	0.902135109
FAM96B	unnamed (-30)	1.027258761	0.902354438
HMBOX1	unnamed (-175)	0.716082577	0.902479382
FKBP2	unnamed (+14)	0.954709458	0.902955516
AAGAB	unnamed (-111)	1.855650336	0.903072512
UFSP2	unnamed (-107)	0.801803178	0.904679954
C14orf93	unnamed (+3341)	#N/A	0.904886468
PSIP1	unnamed (-42124), unnamed (-214)	0.82328265	0.904995129
PRICKLE4	unnamed (-711)	#N/A	0.905143952
SLC41A3	unnamed (-28)	0.862500412	0.905227313
CUTA	unnamed (-139)	1.178387465	0.905748041
EIF3B	unnamed (-349)	0.861209276	0.906005064
DDB1	unnamed (-328)	0.929310935	0.906090798
PAIP2	unnamed (-38)	0.738647434	0.906305347
FAM108B1	unnamed (-271)	1.666524674	0.906441307
PIGP	unnamed (-329)	0.763622297	0.90662118
KIAA1524	unnamed (-98)	1.044074067	0.906895993
CTDNEP1	unnamed (-611)	1.259666972	0.906943656
ACD	unnamed (-282)	1.006232745	0.907197085
SRPR	unnamed (-84)	0.737117554	0.908103694
ZKSCAN2	unnamed (-441)	#N/A	0.908461936
CDC5L	unnamed (-20)	0.867374143	0.908888761

TSPAN31	unnamed (+7)	0.763011667	0.909566008
ECT2L	unnamed (-22587)	#N/A	0.909753839
ADPGK	unnamed (-284)	1.085763073	0.910181151
TGS1	unnamed (+224)	0.974839444	0.91047323
RPP38	unnamed (-32)	1.188836507	0.911233483
ZNF12	unnamed (-58)	1.26510337	0.911345193
MOG	unnamed (-6720)	#N/A	0.911857364
EFTUD2	unnamed (+95)	0.998772013	0.912283449
CDK2AP2	unnamed (+7)	1.450130458	0.912372344
WASL	unnamed (-45)	1.05238205	0.913039756
SETD1B	unnamed (-1768)	1.194163398	0.913064717
FANCG	unnamed (+7)	0.794265997	0.913282388
OMA1	unnamed (-68)	1.192223876	0.913296845
CLDND2	unnamed (+2416)	#N/A	0.913322056
TKTL2	unnamed (-20649)	#N/A	0.913660722
SYNGAP1	unnamed (-1643)	#N/A	0.914448887
EPN1	unnamed (-1416)	#N/A	0.914620922
SSC5D	unnamed (+15502)	#N/A	0.914917886
RCE1	unnamed (+27)	0.923638111	0.915295197
YBEY	unnamed (+322)	#N/A	0.91555737
GSG2	unnamed (-200)	0.741961079	0.915569132
TGOLN2	unnamed (+237)	0.872351477	0.915746251
STK16	unnamed (+30)	#N/A	0.915823884
CNOT6	unnamed (-168)	1.042965044	0.915936824
ATP5G1	unnamed (-240)	0.798113833	0.916048125
ATXN2L	unnamed (-625), unnamed (+854)	#N/A	0.916129981
RNASEH1	unnamed (-154)	0.76166988	0.916178069
CKS2	unnamed (-229)	0.980443733	0.916231187
RNF38	unnamed (-87657)	0.736322336	0.916397982
GABBR1	unnamed (-17076)	#N/A	0.917150437
SSH2	unnamed (+168450)	0.507583368	0.917717582
OSR1	unnamed (+10168)	#N/A	0.918239801
NUPL2	unnamed (+1)	0.86837916	0.918571203
IKZF5	unnamed (+63)	0.703962457	0.918571617
C14orf1	unnamed (+52)	0.722630666	0.919155205
MRE11A	unnamed (+39)	0.760565197	0.919272538
ATP5D	unnamed (-1379)	0.683026166	0.920319052
FAM98B	unnamed (-59)	0.719276594	0.920465785
PTRH2	unnamed (+15)	0.95448698	0.920589141
UQCR10	unnamed (-266)	1.424388245	0.920703898
MPP5	unnamed (-150)	1.495921158	0.920820893
EIF2AK3	unnamed (-353)	2.153553436	0.920932641
TAF12	unnamed (-229)	0.834221867	0.92136212
SGMS1	unnamed (-656)	0.923732394	0.92204215
MAP3K7	unnamed (-114)	0.882395428	0.922111605
LBP	unnamed (+89138)	#N/A	0.922299385
SMARCC1	unnamed (-10)	0.642959321	0.922503303
TEX14	unnamed (-646)	#N/A	0.923960451
ZNF579	unnamed (-6576)	#N/A	0.924453747
OVOL1	unnamed (+31354)	#N/A	0.924588161
ZBTB49	unnamed (+45220)	#N/A	0.924624977

DDX55	unnamed (-114)	0.914066371	0.92492031
ARL10	unnamed (-3968)	#N/A	0.92555833
SCAND3	unnamed (-308977)	1.229578248	0.926004274
HNRPDL	unnamed (-251), unnamed (+631)	0.707334358	0.926095989
MAP3K9	unnamed (-243)	1.802261537	0.926248545
NDUFA6	unnamed (-112)	0.925646253	0.926589911
TSKS	unnamed (-2642)	#N/A	0.927012679
NPTX1	unnamed (+21947)	1.270197872	0.927316637
ATF2	unnamed (+201)	0.713931956	0.927429456
B4GALNT4	unnamed (-13615)	#N/A	0.927452922
PTPN1	unnamed (-194)	0.953493941	0.927530297
H2AFZ	unnamed (-74)	0.909679312	0.927565343
ZNF317	unnamed (+85)	1.267597032	0.927724104
ZNF317	unnamed (+85)	1.267597032	0.927724104
NBN	unnamed (+98)	0.899662757	0.927836579
SNRPA	unnamed (-28)	0.838450679	0.928019677
EIF4EBP2	unnamed (-39426)	0.58110068	0.928203007
SFXN3	unnamed (+11829)	0.898999076	0.928638222
DLST	unnamed (-51)	0.958358992	0.928720176
YOD1	unnamed (-1883), unnamed (+481)	1.385785612	0.928977596
C7orf10	unnamed (-248)	#N/A	0.929303241
ZFHX2	unnamed (-4626), unnamed (-169)	#N/A	0.929573169
COPG	unnamed (+28953)	1.015715946	0.929732985
TRUB1	unnamed (+1)	1.088588901	0.930128451
NCOA2	unnamed (+1471)	1.271628271	0.930148953
CNNM4	unnamed (-268)	#N/A	0.930348697
PSMC3IP	unnamed (+12)	1.011441222	0.93125345
TTI1	unnamed (-96)	1.399212544	0.931831979
MYLK3	unnamed (-41739)	0.423630988	0.931963877
PTPN11	unnamed (+152)	0.656776877	0.932085223
SLC30A5	unnamed (-34)	0.699776148	0.932335785
SRSF1	unnamed (+63)	0.548605944	0.93268316
DNAJA3	unnamed (-117)	0.920949199	0.932688897
NKX6-3	unnamed (-181380)	#N/A	0.933095973
RNMTL1	unnamed (+78)	1.175486676	0.933149988
NSFL1C	unnamed (+701)	0.83571786	0.93327402
RBM12B	unnamed (+81)	0.902829188	0.933355131
PLDN	unnamed (+80)	0.564373024	0.933451493
C14orf135	unnamed (+50)	#N/A	0.933815389
TXNRD3	unnamed (-327527)	#N/A	0.933859163
ATP5H	unnamed (+12875)	0.900807096	0.933952998
VTI1B	unnamed (-25)	0.794106751	0.934124732
MAPKAPK5	unnamed (-623)	0.836716132	0.934140158
PTTG1	unnamed (-2568)	0.81888049	0.934176396
CCT7	unnamed (-795)	0.903819411	0.934238947
PRKCI	unnamed (-254)	1.212592273	0.934570479
AHCYL1	unnamed (-108)	0.960317735	0.934636764
UMPS	unnamed (-37)	0.855147422	0.934940219
SLC7A11	unnamed (+38)	7.602955925	0.936005838
TCF12	unnamed (-39)	0.617876439	0.936493446
FOXK2	unnamed (-347)	0.988574957	0.937376379

EXOSC7	unnamed (+1)	0.969827389	0.937574309
CCDC142	unnamed (-46)	#N/A	0.938898943
ZNF367	unnamed (+951)	1.045286709	0.939233287
SIAE	unnamed (+24)	1.257212094	0.939238516
AGA	unnamed (+15)	1.056389522	0.939322749
ZNF192	unnamed (-5044)	1.096720797	0.939401059
LUC7L2	unnamed (-120), unnamed (+18408)	0.578940188	0.939748418
STX11	unnamed (+65821)	#N/A	0.940273813
NPM2	unnamed (-14535)	#N/A	0.940422627
EED	unnamed (+76)	1.28996325	0.940444651
MTERF	unnamed (-126)	#N/A	0.941103034
DCAF6	unnamed (-86)	0.683240479	0.941171878
ARHGEF4	unnamed (-117011)	0.776293565	0.941696925
NMT1	unnamed (-114)	0.758462259	0.942201492
AIM1L	unnamed (+32434)	#N/A	0.942431309
GPANK1	unnamed (-151)	#N/A	0.942479582
MIEN1	unnamed (-39)	#N/A	0.942637047
IFT122	unnamed (+42)	0.749758915	0.942969193
MRPS27	unnamed (-13), unnamed (+12056)	0.947491459	0.94369724
TRIM69	unnamed (-7477)	#N/A	0.944086569
GCFC1	unnamed (+44)	0.89087239	0.944431618
SPAG4	unnamed (-57)	#N/A	0.944788951
ATP8B4	unnamed (+498381)	#N/A	0.94481895
NUP210L	unnamed (-28235)	#N/A	0.945022431
SNX32	unnamed (-15551)	#N/A	0.945938466
EFHC2	unnamed (-199384)	0.863232248	0.946000914
DCLRE1B	unnamed (+13)	1.287313211	0.946760047
XRN2	unnamed (+303)	0.842373656	0.946985965
BCCIP	unnamed (-41)	0.691044738	0.947271025
SPAG16	unnamed (+20)	1.512509028	0.947371575
ULK4	unnamed (-50)	#N/A	0.947803849
RBAK	unnamed (-21)	1.252979572	0.949280656
ZNF335	unnamed (+40)	#N/A	0.949387789
CITED2	unnamed (-1374), unnamed (-459)	1.470045763	0.949560653
KCNK18	unnamed (+19275)	#N/A	0.94964337
ABHD11	unnamed (-96)	0.953611777	0.949870525
KDM4C	unnamed (-76184)	#N/A	0.950029333
CLTA	unnamed (+67693)	1.127325979	0.950717418
EXTL2	unnamed (-1305)	0.870778506	0.95075017
BHLHE40	unnamed (+1062)	1.073067158	0.950802306
RPS8	unnamed (-187)	0.947403306	0.952195565
USP37	unnamed (-436)	1.234758125	0.952241587
CCDC77	unnamed (-60)	1.278959099	0.952931167
R3HDM1	unnamed (-636)	0.505706422	0.953155624
MTHFSD	unnamed (-19)	0.611345262	0.953191201
HRSP12	unnamed (-165)	0.646621951	0.95344862
NEK1	unnamed (+188)	0.711527759	0.953874833
RBM7	unnamed (+78)	0.760545386	0.954770955
IRS4	unnamed (-318013)	#N/A	0.954988328
STRAP	unnamed (+109)	1.024563679	0.955141183
CCT8	unnamed (-149)	1.070053947	0.955142016

PHRF1	unnamed (-150)	#N/A	0.955866537
FOXS1	unnamed (+22616)	#N/A	0.956115025
AASDH	unnamed (+144)	1.101353986	0.956507524
EFHC1	unnamed (+54)	0.957557883	0.956533312
GAP43	unnamed (-476630), unnamed (+161381)	#N/A	0.956850136
NUP85	unnamed (+33)	0.958644645	0.957241292
ECHDC1	unnamed (-905)	0.854074082	0.957326527
TDP1	unnamed (+31)	0.719130668	0.95736636
OSBP	unnamed (+161)	1.309103497	0.957390563
SPEF1	unnamed (+13129)	#N/A	0.957704957
PDCL3	unnamed (+301)	#N/A	0.957731447
SRSF6	unnamed (-97)	1.158322084	0.95780915
SAV1	unnamed (-166)	1.152360735	0.957935091
C11orf30	unnamed (-255)	1.030145709	0.958160091
ZNF622	unnamed (-105)	1.398842667	0.958181385
TMED5	unnamed (+623)	0.883052868	0.958306072
TSC22D1	unnamed (-341813), unnamed (+141027)	0.836577708	0.958452252
C16orf7	unnamed (-1277), unnamed (+20701)	#N/A	0.958615519
KRBA1	unnamed (-90201)	#N/A	0.958785294
METTL20	unnamed (+69450)	#N/A	0.959288358
ASXL1	unnamed (-324)	0.531606407	0.96011251
ZCCHC6	unnamed (+96)	1.069821334	0.960611469
ABCB10	unnamed (-352)	1.259939207	0.960730136
PIGG	unnamed (+9)	1.187421435	0.960783785
CD81	unnamed (+23142)	0.994945828	0.961170124
C1orf124	unnamed (-408), unnamed (+300)	1.024269425	0.961269052
KRAS	unnamed (-135332)	1.011248012	0.961624482
RFFL	unnamed (-56127)	1.664749077	0.961773669
DNTTIP1	unnamed (+17)	0.989868047	0.962163194
SYT3	unnamed (-39789)	#N/A	0.962230301
MRPS24	unnamed (-71)	0.865182125	0.962525936
ZNF646	unnamed (+21)	#N/A	0.964567341
THADA	unnamed (+20)	0.995703552	0.964716064
KIAA0141	unnamed (+114)	0.726317148	0.964806004
TEX10	unnamed (-66)	1.045418606	0.965067997
ZNF3	unnamed (-70)	#N/A	0.965543292
NCBP2	unnamed (+81)	1.061190889	0.966138255
C17orf90	unnamed (-220)	0.855928924	0.966615895
RRNAD1	unnamed (+190)	#N/A	0.966971312
FUBP1	unnamed (-147)	0.524002218	0.967083456
SEC23B	unnamed (+56)	1.183904467	0.9671192
PMS2	unnamed (+202)	#N/A	0.968589605
HECTD1	unnamed (-98)	0.969201996	0.968597002
NANOS3	unnamed (-30130), unnamed (+3046)	#N/A	0.968643374
LMOD1	unnamed (+50484)	#N/A	0.968933131
KIAA1468	unnamed (-266)	1.229028319	0.969415567
SETDB2	unnamed (-7591)	#N/A	0.969930373
VPS37B	unnamed (-102)	1.207804801	0.970193582
YIF1A	unnamed (+6508)	1.154062667	0.970250843
HBQ1	unnamed (+1095)	#N/A	0.970589346
ELMOD2	unnamed (+170)	0.844641822	0.971130861

G3BP1	unnamed (-313)	0.876504624	0.971193901
PTCRA	unnamed (-25226)	#N/A	0.971419722
FLCN	unnamed (-257)	2.666433553	0.972119795
ETFDH	unnamed (-227)	1.076827288	0.972217363
TFAP4	unnamed (+18842)	#N/A	0.972834695
COL1A1	unnamed (+891)	0.811674315	0.972947598
SUV420H2	unnamed (-832), unnamed (-149)	#N/A	0.97319804
YWHAZ	unnamed (+835)	0.992634048	0.974188025
TTC21A	unnamed (-30)	#N/A	0.974221572
TP53BP2	unnamed (-146673)	0.658758542	0.974257508
NANP	unnamed (-309)	0.682145637	0.974507184
JUND	unnamed (-123)	1.145988908	0.974705975
TCF7	unnamed (-12543)	0.855624652	0.975374221
ARPM1	unnamed (+4746)	#N/A	0.975600851
C14orf102	unnamed (-51062)	0.871778986	0.97562535
THAP1	unnamed (+151)	0.884731929	0.976115262
TMEM203	unnamed (-36)	0.787963787	0.9762663
PMS1	unnamed (-24)	1.036040215	0.976429362
FABP2	unnamed (-132545)	#N/A	0.976552013
FBXW7	unnamed (-1308)	0.853746951	0.976632002
DOM3Z	unnamed (-88)	0.683710734	0.976796355
VPS37A	unnamed (-15)	0.927060212	0.977260129
C10orf111	unnamed (+168)	#N/A	0.977396872
MOB1A	unnamed (-59)	#N/A	0.978199637
DUSP21	unnamed (-300942)	#N/A	0.978232948
DCAF12	unnamed (+77489)	1.215146699	0.978369793
HIST4H4	unnamed (+750)	#N/A	0.979096896
ZNF500	unnamed (-1567)	1.092789914	0.979278281
PIGB	unnamed (-28294)	0.699193578	0.979668016
ANXA7	unnamed (+126)	0.811083278	0.979870181
SLC39A9	unnamed (-170)	0.797979143	0.980020496
CLCN3	unnamed (-276)	1.027591231	0.980154364
COX4I2	unnamed (-24898)	#N/A	0.980220167
RFC1	unnamed (+114)	0.526957791	0.980387067
SLC10A2	unnamed (+221056)	#N/A	0.980573775
TINF2	unnamed (-190)	1.046099446	0.981210182
CISD2	unnamed (+125)	0.711412038	0.981228759
LCMT1	unnamed (-4490)	0.986489165	0.981358811
NOC4L	unnamed (-35), unnamed (+9615)	0.986380209	0.981483566
EXOC6B	unnamed (-36508)	1.112461085	0.981751805
MORF4L1	unnamed (+1022)	0.892634301	0.981874608
VPS13C	unnamed (-82)	1.444424477	0.982192745
SP4	unnamed (-469)	0.653683154	0.98275077
RPN2	unnamed (+103)	0.469385412	0.983278905
POLG	unnamed (-361)	0.844655657	0.983317438
HSD17B13	unnamed (+102263), unnamed (+315795)	#N/A	0.983644328
TCF3	unnamed (-2689)	0.70517836	0.984264773
CHST8	unnamed (+156227)	#N/A	0.984632677
PWP1	unnamed (-73)	1.180953227	0.984678239
ULBP3	unnamed (+121)	#N/A	0.984759559
ZFC3H1	unnamed (-55)	0.777722574	0.984967051

DIABLO	unnamed (+1449)	1.16075103	0.985024839
ZNF337	unnamed (-512194)	1.236372049	0.985107653
ZDHHC5	unnamed (-297)	1.05238253	0.985226175
PHB2	unnamed (-40)	1.010230825	0.985490964
RPN1	unnamed (-30084)	1.065278174	0.985820363
SCAMP1	unnamed (+60)	0.595874584	0.986054576
ZNF764	unnamed (-315)	1.099379604	0.986128772
RBM4B	unnamed (-372)	1.026059584	0.986181966
CPN2	unnamed (+38629)	#N/A	0.986433736
ITGA1	unnamed (+12396)	0.798283419	0.986757138
PAX2	unnamed (+167667)	#N/A	0.987335069
PLEKHG2	unnamed (-648)	#N/A	0.987525468
TMEM41A	unnamed (+49)	1.35314968	0.987886029
ZBTB37	unnamed (-443)	#N/A	0.987951781
COX5B	unnamed (+136)	1.015835464	0.988237843
GRK4	unnamed (-430)	#N/A	0.988252975
ETV5	unnamed (+565)	0.939711857	0.989381385
SRRM2	unnamed (+74)	0.920724695	0.98949612
NBPF1	unnamed (-30980), unnamed (-797)	#N/A	0.98965637
IFLTD1	unnamed (+167031)	#N/A	0.989734938
DCAF16	unnamed (-145)	0.882896548	0.989931746
CEP152	unnamed (+153)	0.77828621	0.990099837
NAA38	unnamed (+130)	0.808440167	0.990325948
GOT2	unnamed (-52)	1.168209968	0.990392148
MTF2	unnamed (+67)	0.860333217	0.990998885
GEMIN4	unnamed (-105)	1.161832877	0.991413795
ARG2	unnamed (-30)	#N/A	0.99142077
PURG	unnamed (-914)	#N/A	0.991703247
GGA1	unnamed (+261)	0.99817262	0.992096484
STK35	unnamed (+548)	1.423755046	0.992471172
RPLP0	unnamed (-27591), unnamed (-51)	0.989465944	0.992542093
SCARF1	unnamed (-3157)	#N/A	0.99254381
CDH17	unnamed (+217049)	#N/A	0.992565355
SRGAP1	unnamed (+377824)	1.020035162	0.992817779
FRAT1	unnamed (-221)	0.902854191	0.993668124
TMEM209	unnamed (+34)	1.467231671	0.993804886
GRIP2	unnamed (-109525)	#N/A	0.994019597
BMS1	unnamed (+227)	0.824611576	0.994162822
CIC	unnamed (-16259)	#N/A	0.994848454
CDC123	unnamed (-9)	0.868430637	0.995282966
SNAP29	unnamed (-71)	0.568242122	0.9955907
TP53I13	unnamed (+140)	#N/A	0.995698145
AAAS	unnamed (-96)	1.190989038	0.996771612
HTRA3	unnamed (+158735)	#N/A	0.996881862
SLC25A36	unnamed (-76)	0.979033667	0.997183521
CRYZL1	unnamed (-269)	0.652118014	0.997611783
C7orf53	unnamed (-30226)	#N/A	0.997663723
ZNF571	unnamed (+135)	#N/A	0.997728146
ZNF239	unnamed (+118841)	0.790033409	0.998545079
SIPA1L1	unnamed (-209086)	#N/A	0.998657814
OLFML2A	unnamed (+75911)	#N/A	0.998678859

MAPK8IP3	unnamed (-9)	#N/A	0.9988234
C19orf26	unnamed (-2380)	#N/A	0.999365392
NARS	unnamed (+102)	1.079671003	0.999816496
PEX11B	unnamed (+172)	1.026115048	1.000040288
ACTR3	unnamed (-247)	0.924243554	1.000319771
LSM14B	unnamed (-306)	#N/A	1.000380623
CREM	unnamed (-409)	0.882747082	1.000419775
PIGO	unnamed (+65)	1.096012455	1.000739419
ZC3H4	unnamed (+76)	1.091228546	1.000870655
RLF	unnamed (-151)	1.117414981	1.001144268
KDM6B	unnamed (-4224)	1.313709705	1.0013367
METTL16	unnamed (-28)	#N/A	1.001389168
CHAF1A	unnamed (-141)	0.863008793	1.002585808
CDADC1	unnamed (-72)	0.662816114	1.002765101
DCAF8	unnamed (+26)	0.93928755	1.003818272
C2orf28	unnamed (+236)	0.893825794	1.004055181
PI4KA	unnamed (-121)	1.053938502	1.004113206
NOP56	unnamed (+26)	0.578073229	1.004193672
SEPT1	unnamed (-17702)	#N/A	1.004677015
MRPL13	unnamed (+87)	0.798594747	1.004848576
USP53	unnamed (-346)	1.16796768	1.004955892
GGNBP2	unnamed (-328)	1.041882124	1.005436589
C6orf62	unnamed (-1958)	0.80959051	1.005564455
OIP5	unnamed (+272)	0.772378697	1.005753533
CDK18	unnamed (-18860)	#N/A	1.005781976
LMBRD2	unnamed (-426)	1.127375032	1.006356289
TATDN1	unnamed (+158)	0.962111083	1.006469793
KIF22	unnamed (0)	0.838902919	1.006554264
C11orf1	unnamed (-71)	1.104137868	1.006711925
CNNM3	unnamed (+69)	1.030318057	1.006896964
MRM1	unnamed (-340)	#N/A	1.006977794
NHP2	unnamed (+49)	0.380577542	1.007309064
NUS1	unnamed (-285)	0.798207519	1.007374531
RAB5B	unnamed (-177)	1.416787262	1.007551841
HDAC6	unnamed (-479)	1.569740249	1.007925583
TRA2B	unnamed (+447)	0.812616541	1.008065728
GSS	unnamed (-21)	0.914138464	1.008294092
C11orf82	unnamed (-99)	0.773376263	1.008611479
PDS5A	unnamed (-98)	0.781151183	1.008645199
NXPH3	unnamed (-7869), unnamed (-5651)	#N/A	1.008836132
SSBP1	unnamed (+28)	0.896485736	1.008868915
SLA2	unnamed (+40648)	#N/A	1.008919311
PIK3CD	unnamed (-24577)	#N/A	1.008976312
ZNF425	unnamed (-107)	#N/A	1.009215944
MYOZ1	unnamed (+15639)	#N/A	1.009387677
MIA3	unnamed (+26163)	0.881164266	1.009409315
HSPA14	unnamed (-74)	0.572957495	1.009460456
HSPA14	unnamed (-74)	0.572957495	1.009460456
TMPRSS11B	unnamed (-104470)	#N/A	1.009732226
ATP5I	unnamed (-46)	0.864541152	1.009817125
FAM206A	unnamed (-288)	#N/A	1.010118046

MEIS2	unnamed (+521171)	0.771655928	1.010233931
SCGB1C1	unnamed (-3035)	#N/A	1.010454211
EXOSC10	unnamed (+130)	0.792003263	1.010471954
DDX19B	unnamed (+47652)	#N/A	1.010649838
ANAPC13	unnamed (-46)	1.179926759	1.010949479
ADAMTS16	unnamed (+282063)	#N/A	1.011026853
NDUFB6	unnamed (-97)	0.970527027	1.011049156
TAF11	unnamed (-35)	0.86133573	1.011058717
SS18	unnamed (-244)	1.034327922	1.011776318
SLC26A7	unnamed (-179127)	#N/A	1.011963808
YTHDC2	unnamed (-63)	0.904107996	1.012525845
GNAS	unnamed (+35953)	0.603924454	1.012617885
SLC35G5	unnamed (+135890)	#N/A	1.012722631
RNF8	unnamed (+39)	1.036891222	1.012753364
RNF151	unnamed (-1979)	#N/A	1.013041981
SUV39H2	unnamed (-101)	0.675508782	1.013147612
ACO2	unnamed (-170)	1.24618258	1.013542948
CHD1L	unnamed (-55)	0.743244058	1.013551202
CEP135	unnamed (-209)	#N/A	1.013629367
ZNF346	unnamed (+42)	1.155295238	1.013702905
LAMP1	unnamed (-9)	1.12745838	1.013825821
OSBPL8	unnamed (+77)	0.669426896	1.015011251
DCAF17	unnamed (+507)	0.835655156	1.015127056
RABGEF1	unnamed (-85515), unnamed (+104808)	#N/A	1.015205492
ARL4D	unnamed (-37757), unnamed (-29771), unnamed (-10027), unnamed (-1)	0.732748005	1.015914716
HYAL1	unnamed (-18048)	1.230667841	1.01625315
TACO1	unnamed (-21)	1.12477136	1.016317053
MFSD11	unnamed (+55)	0.857960619	1.01633722
GZF1	unnamed (-2398)	1.444580581	1.016397722
BAP1	unnamed (-41)	0.930496202	1.016553114
CREB3L1	unnamed (-34237)	#N/A	1.01663159
KCMF1	unnamed (-45107)	1.117311286	1.016691407
SCARB1	unnamed (-46787), unnamed (+96777)	0.965146342	1.017863549
RAB11A	unnamed (-194)	1.027147118	1.017975645
TMEM68	unnamed (-130)	0.630858215	1.018035522
DNAJC27	unnamed (+116)	#N/A	1.018078323
WNK1	unnamed (-1658)	0.94275073	1.018317616
WDPCP	unnamed (-4)	#N/A	1.018539244
TTC31	unnamed (+203)	1.072158924	1.018736577
PTCHD2	unnamed (-138)	#N/A	1.018985771
PHF12	unnamed (-737)	#N/A	1.019124724
RHOD	unnamed (+24835)	0.530654488	1.019668168
GDF7	unnamed (+156356)	#N/A	1.01972329
MAT2A	unnamed (-472)	0.804586422	1.01972405
SENP1	unnamed (-431)	0.660946175	1.02016495
IPO4	unnamed (+842)	0.530585103	1.021388678
MED10	unnamed (+956133)	1.764681375	1.021515575
PANK2	unnamed (+231)	0.978292678	1.021664244
CLN5	unnamed (+190)	1.120879212	1.021671237
C9orf5	unnamed (-203)	1.424351603	1.021996591

TLCD1	unnamed (-568)	0.813059034	1.022027268
SART3	unnamed (-1094)	1.149161026	1.023356107
EIF2AK2	unnamed (-40)	1.429404656	1.023604162
DDX3X	unnamed (+472)	1.322226799	1.023695272
PNPLA8	unnamed (-153)	0.714627947	1.024241286
PGRMC2	unnamed (+633)	1.128656828	1.024770551
EFNA3	unnamed (+6024)	#N/A	1.024793176
SENP2	unnamed (-115)	0.656773682	1.02505791
CD52	unnamed (+3776)	#N/A	1.025964024
C13orf16	unnamed (-167101)	#N/A	1.026024818
NPHP4	unnamed (+315)	#N/A	1.026253251
EIF2C3	unnamed (+33)	0.748995262	1.026844822
ASAH1	unnamed (-23)	1.048529161	1.027034777
SLC25A37	unnamed (-53)	0.862270301	1.027129407
XRN1	unnamed (-146)	0.980927411	1.027130419
TMEM127	unnamed (-27)	1.085609635	1.027206595
UBR4	unnamed (-79)	0.917649114	1.027494366
HMGB3	unnamed (-9)	0.5771203	1.027716689
C19orf77	unnamed (+45443)	#N/A	1.027782773
UNG	unnamed (-3987)	0.992410401	1.027815972
ZCCHC14	unnamed (-1183)	0.885971993	1.028601961
DQX1	unnamed (-3548)	#N/A	1.028732687
MLF2	unnamed (-913)	1.2830095	1.028877346
RBM45	unnamed (-78)	1.10285825	1.029236494
USP54	unnamed (-50443)	0.680444863	1.030966611
UBE2N	unnamed (+142)	0.926510806	1.031125355
GTF2I	unnamed (-391)	0.911924676	1.031163653
LAMP2	unnamed (-91985)	0.68076154	1.031203735
C7orf42	unnamed (-76412)	0.96272204	1.031250622
SIGLEC10	unnamed (+23387)	#N/A	1.031670443
SGK196	unnamed (+220)	#N/A	1.032237555
APTX	unnamed (+4)	0.949306163	1.032420061
POLDIP3	unnamed (-359)	1.190894834	1.032616193
PSRC1	unnamed (+19367)	1.062717264	1.032688319
WFDC3	unnamed (-46)	#N/A	1.033029421
MLC1	unnamed (+54936)	#N/A	1.033591999
ATP8A1	unnamed (-289)	#N/A	1.034205086
ARID4B	unnamed (-428)	1.264703573	1.035031315
FXC1	unnamed (+41)	0.855887137	1.03506982
KIAA1967	unnamed (-433)	1.017470371	1.035820836
STK11IP	unnamed (+409)	#N/A	1.035829787
NBEAL2	unnamed (-3106)	#N/A	1.036934835
CNTROB	unnamed (+47)	#N/A	1.037011413
UBXN4	unnamed (-47)	0.931226881	1.037204915
NACA	unnamed (-892)	0.678965263	1.037662137
GPR56	unnamed (+29220)	2.112999138	1.038546752
SERBP1	unnamed (-243)	0.751274842	1.038577049
MMAB	unnamed (+169)	0.557151945	1.038844188
GSC2	unnamed (-21333)	#N/A	1.039088873
TMEM106A	unnamed (+74702), unnamed (+82688), unnamed (+102432)	#N/A	1.039803875

GTPBP10	unnamed (-60)	1.036150506	1.039859625
NDUFA7	unnamed (+140)	1.054128504	1.039952152
FDPS	unnamed (+47)	0.838467166	1.039991962
RPL7L1	unnamed (-96454), unnamed (+10830)	0.84243443	1.040120955
CREB1	unnamed (+132)	0.745523554	1.04025329
RBM14	unnamed (+59)	0.844187952	1.040271468
SPRED1	unnamed (-1128)	0.501341503	1.04051069
SCAF11	unnamed (+86)	#N/A	1.040836034
DUSP3	unnamed (+388)	0.866816132	1.040872336
ATF6B	unnamed (-2054)	#N/A	1.041247859
GPT2	unnamed (-94348)	#N/A	1.041422566
HIST1H2BD	unnamed (-530)	1.738511051	1.041565688
POLR2E	unnamed (-254)	0.798596503	1.041809095
RPL15	unnamed (-80)	0.745626263	1.041996222
GIT2	unnamed (-2758)	1.172426059	1.04206429
PTPN23	unnamed (+253)	#N/A	1.042208533
ASF1B	unnamed (-614)	1.090216442	1.042378287
PPM1K	unnamed (+19)	#N/A	1.043214275
EDAR	unnamed (+202702)	#N/A	1.04324064
ZFP30	unnamed (-637)	#N/A	1.043452786
LARP1B	unnamed (-166)	0.749760601	1.04375357
KLC4	unnamed (-370)	#N/A	1.043980404
ARL2BP	unnamed (-171)	1.45262181	1.044797315
WDR4	unnamed (-49)	0.568891934	1.045421569
DNAJA1	unnamed (-42)	1.06123954	1.046432448
TRNAU1AP	unnamed (+28924)	1.004844359	1.04671441
CTNNB1	unnamed (+7)	0.644782874	1.047472257
HSD3B7	unnamed (-94)	1.208866191	1.047843483
P4HB	unnamed (-331)	1.421198579	1.047862832
SPG21	unnamed (-96)	0.846078608	1.047869517
FGFR1OP2	unnamed (-90)	0.275453168	1.048125631
YARS2	unnamed (+656)	0.886846264	1.048357129
SUCLG1	unnamed (-44)	1.108401675	1.048569617
NRG2	unnamed (-64357), unnamed (+74360)	#N/A	1.048764407
C17orf81	unnamed (+55)	0.62156916	1.048842594
NAA35	unnamed (-108)	0.959380225	1.048950896
EXOC8	unnamed (-404), unnamed (+304)	1.027903597	1.049244216
D4S234E	unnamed (-50440)	#N/A	1.049373464
GRAP	unnamed (+42299)	#N/A	1.049557336
GCN1L1	unnamed (+71)	0.911591182	1.049737358
FBXO45	unnamed (-260)	0.736192352	1.050099765
ESD	unnamed (-30)	0.969626582	1.050392247
LIN7C	unnamed (-126)	0.328685272	1.05110069
GPN3	unnamed (-589), unnamed (+367)	0.809556866	1.051468305
C1orf49	unnamed (+29447)	#N/A	1.051540616
PPM1L	unnamed (-1090)	0.693995734	1.051797098
CCNA2	unnamed (-21)	0.684460919	1.051927067
FGF11	unnamed (-4047)	#N/A	1.052138811
LRSAM1	unnamed (-746)	#N/A	1.052331901
LFNG	unnamed (-68230)	#N/A	1.052527113
LAMTOR2	unnamed (-195)	#N/A	1.052586631

ABCD1	unnamed (-423)	1.311541064	1.052871517
SPSB1	unnamed (-25508), unnamed (+136070)	#N/A	1.053071577
RPL23	unnamed (+248), unnamed (+28301)	0.854254373	1.053546447
PIF1	unnamed (-165)	#N/A	1.054092793
MXI1	unnamed (+3628)	0.537298283	1.054605768
SUGT1	unnamed (-127)	0.797185223	1.054690254
RPS7	unnamed (+48)	0.972600426	1.054910563
POLR2L	unnamed (-146)	0.687567023	1.05498028
NFE2L2	unnamed (+1529)	1.076840028	1.055054051
TIMM17A	unnamed (-78)	1.141434954	1.055814606
C21orf2	unnamed (+2)	#N/A	1.05587672
VRK2	unnamed (-116)	0.962594591	1.055936784
MED23	unnamed (-74)	1.450974727	1.056043308
SETD8	unnamed (-18640)	0.825659945	1.056282624
TCTEX1D2	unnamed (+99)	1.0686361	1.056389589
ELP4	unnamed (-4)	0.951409863	1.056526992
PTX3	unnamed (-261606)	#N/A	1.056981685
GBF1	unnamed (-80)	0.92566458	1.057373655
SUCNR1	unnamed (+395574)	#N/A	1.057385218
C16orf79	unnamed (-4196)	#N/A	1.058186337
ZFAND5	unnamed (-249)	1.257380635	1.058389508
RNF13	unnamed (+419)	1.211966663	1.058674558
THTPA	unnamed (-4171), unnamed (+286)	0.62056638	1.058816484
C5orf41	unnamed (-64)	0.700115489	1.058913173
JMJD1C	unnamed (+383)	0.955249442	1.058999067
SLC35E2	unnamed (-340)	#N/A	1.059151284
SNRPG	unnamed (+88)	0.889792274	1.060389754
NUDT5	unnamed (+191)	0.718874254	1.060721717
SLC25A5	unnamed (+97074)	0.969081832	1.06072319
STX12	unnamed (-47591)	1.062239544	1.060801257
MFAP3	unnamed (-203)	0.538615583	1.061066689
SERPINF2	unnamed (-26414)	#N/A	1.061127353
PPM1F	unnamed (+14436)	0.869654278	1.061520974
EP400	unnamed (-16)	1.003278348	1.061568409
TP53INP2	unnamed (-333), unnamed (+35)	2.087748273	1.061815533
TMEM208	unnamed (-149)	1.18109656	1.061841582
NUP160	unnamed (+81227)	0.518014693	1.06206401
KCTD18	unnamed (+2)	#N/A	1.062166677
DCTN2	unnamed (-56)	1.035226234	1.062598222
DAXX	unnamed (-60)	1.277245379	1.062624891
TRPM6	unnamed (-140879)	#N/A	1.062817496
NPPB	unnamed (-49426)	#N/A	1.06326019
TRAPPC4	unnamed (-143)	0.756008055	1.063542139
TSR1	unnamed (+812)	0.814109391	1.06412132
MRPS28	unnamed (+121)	0.637890196	1.064179882
MKLN1	unnamed (-220286)	0.790645285	1.06433841
MEF2D	unnamed (-4635)	0.916950599	1.064479364
PNRC2	unnamed (+104)	1.403623773	1.064798954
KCTD7	unnamed (-135333), unnamed (-348), unnamed (+25600)	#N/A	1.065590641
NRXN2	unnamed (-329)	#N/A	1.066300561

BRF1	unnamed (+234)	#N/A	1.066399176
SLC25A3	unnamed (-163)	1.063517338	1.066552026
IREB2	unnamed (+187)	0.854839161	1.066606148
ANKS1A	unnamed (-1184)	0.986552349	1.066641514
TSTD2	unnamed (+478)	#N/A	1.066984321
MRRF	unnamed (+292)	0.731862271	1.067419377
OSGEP	unnamed (+173)	#N/A	1.067762903
C1orf27	unnamed (-4116)	1.329868987	1.067765225
CTF1	unnamed (-2304)	#N/A	1.067874466
DLGAP4	unnamed (+93958)	1.435313627	1.06804686
ENOX1	unnamed (-250087)	1.169083923	1.068401287
MARCH1	unnamed (+888711)	#N/A	1.068521975
FAM57B	unnamed (-22126)	#N/A	1.06940843
AKAP1	unnamed (-364)	1.115952918	1.069534287
RAD51C	unnamed (+99)	1.27913974	1.069789815
ZW10	unnamed (+2)	1.10057587	1.06988076
MAD2L1	unnamed (-216)	0.950558684	1.070043292
TMEM194A	unnamed (-197)	0.961225512	1.070465928
ADAT1	unnamed (+85)	#N/A	1.070499616
SEMA4G	unnamed (-59151)	#N/A	1.070700068
STK10	unnamed (-371)	1.010255239	1.070776922
C7orf45	unnamed (-2400)	#N/A	1.070786991
ERLIN2	unnamed (-63)	0.853232853	1.070862604
BCL6	unnamed (+81)	0.926501433	1.071363365
GGH	unnamed (-399), unnamed (+275)	0.966114449	1.071498077
ZCCHC3	unnamed (-6872), unnamed (-233)	1.060762289	1.071653128
MYO19	unnamed (-49)	0.878312819	1.071931598
DHFRL1	unnamed (+92)	#N/A	1.072133497
TOB1	unnamed (+38)	1.420869457	1.072460082
ERAP2	unnamed (+59192)	#N/A	1.072515424
KIF26B	unnamed (-183926)	#N/A	1.072688177
MTBP	unnamed (-106)	#N/A	1.07280295
PPIL3	unnamed (-327)	1.282557855	1.07317264
PPP2R5E	unnamed (-82)	0.58659673	1.073219345
EPB41	unnamed (-231)	1.369946188	1.073425148
SPATA2	unnamed (-242)	#N/A	1.073648976
ETFA	unnamed (-80)	0.950482677	1.074082841
PAXIP1	unnamed (-284)	1.187799722	1.074538767
CSDE1	unnamed (+79)	0.714728452	1.074613805
SRP19	unnamed (+79)	1.235515637	1.074762777
CTSA	unnamed (+308)	1.258294094	1.075848522
SLC10A7	unnamed (-434)	#N/A	1.076033803
C11orf24	unnamed (-67)	1.185855692	1.076587746
EPC2	unnamed (-278)	0.866070111	1.076724129
VDAC3	unnamed (-149)	0.973378089	1.07728053
ATG9A	unnamed (-62)	1.108289873	1.077374202
PYGO2	unnamed (-176)	1.028216718	1.077559943
ZSWIM5	unnamed (-97456)	#N/A	1.07788719
TRIM24	unnamed (-528)	0.688471413	1.07818023
CLPB	unnamed (+134)	0.925549711	1.078244867
ALG10B	unnamed (-75)	0.878898417	1.078416845

C22orf23	unnamed (-11)	#N/A	1.078437488
RAD51	unnamed (-196)	1.439286469	1.078909248
MRPS7	unnamed (+8)	0.701447299	1.079257791
FOXRED1	unnamed (+26)	1.363081362	1.079600091
EEFSEC	unnamed (-126)	0.869427105	1.079603852
SLC26A8	unnamed (-3183)	#N/A	1.079640403
SEP15	unnamed (-1035)	0.920862197	1.080041816
HSPA13	unnamed (-189)	0.577509804	1.080696354
ZNF219	unnamed (-5080)	#N/A	1.080823342
CAMLG	unnamed (-385)	1.100477281	1.080832987
NIF3L1	unnamed (+126)	1.235735237	1.080876014
ZNF410	unnamed (-131)	1.091810753	1.081008412
C21orf67	unnamed (-21)	#N/A	1.081192924
CLPX	unnamed (+73), unnamed (+717)	0.974254795	1.081244794
SF1	unnamed (-511)	1.05424675	1.081409348
ZNF547	unnamed (-120)	#N/A	1.081582139
CENPL	unnamed (-516)	1.033584836	1.081711341
BCL2L11	unnamed (-633), unnamed (+149)	0.604164567	1.081712175
TCTE3	unnamed (+129)	0.800934109	1.082184793
KDM3A	unnamed (+121595)	0.912235218	1.082441115
NOL9	unnamed (+47)	0.965640668	1.08264033
SLC5A10	unnamed (+52559)	#N/A	1.08304834
PSMD5	unnamed (-51)	0.885967641	1.083604378
PARK7	unnamed (-227)	0.881053381	1.083792301
LMF2	unnamed (-530)	0.993448536	1.083857296
G6PC3	unnamed (0)	0.901751851	1.08395794
SPNS1	unnamed (-25)	1.165338143	1.084406172
NEK8	unnamed (-2034)	#N/A	1.084572059
SCN4B	unnamed (+7461)	#N/A	1.084607186
PLCB3	unnamed (+18775)	#N/A	1.084617341
MINK1	unnamed (-89)	1.204407581	1.0846629
SLC36A1	unnamed (+147)	#N/A	1.084990806
CRIP1	unnamed (+3951)	1.02962232	1.085014534
PRPF40B	unnamed (-7530)	#N/A	1.08506172
ANKRD17	unnamed (-263)	0.96536193	1.085128787
ZBED5	unnamed (+225)	0.806277212	1.085144821
ATF7	unnamed (+89)	0.464607366	1.085236438
HEBP2	unnamed (+369325)	0.737026485	1.085591819
B3GNT4	unnamed (+22391)	#N/A	1.086053203
RFX7	unnamed (-122090)	0.830202814	1.086216597
HNRNPM	unnamed (-125)	0.968873021	1.086256683
ZMYM3	unnamed (-16)	0.619698912	1.087257189
NUP214	unnamed (-20)	0.90550352	1.0872951
GALNT1	unnamed (+318148), unnamed (+413034)	0.69622851	1.087565854
ACBD4	unnamed (-2460)	#N/A	1.088142393
RNPC3	unnamed (-34)	0.850821431	1.088478963
RET	unnamed (+28584)	#N/A	1.088858684
ZNF434	unnamed (+29)	1.120501905	1.089021102
FNTA	unnamed (-152)	1.038600046	1.089252377
C14orf2	unnamed (-17)	0.798781684	1.089442031
GJD2	unnamed (+166095)	#N/A	1.089898969

DBR1	unnamed (+188)	1.219967969	1.090651719
NPR3	unnamed (-1306)	0.648323537	1.090929547
RPL3	unnamed (+8575)	0.884165682	1.091447761
TMEM39A	unnamed (-113)	1.037261441	1.091558837
VPS37C	unnamed (+31456)	1.656077276	1.091755241
SAMD8	unnamed (-272)	1.200085941	1.091953077
ZNF740	unnamed (-139)	0.742758439	1.092026045
KDM2A	unnamed (-37616)	1.285363545	1.092090174
FLJ43860	unnamed (+75557)	#N/A	1.092263323
TBCCD1	unnamed (-3211), unnamed (-95)	1.440626361	1.092609424
CYC1	unnamed (+13)	0.905895846	1.092616884
TUBA1A	unnamed (+2492)	1.218085669	1.092864217
CREB3L2	unnamed (-273)	1.212548798	1.093264474
HES1	unnamed (+179497)	1.229763837	1.093479912
ALS2	unnamed (-76)	#N/A	1.093536636
MRPL48	unnamed (-8276)	1.040717586	1.093754897
SYVN1	unnamed (-523)	1.221452262	1.094213404
POLR3D	unnamed (-19)	0.697019202	1.094268313
MTAP	unnamed (-375)	0.784243549	1.094292892
SIK3	unnamed (-390)	0.624484779	1.09516604
FRAT2	unnamed (-128)	1.003772952	1.095561193
RBM15	unnamed (-446)	0.541964573	1.095668336
CYB561D1	unnamed (-9865), unnamed (-180)	1.201487561	1.095859684
HES7	unnamed (-15043)	#N/A	1.096550938
ADCY1	unnamed (+194543)	#N/A	1.096564131
ZNF77	unnamed (-316)	#N/A	1.096635694
RTTN	unnamed (-134)	0.952532925	1.096767065
TPD52L2	unnamed (-60)	1.217906816	1.09695619
EFCAB11	unnamed (-1188)	#N/A	1.097216837
ACTG1	unnamed (-6607), unnamed (+28432)	1.051272539	1.097264577
METTL5	unnamed (-2585), unnamed (-128)	0.964483065	1.097332971
BHLHE23	unnamed (-50182)	#N/A	1.097661306
BCL7C	unnamed (-225)	0.693465058	1.098147724
C6orf203	unnamed (+204)	1.025100427	1.098211079
TAB3	unnamed (+13)	0.861115077	1.098326369
FSCN2	unnamed (-8918)	#N/A	1.098479933
COL9A1	unnamed (-109952)	#N/A	1.098519203
ZDHHC22	unnamed (+43568)	#N/A	1.098843354
VPS29	unnamed (-151)	1.056256113	1.099023723
STX4	unnamed (-192)	1.618665322	1.099330099
C20orf11	unnamed (-83)	1.024457005	1.099897178
ABCC10	unnamed (+87)	1.429695983	1.100056641
INTS4	unnamed (-170)	0.947815495	1.100148708
RBPJ	unnamed (-1102), unnamed (+49)	0.681410453	1.101112797
APEX1	unnamed (-196)	0.558314716	1.101142395
SNF8	unnamed (-104)	0.945262726	1.101215074
HYAL2	unnamed (+1229)	0.902464993	1.101803231
PKN2	unnamed (+6)	0.740742304	1.102236256
ANAPC10	unnamed (+61)	0.846621986	1.102494773
IBA57	unnamed (+156)	#N/A	1.102679344
TXNDC9	unnamed (-15)	0.812213073	1.103005437

POLE3	unnamed (+161)	1.128100743	1.103523844
RPL13	unnamed (+5069)	0.831541557	1.103544819
KLC2	unnamed (-34859)	#N/A	1.104177515
SPRYD4	unnamed (-4056)	0.940158521	1.10497139
VIPR2	unnamed (+288451)	#N/A	1.105073781
NOP58	unnamed (+10)	0.93580932	1.105297425
MRPL2	unnamed (-17)	1.088392979	1.105337171
LMBR1	unnamed (-260)	0.490804689	1.106216583
FBXO24	unnamed (-3228)	#N/A	1.106635663
CNNM2	unnamed (+10)	1.201174651	1.107074619
NRD1	unnamed (-14)	1.214746435	1.107698737
INO80E	unnamed (-112)	1.258058119	1.108133187
SNAPC2	unnamed (+28)	#N/A	1.108631738
CENPC1	unnamed (+143)	0.681011954	1.108638291
CAND1	unnamed (-84)	0.938697516	1.110094886
ZNF292	unnamed (-25)	0.78925794	1.110292046
HHIPL1	unnamed (-40721)	#N/A	1.111015217
CDK5R1	unnamed (+335554)	#N/A	1.111326825
HERPUD2	unnamed (+95)	1.212954426	1.111679522
TARDBP	unnamed (-143)	0.92239421	1.112470494
MLL5	unnamed (-705)	0.779178982	1.112700867
LIM2	unnamed (-6460)	#N/A	1.11314393
DPAGT1	unnamed (-81)	0.823543975	1.113178207
GSK3A	unnamed (-407)	0.823330949	1.113308239
C3orf20	unnamed (-23493)	#N/A	1.113854995
TMEM18	unnamed (-106)	0.973992118	1.113906514
WDR33	unnamed (-76)	0.644949807	1.114261286
GDE1	unnamed (+66)	0.940705102	1.114557993
HOMER2	unnamed (-59038), unnamed (-33320)	#N/A	1.114635014
GPR153	unnamed (+860)	#N/A	1.114741941
RAB2B	unnamed (+20462)	1.0055591	1.115728669
PLD2	unnamed (-217)	#N/A	1.116669368
ASMTL	unnamed (-33)	0.707166179	1.116842186
TMEM116	unnamed (-18)	1.211484406	1.11713112
DDX51	unnamed (-78)	0.910751607	1.117183423
ARHGAP1	unnamed (-434)	1.451827126	1.11768018
TRIP10	unnamed (-2020)	0.852735155	1.117877238
ESYT2	unnamed (-26879)	1.318322365	1.118172394
ZNF200	unnamed (+223)	1.067990856	1.118230153
METTL23	unnamed (-80)	#N/A	1.118703471
FIZ1	unnamed (+12106)	0.912346358	1.118864817
BAG2	unnamed (+374)	0.782494074	1.119016998
CUL5	unnamed (+718)	1.120479267	1.119173038
CSNK2B	unnamed (-99)	0.987104478	1.119472635
ZNF341	unnamed (-70)	0.753369052	1.11965741
VAC14	unnamed (-72)	#N/A	1.119664648
C6orf70	unnamed (-212)	#N/A	1.119694561
TP53RK	unnamed (+172)	1.106168231	1.119742965
XKR6	unnamed (+361390)	#N/A	1.120390334
MDH2	unnamed (-209), unnamed (+118535), unnamed (+243706)	1.165534088	1.120755503

FTSJD2	unnamed (+58)	0.796705675	1.120983167
CDC25C	unnamed (-6034)	0.656331673	1.121402248
MMP23B	unnamed (+23003)	#N/A	1.122842578
PHF1	unnamed (-900)	1.15741198	1.123072949
POLR2F	unnamed (-9)	1.092077797	1.123138789
POLR2F	unnamed (-9)	1.092077797	1.123138789
BLK	unnamed (-27136)	#N/A	1.123790522
CDC40	unnamed (-298)	1.009752424	1.123852968
SMARCA5	unnamed (-130)	0.992516152	1.124099358
NPFFR1	unnamed (-80985)	#N/A	1.124147263
MUL1	unnamed (+53)	#N/A	1.124527758
ASB13	unnamed (-18043), unnamed (+43303)	0.898178499	1.1245519
GTF3C2	unnamed (+120)	0.935202915	1.124977511
ZNF691	unnamed (+78045)	#N/A	1.125075978
POLR2A	unnamed (+5)	1.000426601	1.125076056
SMARCC2	unnamed (-753)	0.893766396	1.125365601
UBE3A	unnamed (+26)	0.601382101	1.125435511
ZNF91	unnamed (+738)	#N/A	1.125793397
C1orf123	unnamed (+249)	1.056256308	1.126137782
METTL21B	unnamed (-153)	#N/A	1.127101913
USP22	unnamed (-754), unnamed (+99)	1.244263696	1.12716397
MON2	unnamed (+135897)	1.169767311	1.127215369
RBMXL1	unnamed (+301)	0.790469954	1.128065469
ZBTB5	unnamed (-167)	1.147161122	1.128261984
SPHK2	unnamed (-169)	#N/A	1.128345227
STARD13	unnamed (-257167)	#N/A	1.128383609
ATXN1L	unnamed (-32)	0.993884804	1.128638489
XRCC6	unnamed (+80)	0.922081366	1.129026094
C4orf29	unnamed (-73)	1.041487969	1.129234537
AURKAIP1	unnamed (-174)	0.881119814	1.129444538
RIC8B	unnamed (-289)	1.031560484	1.130633614
C20orf132	unnamed (+415)	#N/A	1.130640572
RPS15A	unnamed (-34), unnamed (+1006)	1.028314366	1.130717484
NPC1L1	unnamed (+50191)	#N/A	1.130792602
RBBP4	unnamed (-338)	0.814879348	1.130819753
DUS1L	unnamed (-104)	0.839071792	1.131625588
CDK13	unnamed (-216996), unnamed (-176)	0.647673201	1.131819841
SARS2	unnamed (+336)	#N/A	1.131887044
PSTK	unnamed (+562)	1.02317496	1.132956897
BTG1	unnamed (-180)	1.54721188	1.133425933
COX7B	unnamed (+104)	1.089836091	1.133472695
NUDT2	unnamed (-297)	0.966682262	1.133994683
ZNF770	unnamed (-557967), unnamed (-11)	0.902601263	1.134207255
GPR97	unnamed (-19027)	#N/A	1.134887393
GPR176	unnamed (+137952)	1.979514925	1.13499599
RING1	unnamed (-70)	0.801389101	1.135563918
OGDH	unnamed (-20)	1.538342967	1.135730205
MFSD4	unnamed (+62214)	#N/A	1.136157548
CLPTM1L	unnamed (-298)	1.235439279	1.136273923
PRKCSH	unnamed (-58)	1.242716871	1.136686894
METTL19	unnamed (-165)	#N/A	1.137102887

CDK16	unnamed (-4553)	0.982225039	1.137555282
TRAF3IP3	unnamed (+28456)	#N/A	1.138367548
TDRKH	unnamed (-955)	#N/A	1.138400766
PDPR	unnamed (+522)	0.976731047	1.138455473
CIAPIN1	unnamed (-92)	1.009471014	1.138795157
ATN1	unnamed (+13557)	#N/A	1.139574148
ZBTB2	unnamed (+736)	0.983915718	1.139636027
COQ3	unnamed (+276)	0.448289976	1.140495898
NOTCH1	unnamed (-98852), unnamed (-85747)	0.813027067	1.140778874
SFXN5	unnamed (+147)	#N/A	1.141644971
USP15	unnamed (-65)	0.684184616	1.141984775
CCNL2	unnamed (-218)	0.744323858	1.142970911
USP2	unnamed (-53)	#N/A	1.142998719
ZWILCH	unnamed (+10)	1.42062684	1.143386157
RAB21	unnamed (-143)	1.658920591	1.143732354
USP30	unnamed (-88)	0.902498509	1.143867187
C16orf5	unnamed (-77560)	1.290045911	1.143892091
MAPK14	unnamed (+34)	0.670142468	1.145347888
SAMD11	unnamed (-147033)	1.144821205	1.14597804
TCTN1	unnamed (+51)	#N/A	1.146574939
CEP85	unnamed (-265)	#N/A	1.14661087
REPS1	unnamed (-47)	0.979056714	1.146658697
SMG7	unnamed (-574)	0.800145812	1.146704078
PRPF31	unnamed (+110)	0.691692468	1.147121407
LZTR1	unnamed (-228)	0.985370123	1.147834587
PSMD11	unnamed (-105)	0.832349068	1.148702303
SRSF7	unnamed (+182)	0.810485769	1.149040593
ZNF384	unnamed (+288)	1.155123797	1.15006602
PPP1R15A	unnamed (-79), unnamed (+720)	1.745968372	1.150546027
CSAD	unnamed (+297)	0.978862834	1.150756204
ZNF689	unnamed (-40241)	0.960029875	1.151155762
TMEM87B	unnamed (+387)	0.889268986	1.151264834
TTLL12	unnamed (-463)	0.822330095	1.151364514
CAPNS1	unnamed (-11888), unnamed (-82)	0.968197688	1.151462458
DDX28	unnamed (+625)	0.926594726	1.152045848
CTAGE5	unnamed (+44), unnamed (+165359)	0.977210416	1.152529658
KIF2C	unnamed (-140)	1.09464208	1.152581301
NCK1	unnamed (+347)	1.410773149	1.152609469
GALC	unnamed (+221920)	#N/A	1.152924078
PKIG	unnamed (+41)	#N/A	1.153790293
SLC25A32	unnamed (-110)	0.570008655	1.154137828
C8orf76	unnamed (+84141)	1.270400105	1.154732838
CCNK	unnamed (+45), unnamed (+123020)	1.257432022	1.15475912
GDI2	unnamed (-390), unnamed (+128911)	0.937421692	1.155494061
COMTD1	unnamed (-72)	0.669067074	1.156275165
MUC5B	unnamed (+41535)	#N/A	1.156542752
ZNF543	unnamed (-85)	#N/A	1.156828469
IL10RA	unnamed (-39305)	#N/A	1.15785105
AIDA	unnamed (+68257)	0.338087605	1.158045066
SCAND2	unnamed (+23049)	#N/A	1.158459897
SLC38A6	unnamed (-70)	0.989851428	1.158600364

MAN2C1	unnamed (+52)	0.993302056	1.158774237
RQCD1	unnamed (-158)	0.54844451	1.158814084
ATP6V1H	unnamed (+30)	1.210975341	1.15884306
CENPQ	unnamed (-5)	0.693450694	1.159113003
C6orf228	unnamed (-233)	#N/A	1.159182585
KIF5A	unnamed (-2813)	#N/A	1.159758526
MAFF	unnamed (-1236)	1.170362389	1.160058967
P4HA1	unnamed (+36)	0.99345673	1.160187063
COQ9	unnamed (+124)	0.9622487	1.160628621
HNRNPH1	unnamed (-166)	0.761543412	1.161228033
TM9SF4	unnamed (+43)	0.675611305	1.161321159
DFFA	unnamed (-84)	0.84212166	1.161599988
COPS7B	unnamed (+97)	#N/A	1.162051707
SYNGR4	unnamed (-1112), unnamed (-97)	#N/A	1.162547345
NAA60	unnamed (-214)	#N/A	1.162598737
SCYL3	unnamed (-250)	1.191142105	1.163298433
SEC61A1	unnamed (-192)	1.486701166	1.163940123
MTMR2	unnamed (-319236)	1.092917116	1.164149037
PBLD	unnamed (+449)	#N/A	1.164230303
DPEP3	unnamed (-12903)	#N/A	1.164579398
RNF139	unnamed (-253)	0.770043718	1.164738732
CELSR2	unnamed (+13782)	0.977493244	1.165367634
CCNC	unnamed (+271)	0.825201871	1.165551169
KDM3B	unnamed (-14735), unnamed (-103)	1.116857752	1.166094967
PQLC2	unnamed (-143)	1.411798871	1.16628886
TSNAXIP1	unnamed (+26880)	#N/A	1.166836616
CNTNAP1	unnamed (-4047)	#N/A	1.167246699
TYROBP	unnamed (-23458)	#N/A	1.167759651
NDUFB9	unnamed (-172)	0.937924339	1.168195749
HGS	unnamed (+19421)	1.142417261	1.168272183
KLF10	unnamed (-151903)	1.701790653	1.1685548
TMEM201	unnamed (+38281)	#N/A	1.168912444
CD55	unnamed (-127)	0.923013276	1.169259268
SERP1	unnamed (+565)	0.993379339	1.17064946
IFRD1	unnamed (+27483)	1.307302039	1.170886091
EFNA1	unnamed (-42977)	0.817655001	1.170896881
SEC24C	unnamed (-304)	1.230744918	1.171043378
ISCU	unnamed (-35)	1.02345995	1.171295674
SRSF9	unnamed (-254)	1.04842707	1.17147616
ATP6V0D1	unnamed (-98)	1.185507331	1.171505564
MYH7B	unnamed (-82)	#N/A	1.171674669
NDUFS3	unnamed (-67)	0.851456922	1.171753807
ZNF783	unnamed (-261)	#N/A	1.171896167
NUMBL	unnamed (+27687)	1.7039598	1.172295536
RNASE4	unnamed (-4693)	#N/A	1.172569442
ACOX3	unnamed (+85), unnamed (+12228)	#N/A	1.173105284
NLRC5	unnamed (-52365)	#N/A	1.173519461
PRR3	unnamed (+238)	0.732921913	1.173856969
NDOR1	unnamed (+7)	#N/A	1.174733575
RER1	unnamed (-122)	0.969628162	1.174887434
AJUBA	unnamed (-24171)	#N/A	1.175107183

POLR3G	unnamed (-492)	0.718960363	1.175127402
TSPAN4	unnamed (-149)	0.733015667	1.175460144
STAT4	unnamed (+131197)	#N/A	1.17555359
CSH2	unnamed (+24642)	#N/A	1.175585324
PTEN	unnamed (-1146), unnamed (-117)	0.470551047	1.176349842
PPM1D	unnamed (+3)	0.823706196	1.176871558
NR6A1	unnamed (-255)	#N/A	1.17700062
TP73	unnamed (-2507)	#N/A	1.177180716
TAS1R1	unnamed (-727)	#N/A	1.177274237
AFAP1L2	unnamed (-122214)	#N/A	1.177353274
MGRN1	unnamed (-8449), unnamed (-326)	1.214466648	1.177507372
CREB3L4	unnamed (-4109), unnamed (-346)	0.432842716	1.178392345
PELP1	unnamed (+194)	1.192468709	1.178649026
GLYCTK	unnamed (+152)	#N/A	1.179279289
MLL2	unnamed (-5510)	1.191680652	1.180943505
ZYG11A	unnamed (+333)	0.727151834	1.181381855
RNF7	unnamed (+18)	0.908961009	1.181478513
BTD	unnamed (+201)	1.022164359	1.181522432
TOLLIP	unnamed (+45062)	#N/A	1.181559954
ARFGAP1	unnamed (-44)	1.012603875	1.182025163
HNRNPUL1	unnamed (-305)	1.013816303	1.18222408
PTPRS	unnamed (+292390)	1.082223993	1.182292925
MAPK1	unnamed (-70844)	0.899514768	1.182490821
FAM186B	unnamed (-17365)	#N/A	1.182598969
MED22	unnamed (-95)	0.745356356	1.182937559
ATF4	unnamed (-357)	1.142168857	1.183521192
PITX3	unnamed (-3944)	#N/A	1.183597047
TMIGD2	unnamed (+55349)	#N/A	1.18360516
CBY1	unnamed (-159)	#N/A	1.184627693
TMEM222	unnamed (+76)	0.924049138	1.184870912
CCT2	unnamed (+129)	0.97062664	1.185261509
NLE1	unnamed (-89), unnamed (+22436)	#N/A	1.186023218
NFX1	unnamed (-8)	0.785884294	1.186650442
SLC44A1	unnamed (-301)	1.001324002	1.187768376
GUCY2F	unnamed (+427665)	#N/A	1.187829876
F8	unnamed (-4417)	1.437207118	1.187832848
IPP	unnamed (+29)	1.055833736	1.187833911
CROCC	unnamed (-17066)	#N/A	1.189077251
MYLIP	unnamed (+349)	0.835087109	1.189725989
HNRNPAB	unnamed (-125)	1.399841601	1.190186979
SERTAD3	unnamed (+833)	1.184572074	1.190243303
DUS2L	unnamed (-59)	1.0568919	1.190318781
STAM2	unnamed (+108)	1.08929218	1.191864312
TBRG4	unnamed (+1)	0.918502074	1.192051541
PSPH	unnamed (+106), unnamed (+17326)	1.040877413	1.192128953
VDAC1	unnamed (-97035)	0.927240251	1.193112232
USP3	unnamed (+18)	0.835159398	1.193331972
PKNOX1	unnamed (-202)	1.085686588	1.194014946
PRKACG	unnamed (+234148)	#N/A	1.194044201
BCL7B	unnamed (+481)	0.766884209	1.194301338
MTERFD1	unnamed (-152)	1.325991147	1.195071947

PANX1	unnamed (+269), unnamed (+649)	1.343858505	1.195383677
WARS2	unnamed (+184)	0.898783569	1.195504745
HN1	unnamed (+288)	1.048403952	1.19624896
CSDA	unnamed (+1120)	0.483652348	1.197031275
TIMM22	unnamed (-231)	1.044436587	1.19728185
CHD9	unnamed (-205)	0.642850797	1.19732016
CDIPT	unnamed (-190)	0.934985597	1.19733861
C15orf61	unnamed (+459)	0.969275638	1.197530997
ARHGEF12	unnamed (-731)	0.86582239	1.197782389
BARX1	unnamed (-75383)	#N/A	1.197845024
ZNF259	unnamed (+15002)	1.181312399	1.198467339
ATP5F1	unnamed (+301)	0.950392337	1.19883965
RBKS	unnamed (-795)	1.003701765	1.199040324
FBXL19	unnamed (-1737)	#N/A	1.199147076
DCT	unnamed (-70159)	#N/A	1.199256981
SHC1	unnamed (-3576)	0.971688205	1.200152549
MECOM	unnamed (-101374)	#N/A	1.200171553
RFX1	unnamed (-358)	0.958228059	1.200179892
EIF2D	unnamed (-1)	#N/A	1.200790345
CNTNAP3B	unnamed (+542559)	#N/A	1.200948052
BAI2	unnamed (-25367)	#N/A	1.201122499
SSU72	unnamed (+68)	0.965060224	1.201446966
RBFOX2	unnamed (-95630)	1.167345417	1.202192078
CHD2	unnamed (-91050), unnamed (+3957)	0.312092403	1.202472803
TMPO	unnamed (+13)	0.607783236	1.202473529
LBX1	unnamed (+15126)	#N/A	1.203378272
IGHMBP2	unnamed (+34)	#N/A	1.203428255
KCTD5	unnamed (-10)	1.221729105	1.203839278
HNRNPA3	unnamed (+50908)	0.992627397	1.20392552
SIVA1	unnamed (-22)	0.846816414	1.203962031
TLX1	unnamed (-63262), unnamed (+82530)	#N/A	1.204126071
PTPDC1	unnamed (-53720), unnamed (+82722)	1.017005494	1.20431213
LEFTY2	unnamed (-58070)	#N/A	1.204511375
CNIH2	unnamed (+4434)	#N/A	1.204966245
CYCS	unnamed (-106)	0.731068951	1.205025749
PPP2R3B	unnamed (-142)	#N/A	1.205467954
ZNF408	unnamed (+237)	#N/A	1.205919142
RHOC	unnamed (-496)	1.088611076	1.206851749
CHST12	unnamed (+52), unnamed (+48054)	1.05939252	1.207709596
PURB	unnamed (-309)	0.503226935	1.208239131
WHSC2	unnamed (-31)	0.78043904	1.208579681
BRAT1	unnamed (+96)	#N/A	1.208742859
SLC2A1	unnamed (+34558)	0.716844009	1.20893285
PDE8A	unnamed (-1551), unnamed (-609)	0.852608057	1.209079753
PICK1	unnamed (-4801)	#N/A	1.209678711
FAM40A	unnamed (-92)	1.130785628	1.210348938
HNRNPL	unnamed (-481)	0.899825797	1.2103493
RAD1	unnamed (+68)	0.955051038	1.210493352
GATAD2B	unnamed (-379)	1.02515647	1.21054106
DCAF10	unnamed (-107)	0.774032743	1.210610301
LRP10	unnamed (-18574)	1.67100641	1.210632542

KDM5A	unnamed (-65)	0.776625975	1.211018956
ADNP2	unnamed (-74)	0.7124639	1.21122494
ULK3	unnamed (-154)	0.629569054	1.211562774
TSSK4	unnamed (+7761)	#N/A	1.211642055
KIAA0182	unnamed (-367501)	0.655426929	1.211642713
NAA40	unnamed (-97)	1.076053058	1.211849131
APOB	unnamed (+244165)	#N/A	1.211856735
ALDOA	unnamed (-12682)	0.870036904	1.211918742
CALM1	unnamed (-13784), unnamed (+2), unnamed (+361633)	0.897096704	1.211922966
ING4	unnamed (-263)	#N/A	1.212767583
IGFBP1	unnamed (-119291)	#N/A	1.212830366
IFITM3	unnamed (-35266)	1.010311809	1.213108418
PRR24	unnamed (-458)	1.135473296	1.21345781
FBXL18	unnamed (-12366), unnamed (-60)	1.340794501	1.213621243
TMEM38A	unnamed (-911)	#N/A	1.214023108
NFIC	unnamed (+68532)	0.767446867	1.21413085
C2orf49	unnamed (-165)	0.74608866	1.214662769
CTNNA3	unnamed (-68384)	#N/A	1.215526912
MYL9	unnamed (-80491)	#N/A	1.215929482
ZMAT5	unnamed (-123)	0.947679967	1.216768921
CREG1	unnamed (-160955)	1.144049823	1.217035093
POLB	unnamed (+15)	0.669408907	1.217520206
AUP1	unnamed (+18)	0.817902143	1.218087481
MBOAT7	unnamed (-500)	1.226321989	1.218239164
RPS25	unnamed (-41)	0.94152716	1.218576287
BCAS3	unnamed (-260)	#N/A	1.218896309
C11orf31	unnamed (+114)	0.886294779	1.219429491
CCPG1	unnamed (-35)	0.917351675	1.220388224
TULP1	unnamed (-10179)	#N/A	1.220406538
STYK1	unnamed (-47942)	#N/A	1.220737917
TIGD5	unnamed (-344)	1.184532169	1.220912397
MICA	unnamed (-36552)	0.935551749	1.221408632
RAB7A	unnamed (-45176)	0.736286194	1.221647421
SNAPC4	unnamed (-1902)	#N/A	1.221832385
MIB2	unnamed (+54)	#N/A	1.221848945
ESRRA	unnamed (-942)	0.979493735	1.222162665
RSL24D1	unnamed (+108)	0.777826149	1.223445902
SLC25A28	unnamed (-566)	1.102803157	1.223447526
NDUFS2	unnamed (+288)	1.256173592	1.223848644
PRCD	unnamed (+17770)	#N/A	1.224603452
GUSB	unnamed (+75)	0.942929766	1.224692939
MTRF1L	unnamed (-151)	0.930803515	1.225104047
GSTCD	unnamed (-2024)	1.370834361	1.225335626
ERRFI1	unnamed (-194)	1.145212236	1.225416203
PARD6A	unnamed (+149)	#N/A	1.225983629
ECSIT	unnamed (+287)	0.961535095	1.226892717
HIST1H3C	unnamed (+9907)	#N/A	1.228237963
WDR5	unnamed (+28701)	0.859429505	1.22848818
DHX37	unnamed (+4), unnamed (+48414)	#N/A	1.229504825
KIAA1797	unnamed (+25865)	0.881668627	1.230411143

DCAF11	unnamed (+56)	0.809530696	1.230768845
LRTOMT	unnamed (+417)	#N/A	1.231091147
LRTOMT	unnamed (+417)	#N/A	1.231091147
GPSM3	unnamed (-515)	#N/A	1.231625996
B3GAT3	unnamed (-62)	#N/A	1.233358649
NCDN	unnamed (+204)	#N/A	1.233411833
PPP1R2	unnamed (-104)	0.698084314	1.233465577
H2AFJ	unnamed (-3955)	0.914188794	1.233858527
SHANK1	unnamed (+37314)	#N/A	1.233908763
NOP14	unnamed (+205)	0.929078285	1.234226435
SYNCRIP	unnamed (+249)	0.65635974	1.235991328
PHACTR4	unnamed (+136659)	1.22253818	1.236122456
TBCA	unnamed (+92)	0.937271099	1.236333765
C1RL	unnamed (+659)	1.132142202	1.236713873
ASNSD1	unnamed (-76)	0.668047395	1.237137278
FOXK1	unnamed (-40234)	1.153290436	1.238064054
PLAGL1	unnamed (+165293)	0.786041205	1.238251564
AP2A1	unnamed (-1023)	0.909336885	1.239340979
JOSD1	unnamed (-4994), unnamed (-697)	2.168837917	1.239424879
PRUNE	unnamed (-232)	0.739021527	1.23988108
PLXNB1	unnamed (-6533)	#N/A	1.240768696
STK40	unnamed (-477)	#N/A	1.241450714
C19orf24	unnamed (+259)	0.779618025	1.242198068
MAP2K1	unnamed (-150)	1.178755745	1.242997682
PHF21A	unnamed (-122006), unnamed (+16)	#N/A	1.24333684
NOL8	unnamed (+5)	1.000618471	1.243924844
TWISTNB	unnamed (-76)	1.078341412	1.244763412
FOXJ3	unnamed (-150)	1.33271507	1.244857192
ZMPSTE24	unnamed (+37)	1.137218925	1.244985011
DAPK3	unnamed (-1596)	1.281010325	1.245374094
LNPEP	unnamed (-23320)	0.867326303	1.245532661
ZNF136	unnamed (+33)	1.143516119	1.245797554
TMEM11	unnamed (+257)	0.925927442	1.245856114
TMEM11	unnamed (+257)	0.925927442	1.245856114
NSDHL	unnamed (+55)	1.097144902	1.246055507
ZNF420	unnamed (-69)	1.55385638	1.246193161
ALKBH2	unnamed (-119)	1.078308222	1.24649871
CENPB	unnamed (+30)	1.106362188	1.246771216
ABTB2	unnamed (+183441)	1.824554741	1.24746697
PDP1	unnamed (+73774)	1.30179666	1.248176875
DNAJB12	unnamed (+96)	0.716862168	1.24826947
RAB11B	unnamed (-204)	1.024264888	1.248308241
COBRA1	unnamed (-170)	1.163901823	1.249012904
ANGPTL6	unnamed (-3684)	#N/A	1.249760231
CHERP	unnamed (-148)	1.043983529	1.249812619
FBXO5	unnamed (-386)	0.706420876	1.250451093
CAMSAP2	unnamed (-229)	#N/A	1.250480273
C17orf101	unnamed (-124)	0.761147434	1.25048719
NAP1L4	unnamed (-382)	1.031834268	1.250671153
WNT11	unnamed (-112425)	#N/A	1.251103039
GPR137	unnamed (-14041)	1.088894531	1.251772678

TMED2	unnamed (-256)	0.972389348	1.251876162
C7orf70	unnamed (-118)	0.774616175	1.252042298
MDM2	unnamed (+576)	0.844039469	1.25218854
TBC1D19	unnamed (+91)	#N/A	1.252488461
GAPDH	unnamed (-398)	0.982983837	1.25361698
HIBCH	unnamed (+130)	0.660699903	1.253915095
ZNF79	unnamed (+84)	#N/A	1.254399907
METTL2B	unnamed (-2)	#N/A	1.255461455
PTP4A1	unnamed (+1277)	0.793384788	1.256645535
CACNA1E	unnamed (-293498)	#N/A	1.256893767
OFD1	unnamed (+112)	1.11228489	1.257119069
PHB	unnamed (-160)	0.938187611	1.257149725
DOLK	unnamed (+21)	1.302714635	1.257208426
UBL3	unnamed (+220)	0.692959407	1.25725635
ZSCAN16	unnamed (+12285)	#N/A	1.259334781
RPS6KA1	unnamed (+74989)	1.473007567	1.25972355
LSAMP	unnamed (+660853)	#N/A	1.259889719
RC3H2	unnamed (-14)	0.719274983	1.259928087
SLC25A33	unnamed (-110517), unnamed (-274)	0.734662353	1.259970447
FAM82A2	unnamed (-164)	1.04115857	1.260015581
MRPL14	unnamed (+52726)	0.7660574	1.260353433
DCAF13	unnamed (+636)	0.934620832	1.260749956
MRPL21	unnamed (-50)	0.765007284	1.261272056
HAUS1	unnamed (+69439)	0.833225721	1.262250583
RFT1	unnamed (+99)	0.67404597	1.264012446
AHSA1	unnamed (-195)	1.302430764	1.264111352
ORMDL3	unnamed (-97)	#N/A	1.264415309
SH3GLB1	unnamed (+45)	0.865785701	1.265014798
PTP4A3	unnamed (+9766)	0.753411185	1.265099386
FOSB	unnamed (-24427)	#N/A	1.265868885
HECA	unnamed (-146)	1.101029717	1.266878243
MBLAC1	unnamed (+458)	#N/A	1.267224874
TMCO1	unnamed (-97)	0.858060922	1.267943599
CHRM1	unnamed (+40377)	#N/A	1.268665354
C7orf49	unnamed (+15)	1.050425297	1.268909079
CPSF1	unnamed (-52)	0.920399982	1.269009743
B4GALT5	unnamed (+102647)	1.500766791	1.270381394
LONP1	unnamed (-291)	1.020401895	1.270444253
SLC35B4	unnamed (-19)	0.910154004	1.270635125
TBC1D17	unnamed (-272)	1.000864887	1.271316695
POP7	unnamed (-30)	1.042521891	1.272513849
ZNF746	unnamed (-127049)	1.683723249	1.272673233
TIMM13	unnamed (+336)	0.805878996	1.273395624
ZNF263	unnamed (-211)	1.261941224	1.273399757
H3F3B	unnamed (-63255)	1.291780022	1.273614874
SPATA5L1	unnamed (+33)	0.774692499	1.274335851
HDAC10	unnamed (+146)	#N/A	1.274901864
ACTR1B	unnamed (+731)	0.669591387	1.274992238
PPP6R1	unnamed (-759)	1.209992974	1.275013503
ABI1	unnamed (-172)	1.21439776	1.27550614
MLX	unnamed (-4998)	0.879046369	1.275816684

ASH1L	unnamed (-595)	0.825386988	1.275887991
MZT2A	unnamed (-351)	0.891783582	1.276295732
BRAF	unnamed (-275)	0.540694661	1.276662054
SLC5A6	unnamed (+40)	1.05165447	1.277226867
PDRG1	unnamed (+52), unnamed (+72150)	1.161825339	1.277656753
SNRPD3	unnamed (+7)	0.970706047	1.278194595
PPCDC	unnamed (-8)	1.347796768	1.278315659
NPM1	unnamed (-66), unnamed (+880)	0.824925751	1.278431082
TYW1	unnamed (+74)	1.052473378	1.278796712
PSME3	unnamed (-265)	0.66685942	1.279782653
U2AF2	unnamed (-53)	1.02594845	1.280109068
CHAC2	unnamed (+66)	0.915144628	1.280449711
CHTOP	unnamed (+112)	#N/A	1.281131938
SRF	unnamed (-151)	1.281756717	1.281296065
RTN4RL2	unnamed (+32705)	#N/A	1.281904224
FGF7	unnamed (+197663)	#N/A	1.282851518
LAMTOR1	unnamed (+246)	#N/A	1.283175385
CCNT1	unnamed (+116)	#N/A	1.283219641
PRR14L	unnamed (-3335)	#N/A	1.283529663
MAN2A2	unnamed (-17960)	0.941369151	1.284057716
RNH1	unnamed (-12062), unnamed (-73)	0.960978174	1.284484876
NARFL	unnamed (-134)	#N/A	1.286989063
KIN	unnamed (-60)	0.855158435	1.287218575
MYL5	unnamed (-3538)	#N/A	1.287735405
KCTD15	unnamed (-18663)	1.002508185	1.288222562
ZBTB20	unnamed (-763032)	1.218857388	1.28823428
TBCC	unnamed (-37333)	0.836275669	1.28877466
ZDHHC7	unnamed (-33)	1.282883803	1.289094869
VMP1	unnamed (-22)	#N/A	1.28916103
U2AF1L4	unnamed (-2904), unnamed (+178)	#N/A	1.289407349
VMAC	unnamed (-1019)	#N/A	1.289435089
MLL3	unnamed (-28374)	0.895896301	1.291571674
ALDH4A1	unnamed (+82)	#N/A	1.291658938
TRAPPC9	unnamed (-5936)	0.816630887	1.291711914
E4F1	unnamed (+177)	0.907621781	1.291917179
SMG5	unnamed (+199)	1.130804932	1.29215237
HSPD1	unnamed (-119)	1.055001859	1.293125466
NME6	unnamed (-168)	0.969358739	1.293610592
ERGIC2	unnamed (+56)	0.635159602	1.293613144
SHF	unnamed (+2458)	#N/A	1.294584637
NEK11	unnamed (-202)	#N/A	1.294858201
NELF	unnamed (-32)	1.220604505	1.295085314
DHDDS	unnamed (-25)	0.957968359	1.295170326
MUTYH	unnamed (+121)	1.187179303	1.295227404
RALBP1	unnamed (-200)	1.127620568	1.295791786
AFF1	unnamed (+72107), unnamed (+285639)	0.855361752	1.29659046
M6PR	unnamed (-460)	1.004844392	1.299045351
C12orf65	unnamed (-356)	0.828904449	1.299046585
MED1	unnamed (+49)	1.146965276	1.299131114
SPA17	unnamed (+13)	0.793849964	1.300166771
SNRPC	unnamed (+289)	0.883934649	1.30107936

ARHGEF17	unnamed (-1344)	#N/A	1.301196868
RHBDD2	unnamed (+197)	1.597272424	1.301597678
SRSF10	unnamed (+215)	0.682180298	1.301783841
RBFA	unnamed (+355)	#N/A	1.302975856
PSMD9	unnamed (+135)	1.053799419	1.303238049
ZNF548	unnamed (-181)	0.728121437	1.303776451
ANKHD1-EIF4EBP3	unnamed (+733)	#N/A	1.303943714
AMDHD2	unnamed (-13)	#N/A	1.304614006
TPST1	unnamed (+288276)	#N/A	1.305681778
LAP3	unnamed (-220)	1.500804582	1.305744154
TMEM44	unnamed (+49502)	#N/A	1.30621196
NKIRAS2	unnamed (-2449)	1.054088511	1.306433899
EHD1	unnamed (-169)	1.154353952	1.30774451
ANAPC2	unnamed (-13)	#N/A	1.308867814
POLR3B	unnamed (-54923), unnamed (+100)	1.056611172	1.309477085
FOXL2	unnamed (-382404)	#N/A	1.309656952
PPP1R12A	unnamed (+347)	0.969348205	1.310100502
LRRC8A	unnamed (-66)	0.747117539	1.311832137
EMG1	unnamed (+12)	0.830296154	1.312568131
GTPBP1	unnamed (-4651), unnamed (-354)	1.428489377	1.312908873
HSD17B10	unnamed (-249698)	0.930387466	1.313241366
SIPA1L3	unnamed (-372)	#N/A	1.31336757
C6orf120	unnamed (-82)	1.191615434	1.313773483
IMMP1L	unnamed (-124)	0.4935279	1.313939555
PRDX2	unnamed (-69), unnamed (+8211)	0.954582836	1.31482657
DEGS1	unnamed (-190563), unnamed (+163)	0.880846025	1.314925046
CNPY2	unnamed (+267)	0.599888489	1.315193115
CELF1	unnamed (-64318)	0.86566717	1.315226673
AGRN	unnamed (+43961), unnamed (+49303)	0.915921158	1.315869375
UROS	unnamed (-226)	0.750069172	1.316027981
GIPR	unnamed (+13484)	#N/A	1.316091797
SFT2D3	unnamed (+130)	0.786263211	1.317790639
HIST1H4E	unnamed (+627)	#N/A	1.318110492
CHMP4B	unnamed (-208)	0.998069408	1.318182676
ZNF561	unnamed (-79)	0.722092486	1.319650049
RAVER1	unnamed (+418)	0.998978927	1.32063984
SESN1	unnamed (-882)	1.541384219	1.320724793
UBE2D2	unnamed (-96)	0.941760539	1.320765548
SPR	unnamed (-24827)	0.985124347	1.320791464
SNX21	unnamed (-102)	#N/A	1.321322846
ZNF397	unnamed (-199846), unnamed (-51)	#N/A	1.322174003
GLUD1	unnamed (-599)	1.082544904	1.322327975
ATG5	unnamed (+41)	0.903182794	1.32261199
ZFP91	unnamed (-765)	1.277288214	1.323043053
MAP4K1	unnamed (-1061)	#N/A	1.323486476
ADCK5	unnamed (-226)	#N/A	1.32360585
ANKRD54	unnamed (-4486), unnamed (-219)	0.856688595	1.324249529
TH1L	unnamed (-92322)	0.886613376	1.324859903
ZC3H10	unnamed (+10)	#N/A	1.3251323
PRPF4	unnamed (-90)	1.214992768	1.325476753

RPS14	unnamed (-162)	0.997158557	1.325809775
METTL1	unnamed (-316)	#N/A	1.326169633
SERPINI1	unnamed (+218)	0.853467112	1.32641409
LSMD1	unnamed (+349)	0.6281296	1.326641162
SLC25A10	unnamed (-8988)	#N/A	1.326672982
IDH3G	unnamed (+90)	0.989411611	1.327463068
RPL9	unnamed (-462)	0.975495217	1.329054171
SLC35F5	unnamed (+93)	0.720400156	1.329189758
AGBL3	unnamed (+72)	#N/A	1.330069102
GLCE	unnamed (-258)	1.389394202	1.331323461
ABL1	unnamed (+283)	1.006920776	1.331337888
AIMP2	unnamed (-347)	0.840971774	1.331449287
ZFP36L1	unnamed (-20544), unnamed (+2677)	1.881628789	1.332416655
SUV39H1	unnamed (+8)	0.832961885	1.332640385
SAR1A	unnamed (-144)	1.314534214	1.333348168
PKMYT1	unnamed (-3272)	1.312015797	1.33399879
PSMG1	unnamed (+104)	0.976294602	1.334675382
PEX14	unnamed (-2306)	1.152060814	1.334876863
ARIH1	unnamed (-290)	0.975939381	1.334898831
THAP11	unnamed (-8323), unnamed (-405)	0.970683237	1.335091187
PFKFB2	unnamed (-2679), unnamed (-315)	1.278480904	1.336359766
DNAJC7	unnamed (+77)	0.53023241	1.336861542
PPP1R15B	unnamed (-178)	1.104709924	1.337238443
EBI3	unnamed (+17539)	#N/A	1.337360027
DBF4B	unnamed (-18725)	#N/A	1.337664783
ETFB	unnamed (-11745)	0.801607371	1.337939225
PTRHD1	unnamed (+252)	#N/A	1.338128144
CSNK1D	unnamed (-27)	0.70560267	1.338678873
MRPS17	unnamed (-14)	#N/A	1.33870603
NAB1	unnamed (-431)	0.627615999	1.338937318
TGIF2	unnamed (+32095)	1.029017297	1.339741529
RERE	unnamed (+64)	0.886742297	1.340177135
KIAA0494	unnamed (-107)	1.127555584	1.341383755
ZNF295	unnamed (+139)	1.097137091	1.341720917
CSNK1G2	unnamed (-406)	0.991848622	1.342556173
ATP6V0C	unnamed (+65)	1.099023901	1.342591978
DHX34	unnamed (-131)	#N/A	1.342783645
HTT	unnamed (+677)	#N/A	1.342980511
LTBP4	unnamed (+65728)	#N/A	1.344219534
CDKN2D	unnamed (-198)	#N/A	1.344748868
DBP	unnamed (-1072)	#N/A	1.345455595
ACBD6	unnamed (+88)	0.891370195	1.345792317
C8orf55	unnamed (-127)	1.290396093	1.346196485
TTC3	unnamed (-139)	0.518271176	1.346360591
SLC25A11	unnamed (-140)	0.789751536	1.346648575
MPG	unnamed (+18969)	1.230113596	1.34665306
SMARCD2	unnamed (-6096), unnamed (+62)	1.11678822	1.347036997
TONSL	unnamed (-12525)	#N/A	1.347234484
INTS12	unnamed (-65)	0.868050777	1.347470288
DOT1L	unnamed (-329)	1.563714903	1.347548669
RPL27	unnamed (+454)	0.981643005	1.350093256

EIF4A2	unnamed (+211)	1.022907744	1.350390024
LIN37	unnamed (-3104), unnamed (-22)	1.401264928	1.351145432
HDAC5	unnamed (-254)	#N/A	1.351369067
ZNF146	unnamed (+243)	0.762443896	1.351550683
ANKRD49	unnamed (-152)	1.101180247	1.351601766
VEGFA	unnamed (+304519)	0.854087562	1.351758291
IPPK	unnamed (-111)	1.550268131	1.351883655
SSSCA1	unnamed (+14)	#N/A	1.353118968
ABCF1	unnamed (-39)	1.0251098	1.353520734
PRCP	unnamed (-1081)	1.153812139	1.353977664
ZNF276	unnamed (+719)	#N/A	1.354110885
WDR77	unnamed (-214)	0.813006064	1.354266528
C17orf76-AS1	unnamed (-102)	#N/A	1.35557513
PLEKHA8	unnamed (+106300)	0.994594829	1.355954165
BNIP1	unnamed (+56), unnamed (+557)	0.810225854	1.356060051
DCUN1D5	unnamed (-31)	1.004618031	1.356285918
IRF2BPL	unnamed (-69532)	#N/A	1.356399779
IFT52	unnamed (+66793)	0.9032294	1.356838342
C10orf26	unnamed (-212)	1.177243796	1.356930088
ABCC5	unnamed (-107)	0.575758399	1.356937943
DNAJA2	unnamed (-64379)	1.030423313	1.357366391
UBC	unnamed (-25666), unnamed (+258), unnamed (+885), unnamed (+4281)	1.124899484	1.358055881
DDX58	unnamed (-24658)	5.329119919	1.359019736
POP4	unnamed (-1)	0.93595495	1.359401438
EXOSC6	unnamed (-158)	0.922978277	1.360450234
ZNF296	unnamed (-2821)	#N/A	1.360789728
ZNFX1	unnamed (-509)	1.792263988	1.361395078
LENG9	unnamed (-1076)	#N/A	1.362325718
STX5	unnamed (+507)	#N/A	1.362686594
COPS5	unnamed (-1954), unnamed (+33728)	0.991527395	1.364334175
ZNRD1	unnamed (-96)	0.783878705	1.364819238
LRRC16A	unnamed (-480)	0.760094442	1.365687651
ZFAND2A	unnamed (-151), unnamed (+21852)	1.935364037	1.366099871
ZNF566	unnamed (+22)	#N/A	1.367043082
C19orf57	unnamed (+25800)	#N/A	1.367281767
EIF4B	unnamed (+472)	0.681132428	1.367727886
ICMT	unnamed (-67)	0.766415308	1.368121626
TSTA3	unnamed (-18357)	0.580601346	1.368589238
CETN2	unnamed (-265)	0.881532142	1.369387529
ZNF155	unnamed (+149)	#N/A	1.369842111
HSPA5	unnamed (-197)	1.358692408	1.369961426
ANG	unnamed (-4693)	#N/A	1.371552577
EEF1G	unnamed (-190)	1.022638611	1.371921109
SLC43A1	unnamed (+21314)	1.179587919	1.372373109
CRKL	unnamed (-129)	1.593205542	1.3728242
ZNF121	unnamed (+27)	#N/A	1.373248058
RPS26	unnamed (-55)	0.9856257	1.373494989
CCDC59	unnamed (-124)	0.651876201	1.373630761
RPS28	unnamed (-244)	1.051677113	1.375584658
ADCK4	unnamed (-199)	#N/A	1.376099388

UNC45A	unnamed (-2401)	1.135373635	1.376756709
CYB5D1	unnamed (-241)	0.826027838	1.377456694
MINOS1	unnamed (-104)	#N/A	1.377626778
UXT	unnamed (+286)	0.802635138	1.378006318
WFIKKN1	unnamed (+5643)	#N/A	1.378059186
CYP20A1	unnamed (+672)	1.075909199	1.378130486
NUMA1	unnamed (-221)	0.752851712	1.378410342
GTPBP5	unnamed (-63)	0.920166176	1.378752933
UBQLN4	unnamed (-806)	1.355179073	1.378838318
PPP2R1A	unnamed (+111)	1.139206334	1.379030761
SNX11	unnamed (-49)	1.018103064	1.379099534
PFDN4	unnamed (-11)	0.545950916	1.379575874
TXNDC15	unnamed (-27967), unnamed (+506)	0.882929851	1.380237693
DNMT1	unnamed (+2)	1.045381418	1.382272406
SUGP2	unnamed (-272)	0.791084494	1.383332073
ATG4A	unnamed (-221)	0.616452027	1.383571064
TMEM147	unnamed (-34)	0.868778092	1.384375238
SLC30A7	unnamed (+91)	1.544726612	1.384851964
ZFPL1	unnamed (-65)	#N/A	1.385135218
DDX56	unnamed (+429)	1.097705586	1.385724213
DPP9	unnamed (-154)	#N/A	1.386438606
SSBP4	unnamed (+604)	0.780131544	1.386862274
DNAJB9	unnamed (-54)	0.687494231	1.387197249
XPOT	unnamed (-181788)	1.204319934	1.387436615
ZNF181	unnamed (-350)	#N/A	1.387984766
PDCD7	unnamed (+817)	1.503861032	1.389466812
C6orf162	unnamed (-98)	#N/A	1.389920159
PTGES3	unnamed (-364)	1.05233826	1.39011658
GPR108	unnamed (-54)	0.907393064	1.390322464
NFATC3	unnamed (-470)	0.907004109	1.390620678
MSL2	unnamed (-1014), unnamed (-458)	0.974669833	1.391141841
NUP43	unnamed (-111)	1.008640517	1.392094552
RNF44	unnamed (-357)	1.320357501	1.392470038
ZNF174	unnamed (-206)	#N/A	1.393044673
ETNK1	unnamed (-80593)	0.616618166	1.394401324
TRPM5	unnamed (+22586)	#N/A	1.394527431
RNF166	unnamed (-54)	#N/A	1.395484016
BTBD1	unnamed (+55592), unnamed (+81310)	0.473759562	1.396063059
FASTKD2	unnamed (+46)	0.909681605	1.396543549
BTN2A2	unnamed (+91)	#N/A	1.396552389
CACYBP	unnamed (-379)	0.642145234	1.396725984
HRK	unnamed (+61881)	1.675979799	1.396825085
CLTB	unnamed (-323)	1.054968171	1.397218304
PDE4DIP	unnamed (+62441)	1.603789869	1.397315152
STK38	unnamed (+48)	0.755966472	1.398212615
HSP90AB1	unnamed (-256), unnamed (+587)	1.100804145	1.398386476
PLEKHM3	unnamed (-26)	#N/A	1.398901959
KSR1	unnamed (-138927)	#N/A	1.399093893
TPM3	unnamed (+8782)	0.616518214	1.401942275
POM121	unnamed (-125)	#N/A	1.4026435
NOP16	unnamed (-165)	0.968794413	1.402651391

CES2	unnamed (+3)	1.150302389	1.402854703
TAF7	unnamed (+114)	0.935058909	1.4030804
ZNF76	unnamed (-244)	#N/A	1.403530198
CENPP	unnamed (+130)	#N/A	1.403828793
FONG	unnamed (+27)	#N/A	1.404340191
SMAD2	unnamed (+181840)	0.59894465	1.40479651
NUP133	unnamed (+131)	0.987070506	1.405116896
EPB41L4A	unnamed (+258432)	0.806065602	1.405434193
GNL1	unnamed (+647)	1.096171485	1.405482144
NMRAL1	unnamed (+214)	0.804726079	1.406658765
HS2ST1	unnamed (+807)	0.83978865	1.407476831
SNAP47	unnamed (+320)	0.961952943	1.408001595
ZBTB1	unnamed (-715), unnamed (+314)	1.03815782	1.408710936
CPNE7	unnamed (-10042)	#N/A	1.40954038
SRRD	unnamed (+124)	0.972780796	1.409656917
FAF1	unnamed (-151)	0.893976426	1.411442717
C11orf2	unnamed (-89)	0.847734394	1.412000837
NOP2	unnamed (+24)	0.650132054	1.412055415
RAB4B	unnamed (-581)	#N/A	1.412121541
MLL4	unnamed (-966)	1.285179424	1.412189185
PPOX	unnamed (-67)	0.931285767	1.412326435
BFAR	unnamed (-118)	1.124433211	1.412473485
SAR1B	unnamed (-1), unnamed (+105079)	0.786920423	1.412937719
PPAN-P2RY11	unnamed (+210)	#N/A	1.413123877
RPS19BP1	unnamed (-90)	0.771489468	1.413203984
SEC63	unnamed (+156)	0.882765671	1.413813126
ZFAT	unnamed (+355)	0.893962463	1.414765564
ZNF565	unnamed (-181)	#N/A	1.414864897
NAA20	unnamed (+35269)	0.907845994	1.416342198
TRIM38	unnamed (+29812)	#N/A	1.417096136
VPS4B	unnamed (+53)	1.083389035	1.417918147
EGFL7	unnamed (-34239), unnamed (-21134)	#N/A	1.420423556
XRCC2	unnamed (+211786)	1.152610779	1.421186244
NPRL3	unnamed (+18), unnamed (+41559)	0.857548481	1.422117139
DHPS	unnamed (+274)	0.61923339	1.422771619
MEPCE	unnamed (-486)	1.168526553	1.422894077
WDR62	unnamed (-439)	#N/A	1.422988205
RNASEH2A	unnamed (-4665)	0.909395079	1.422997604
FES	unnamed (+1795)	#N/A	1.423043986
SF3B5	unnamed (-23)	1.00085978	1.423524312
CTDP1	unnamed (-35592), unnamed (-287)	#N/A	1.423722492
STYXL1	unnamed (+137)	0.948968737	1.424072843
RNF138	unnamed (-729)	0.760066535	1.425242911
ZNF394	unnamed (-4926), unnamed (-4357), unnamed (+20)	0.938703814	1.427297956
AGPAT6	unnamed (-76)	0.855342112	1.427966732
C9orf156	unnamed (+215)	0.708979793	1.428159503
CDC20	unnamed (-407)	1.0159487	1.428206324
BRMS1	unnamed (0)	1.327273322	1.428886152
TFCP2	unnamed (+51)	0.857081653	1.428993197
WDR74	unnamed (-1586), unnamed (+429)	0.753076039	1.42912275

NDUFB1	unnamed (+7)	0.955164178	1.429254979
PTPN6	unnamed (-8557), unnamed (-2776), unnamed (-1611)	1.265069722	1.430049674
CCDC86	unnamed (+97)	0.686832938	1.43009792
GPBP1L1	unnamed (-497)	0.75970386	1.430598989
PTPLAD2	unnamed (+347461)	#N/A	1.431745825
LANCL2	unnamed (+737)	0.738363367	1.431957531
RHOT2	unnamed (-31428)	0.854794149	1.432200479
NFATC1	unnamed (+243883)	#N/A	1.432680476
ARID1A	unnamed (-75190), unnamed (-2798), unnamed (-420)	1.011588498	1.433671497
PTPLB	unnamed (-358)	0.68932233	1.433722024
ZNF414	unnamed (+141)	#N/A	1.434938932
CLP1	unnamed (-213)	1.265873134	1.435189797
GEMIN8	unnamed (+303)	0.57574609	1.435380708
ABCF2	unnamed (-105)	0.694927449	1.435563394
TMEM119	unnamed (-7884)	#N/A	1.43569068
MAGOHB	unnamed (-71)	0.810540126	1.436065849
CNP	unnamed (-122)	1.292515746	1.436649819
NUP37	unnamed (-1787)	1.053591092	1.438698163
NSMCE2	unnamed (-32)	1.044648208	1.438784838
DIAPH1	unnamed (-292)	0.562210871	1.440002504
LYRM7	unnamed (-80)	0.483598991	1.440273016
IGLL1	unnamed (+298284)	#N/A	1.440519098
GATAD2A	unnamed (-148)	0.940071352	1.440657459
ESYT1	unnamed (-220)	0.654168142	1.441609714
FEM1B	unnamed (-43)	0.684169506	1.442965832
B4GALNT2	unnamed (+59440)	#N/A	1.443283964
RHEBL1	unnamed (+9158)	2.273377687	1.443477356
LSG1	unnamed (+20)	1.155757399	1.443514516
COQ7	unnamed (+184), unnamed (+19142)	0.76067531	1.444109626
TECPR1	unnamed (+244)	#N/A	1.445039408
RNASEH2C	unnamed (+189)	0.809709479	1.445308824
RDH13	unnamed (-171)	0.976822703	1.445547311
SLC35A2	unnamed (+432)	1.455326368	1.447218598
TMEM141	unnamed (+540)	0.649209848	1.449192465
PRKAB2	unnamed (+270620)	0.956385654	1.449478935
ZC3H6	unnamed (+51)	#N/A	1.449847622
TOB2	unnamed (+153)	1.361423404	1.449893071
WWOX	unnamed (+382)	#N/A	1.450169944
HRAS	unnamed (+16667)	1.084294379	1.450498445
POLR1C	unnamed (-10)	0.773955048	1.450836735
PIK3R2	unnamed (-289), unnamed (+15020)	#N/A	1.451261257
TTC4	unnamed (-17)	0.998337994	1.452742712
GUK1	unnamed (-114)	1.188226401	1.453193383
IFNGR2	unnamed (+77546)	0.934391426	1.453368422
ZNF614	unnamed (-130)	#N/A	1.45390537
FTH1	unnamed (-43)	0.867275919	1.454358209
THRAP3	unnamed (-382)	0.788881472	1.454561384
H1FX	unnamed (+37714)	1.591327336	1.455886774
NUP188	unnamed (+14)	0.904401555	1.456288753
DIMT1	unnamed (-8755)	#N/A	1.457513593

PPP6C	unnamed (+105)	0.879092167	1.458036395
ABHD2	unnamed (+243)	1.079191975	1.458262269
WRNIP1	unnamed (-260)	1.018774696	1.459048902
SUPT16H	unnamed (+150)	0.640958457	1.459655792
GET4	unnamed (-166)	0.95015532	1.460126984
EZR	unnamed (-181838)	0.84917381	1.460735957
C18orf32	unnamed (-355)	0.688160672	1.461826077
IST1	unnamed (-175)	#N/A	1.461921309
SELT	unnamed (+100666)	0.893147662	1.461961497
RAB5C	unnamed (-47)	0.964978467	1.462226546
VPS45	unnamed (+297)	0.864756756	1.46337112
APOL3	unnamed (+36762)	#N/A	1.463602574
AHCTF1	unnamed (-631)	0.590815055	1.464430333
ZNF24	unnamed (+420)	1.112453676	1.464727589
BCAS1	unnamed (+11697)	#N/A	1.465049323
NFYC	unnamed (+40)	1.028550635	1.465893455
ZNF593	unnamed (-33)	1.01951406	1.468708416
CLIP1	unnamed (+781)	0.980396884	1.469816279
LUC7L	unnamed (+48021)	0.889595097	1.470174969
AES	unnamed (+27260)	0.874293065	1.471559821
NMNAT1	unnamed (-377)	1.510943779	1.471779196
C16orf80	unnamed (-123)	1.45089316	1.472574814
JOSD2	unnamed (-397)	#N/A	1.472652559
JMJD6	unnamed (+49)	0.501456266	1.473065606
PRKACA	unnamed (-137)	#N/A	1.474168056
ORMDL1	unnamed (+310)	0.851819298	1.474216654
ZNF169	unnamed (-92145)	#N/A	1.474559974
SRSF4	unnamed (+33)	0.93224361	1.475581305
RXRA	unnamed (-188405)	1.157468196	1.478258602
BRI3BP	unnamed (-4531)	1.569822353	1.478649456
TMBIM6	unnamed (-423)	0.655828814	1.479018586
CHMP1A	unnamed (-217)	1.157119401	1.479206858
NDST2	unnamed (-10)	#N/A	1.479424387
PTP4A2	unnamed (-191)	0.916386982	1.480904286
TPRN	unnamed (-4963)	#N/A	1.481866031
RNPS1	unnamed (-293)	0.788075491	1.482189217
CYHR1	unnamed (+8142)	#N/A	1.482883905
BRF2	unnamed (+24)	1.259137843	1.483163595
JARID2	unnamed (+2559)	1.592422103	1.483472056
BTG3	unnamed (-206568)	0.55822479	1.483534189
CDK5RAP1	unnamed (-48)	1.053510411	1.485381585
FKBP5	unnamed (+165893)	0.677356604	1.48643786
TMEM69	unnamed (-1048)	0.940169585	1.486802879
PCMTD1	unnamed (-170)	0.532506448	1.488161831
UBQLN1	unnamed (+97)	1.035678795	1.489256467
ARF3	unnamed (-301)	0.70640771	1.489825992
EXOSC4	unnamed (-10)	0.827170874	1.490583892
GEMIN7	unnamed (-9)	0.82539939	1.490758654
ZBTB6	unnamed (+207)	#N/A	1.49158294
PPP1R13L	unnamed (-1458)	1.204523337	1.492036247
PTDSS1	unnamed (-219)	1.127128752	1.492720326

RBBP6	unnamed (-277)	0.8305899	1.494531489
GPR146	unnamed (-64197), unnamed (-13246)	#N/A	1.494838952
CNOT4	unnamed (-372)	0.912978651	1.496379431
PLXNA1	unnamed (-5943)	0.807926204	1.496998725
ADI1	unnamed (+76)	0.944756186	1.49730928
ZBTB4	unnamed (-135)	1.050195686	1.497942157
ISG20L2	unnamed (-748)	1.110687714	1.498239269
C19orf40	unnamed (-82)	0.820605961	1.500227009
PCBP2	unnamed (+399)	0.929024866	1.500393326
C20orf72	unnamed (-188)	1.24387393	1.501232605
MPZL1	unnamed (-7176)	0.95408693	1.501524535
PRDX4	unnamed (-54)	1.005782782	1.501552728
CCRN4L	unnamed (-53)	1.084226372	1.501900949
BRP44	unnamed (+596)	0.625739457	1.50277253
DCAF7	unnamed (-140)	1.048244158	1.504513373
ZNF559	unnamed (+535)	1.054821191	1.504767061
B4GALT7	unnamed (-72)	0.787832204	1.505196213
AFF4	unnamed (+847)	1.379839511	1.50525347
C16orf42	unnamed (-80)	0.711711358	1.506868394
KIF1B	unnamed (-392)	0.928875115	1.50769586
WRAP73	unnamed (+49)	#N/A	1.508846811
PPFIA3	unnamed (-455)	#N/A	1.508951457
AP2B1	unnamed (+121)	0.707516796	1.512703565
OPA3	unnamed (+18)	1.226318734	1.512829302
AIP	unnamed (+136)	0.927549193	1.512945233
ARPC1B	unnamed (-94)	1.020172221	1.512954777
AKR7A2	unnamed (+43)	0.838916745	1.513429879
LYRM2	unnamed (-207)	0.549195668	1.513838663
PAFAH1B3	unnamed (+111)	0.809886347	1.513988802
BAIAP2	unnamed (+66744)	#N/A	1.514040771
PDE6D	unnamed (+66658)	0.909977354	1.514358563
TBL3	unnamed (-63)	0.64696499	1.514861001
POLR2H	unnamed (+51)	0.999215023	1.516564533
SLC25A26	unnamed (+152024)	0.642550209	1.516814177
REEP3	unnamed (+520033)	0.870978533	1.517028057
WDR59	unnamed (-178)	#N/A	1.517207683
RPS3A	unnamed (+232)	0.90394065	1.517659228
SIAH2	unnamed (+59531)	1.040320534	1.518200142
PQLC1	unnamed (+7)	1.110925081	1.518220148
FDXR	unnamed (-428)	#N/A	1.518971506
RAP1GDS1	unnamed (-414)	0.400253781	1.520884663
RACGAP1	unnamed (-121)	0.898364144	1.521635866
SLC25A25	unnamed (+49612)	#N/A	1.52245809
TRAF7	unnamed (-333)	0.817170321	1.523559412
HIST2H2AC	unnamed (-1413)	#N/A	1.525057978
BCDIN3D	unnamed (-6)	0.780380467	1.526550964
JUNB	unnamed (-1405), unnamed (+2173)	1.252302544	1.526631426
SUV420H1	unnamed (-532)	0.847283423	1.527557219
RHBDD3	unnamed (-421)	1.355150135	1.528022755
MTX1	unnamed (+18408)	#N/A	1.528257824
MYO9B	unnamed (-247)	#N/A	1.52984582

CHD8	unnamed (-24803)	1.090693731	1.529961544
MRPL49	unnamed (-4432), unnamed (-7)	1.224893389	1.530187017
CD58	unnamed (+152494)	0.876565736	1.530372634
KATNB1	unnamed (-545)	0.873294811	1.531263264
MAGT1	unnamed (-4000)	1.06066434	1.532684966
GATC	unnamed (-13)	0.851084727	1.534807189
HM13	unnamed (-65), unnamed (+58466)	0.814744264	1.535221206
HMOX2	unnamed (-1659)	0.927736615	1.536762516
NOTCH2NL	unnamed (-81)	0.778998937	1.53679356
KIAA0415	unnamed (-240)	1.030293959	1.53760602
ANAPC5	unnamed (-86)	0.980559272	1.537724336
PSME2	unnamed (+5218)	1.249546605	1.541315135
DUS3L	unnamed (+293)	1.120250332	1.542912599
FKBPL	unnamed (-4)	0.95773209	1.543344173
ZNF266	unnamed (-7)	1.31075404	1.54431453
ORC2	unnamed (+279)	1.008326124	1.546081176
TXNDC17	unnamed (-57)	0.993617774	1.547594627
ITSN1	unnamed (-355)	0.98466307	1.550267524
RBM42	unnamed (-62)	0.99437232	1.553562325
TIPARP	unnamed (-2367)	1.445116404	1.553831179
XXYLT1	unnamed (-564)	#N/A	1.55562268
THOC6	unnamed (+179)	0.965126556	1.555872014
TFB1M	unnamed (-49)	0.924518659	1.555931851
RPS6KB2	unnamed (-77)	#N/A	1.557135985
ABT1	unnamed (-132)	1.311046579	1.559581516
SF3A1	unnamed (+32)	1.120330436	1.560692369
PRR7	unnamed (+6354)	0.76842892	1.561464437
EMD	unnamed (-145)	0.810710828	1.562548946
CSPP1	unnamed (-87)	0.64206322	1.564178419
MAD1L1	unnamed (-99)	1.159038596	1.564909609
GTF2A1	unnamed (-407), unnamed (+1351)	1.04415833	1.566027871
ACYP2	unnamed (+268)	0.980493954	1.567383126
TOR1A	unnamed (+8)	0.922286503	1.568413597
PMVK	unnamed (+37)	1.203337021	1.569727244
TAGAP	unnamed (+45006)	#N/A	1.570015591
MRPS14	unnamed (-115)	0.680476443	1.571389886
U2AF1	unnamed (-220)	0.844427529	1.57173621
KHSRP	unnamed (-508)	1.202053826	1.571905456
SOX7	unnamed (-109463)	#N/A	1.575259767
C22orf32	unnamed (+78)	0.724981089	1.576382004
PURA	unnamed (-6467), unnamed (-82)	0.870139899	1.576620255
CEBPE	unnamed (-1386)	#N/A	1.578613417
CCM2	unnamed (-294)	1.729594745	1.578748668
MECR	unnamed (-84)	0.685484417	1.579240611
PPM1H	unnamed (+332171)	1.912343336	1.580741713
HMGN5	unnamed (+5)	0.722752657	1.581324801
IQCE	unnamed (-3336)	0.986584546	1.584166784
IRF2BP2	unnamed (-1083)	0.997220729	1.584661252
LRFN3	unnamed (-5353)	#N/A	1.585127116
SLC39A6	unnamed (+61790), unnamed (+156676)	0.262305952	1.585489995
POR	unnamed (-30)	1.265310357	1.58610309

LZIC	unnamed (-269)	0.997879192	1.588069218
SF3A3	unnamed (-88)	1.376759351	1.588741101
DRG1	unnamed (+7)	1.047182323	1.589029216
PEX10	unnamed (-20)	#N/A	1.58916053
B3GALNT2	unnamed (-181)	0.776520652	1.589929495
AGPAT3	unnamed (+73998)	1.077325688	1.590326801
ZNF771	unnamed (-6862)	#N/A	1.591100579
UBE2I	unnamed (-479)	0.906748006	1.593842981
RGL2	unnamed (-185)	0.814351384	1.59399762
LSM14A	unnamed (-296)	0.981989629	1.594654314
IGFLR1	unnamed (-2638)	#N/A	1.59734203
TET2	unnamed (+101)	#N/A	1.597695171
TSHR	unnamed (+264074)	#N/A	1.598052424
TMEM102	unnamed (-120)	#N/A	1.598926812
SH3GL1	unnamed (-1954)	1.649986314	1.601043004
SRCAP	unnamed (-48539), unnamed (-926)	#N/A	1.602787543
THAP7	unnamed (+5)	0.792762576	1.604453831
SYNGR2	unnamed (-100)	0.856280925	1.607158569
MKNK2	unnamed (-250)	1.339867597	1.612484092
TMEM237	unnamed (+776)	#N/A	1.613786439
HES4	unnamed (-761)	1.768672884	1.614431748
PLAA	unnamed (-11), unnamed (+54581)	1.267536716	1.61546742
NDUFS7	unnamed (-60)	0.881041674	1.615655621
CCDC124	unnamed (-393)	0.674925887	1.618124803
ETF1	unnamed (-147)	0.721944222	1.61860839
C6orf89	unnamed (-33)	0.925085277	1.618675497
STK19	unnamed (+474)	0.863945628	1.621237178
LARS	unnamed (-14)	0.670139807	1.623156663
HSPA4	unnamed (-235)	0.777450748	1.623407797
FANCI	unnamed (-206)	0.99116134	1.623465227
SAE1	unnamed (+95298)	1.284288547	1.623846435
TRIAP1	unnamed (-13)	1.011397139	1.624836175
RAB1B	unnamed (-713)	0.666694544	1.625004433
MTCH2	unnamed (+62)	0.832047362	1.625120245
STK38L	unnamed (-8)	2.832799346	1.625336119
KIAA0319L	unnamed (-560)	1.00850976	1.625726432
WDR19	unnamed (-59)	0.851307925	1.626199897
MDH1B	unnamed (-108)	#N/A	1.626812269
AKT1S1	unnamed (+202)	0.882854701	1.627000473
FBXW2	unnamed (+98)	0.872604501	1.627988976
NUP62	unnamed (-307)	0.93896279	1.629564844
RPL35	unnamed (-193), unnamed (+8892)	0.959525292	1.63245627
ERH	unnamed (-194)	0.875223498	1.633365924
PHF15	unnamed (-1066), unnamed (+1656)	1.041643896	1.634389166
TRIM27	unnamed (+27679)	0.944159572	1.635341435
CNST	unnamed (-87)	0.969122494	1.636199538
HIST1H3D	unnamed (+2235)	#N/A	1.641284549
C20orf43	unnamed (+29)	0.983855101	1.641359893
C20orf3	unnamed (+39)	0.925120368	1.645854567
HEXDC	unnamed (+385)	#N/A	1.646037676
AP4M1	unnamed (-811)	#N/A	1.648054553

TIMM44	unnamed (+169)	0.767248286	1.648516253
NDUFB2	unnamed (-87)	0.723435298	1.64970055
AZIN1	unnamed (+225), unnamed (+56511)	1.166676181	1.650006681
FLNA	unnamed (-4446)	1.448166383	1.6501881
FBLN2	unnamed (+101496)	#N/A	1.653277
ERCC1	unnamed (-20006)	0.486157233	1.654277011
ENAH	unnamed (+178006)	0.390960441	1.655149079
CTU2	unnamed (-8)	#N/A	1.657118103
ZNF696	unnamed (-12486)	#N/A	1.658864452
CD164	unnamed (-2)	0.924846193	1.661882231
ERP29	unnamed (-111)	1.217772077	1.663886359
TUBB4B	unnamed (-4667)	#N/A	1.664540147
ARF1	unnamed (+20715)	1.139588631	1.665245832
NCL	unnamed (+547)	0.572356112	1.666860491
EEF1A1	unnamed (+416)	0.971892882	1.671278343
HBP1	unnamed (+927)	0.941265073	1.672545832
NPTN	unnamed (-242)	1.450636947	1.673374868
MRPL55	unnamed (-1), unnamed (+5937)	0.976669347	1.676349233
FAM63A	unnamed (+113)	0.708844896	1.677042498
PGD	unnamed (+118)	1.112471858	1.68010846
ITPRIPL2	unnamed (-27195)	0.820016899	1.684947693
FAU	unnamed (-37)	0.997253784	1.684960678
GNB2	unnamed (-73)	1.13588171	1.686179206
EDEM3	unnamed (-208)	1.066260663	1.687339081
RXRB	unnamed (-15)	0.640781449	1.687719358
ZNF687	unnamed (-656)	#N/A	1.688211818
ZFAND3	unnamed (-166)	0.920745099	1.688242196
SCAND1	unnamed (+41)	1.085664883	1.68858453
ZNF527	unnamed (+21)	#N/A	1.689943997
SMG9	unnamed (-288), unnamed (+349)	#N/A	1.691363496
SNRPD2	unnamed (+10457)	0.590182829	1.691424803
GBA	unnamed (+17755)	#N/A	1.693112566
HSPB1	unnamed (-135947), unnamed (-10776)	1.006745878	1.694745203
PPP2R5A	unnamed (-732)	1.430804966	1.696801495
GAA	unnamed (+6)	#N/A	1.697682366
PEF1	unnamed (+348)	1.185493219	1.698615707
TMEM170A	unnamed (-185)	0.878924437	1.701590776
NCAPH2	unnamed (+20)	#N/A	1.704395332
PDE4A	unnamed (-3846)	0.992569141	1.707952018
LSR	unnamed (-345)	#N/A	1.708610625
TRMT2A	unnamed (+12)	#N/A	1.708859524
DSTYK	unnamed (+5)	1.283723247	1.710566016
LRIG1	unnamed (+279536)	#N/A	1.7106484
SCYL1	unnamed (-102572), unnamed (-27637), unnamed (-25565)	1.188099993	1.71262445
TMEM179B	unnamed (-719), unnamed (-58)	0.741932409	1.715170175
BRD2	unnamed (-3893), unnamed (-1819), unnamed (-863)	0.93860673	1.718296298
SLC31A2	unnamed (+38)	#N/A	1.719686148
ZNRF2	unnamed (-149646)	#N/A	1.720588286
STT3A	unnamed (-291)	1.16030288	1.721312415
ADPRHL2	unnamed (-50)	1.017964419	1.721835367

H6PD	unnamed (+32570)	1.499712759	1.722648728
TXNL1	unnamed (+252)	1.112597171	1.722691355
PAK1	unnamed (-297)	0.969108801	1.722972012
TOR1AIP1	unnamed (+535)	0.749799946	1.723098956
ANP32E	unnamed (-39)	0.932438399	1.728398396
TBC1D22A	unnamed (-100)	0.763419465	1.732085199
TOE1	unnamed (+324)	1.091272725	1.732471519
SLC25A4	unnamed (-84)	1.220426363	1.736810372
PITPNM1	unnamed (-3252)	#N/A	1.736853893
MIF	unnamed (-122)	0.910121344	1.739039365
SBDS	unnamed (-1303)	#N/A	1.739322744
INO80C	unnamed (+197)	0.926658149	1.740272677
DMAP1	unnamed (-108)	0.652487996	1.74419847
RBM18	unnamed (-31)	0.829245869	1.748052617
MYL6	unnamed (+214)	0.809414276	1.75145814
HNRNPK	unnamed (+107)	1.00119959	1.75302498
GINS4	unnamed (-57)	0.929406059	1.753168941
ATP5C1	unnamed (-43)	0.729535555	1.754472895
PLEKHA4	unnamed (-4485), unnamed (-3686)	#N/A	1.757055698
BAK1	unnamed (+53)	#N/A	1.757715792
ACAD9	unnamed (+28)	0.892056179	1.759481469
TAB2	unnamed (-949), unnamed (-201)	0.867300123	1.759553466
DDX39B	unnamed (+525)	#N/A	1.765501428
HSBP1	unnamed (-10)	0.840172923	1.76589182
MBTPS1	unnamed (+132)	0.955533321	1.766298512
GPAA1	unnamed (-4012)	0.751986859	1.76750749
SLC16A3	unnamed (-15517)	0.823441554	1.770404286
MAP2K2	unnamed (-63)	1.063830503	1.772242884
SLC35C2	unnamed (+225)	0.973658646	1.772332196
SLC39A3	unnamed (-170)	0.848893424	1.77661794
SLC25A19	unnamed (-126)	0.941288061	1.779874033
FRMD8	unnamed (+35935), unnamed (+110870), unnamed (+112942)	1.286015385	1.783116702
VRK3	unnamed (+291)	0.774789533	1.785662924
NUDT19	unnamed (-87)	1.364247682	1.788373023
TXNDC12	unnamed (-58)	1.050039795	1.792387077
PA2G4	unnamed (-62)	0.695421902	1.793647125
PRADC1	unnamed (-213)	#N/A	1.796811544
ECI1	unnamed (+36)	#N/A	1.803593467
CD2BP2	unnamed (+106)	0.925936215	1.805117893
PTOV1	unnamed (-112)	0.804405298	1.805155447
KAT5	unnamed (+2)	0.925285407	1.810547322
RRP1	unnamed (-9)	1.044128027	1.811352318
GRB2	unnamed (+125)	0.806358863	1.811383315
SAP18	unnamed (-150)	0.90988374	1.813436113
SCAMP3	unnamed (-35)	1.118688684	1.813634887
AGXT2L2	unnamed (-2535)	0.995673348	1.817324918
AKIRIN1	unnamed (-302)	0.877646451	1.819116113
PCMT1	unnamed (-3032)	0.811815358	1.819270549
ATAD3A	unnamed (-503)	0.774519437	1.820907629
GPATCH3	unnamed (+157)	1.142975024	1.821062474

AATK	unnamed (+64181)	#N/A	1.821739614
ITPKC	unnamed (-19)	#N/A	1.823005928
FZD6	unnamed (+429)	1.446028703	1.823152035
MYBL2	unnamed (-9337)	1.186150042	1.823255602
ANKZF1	unnamed (-56)	#N/A	1.826431447
C19orf42	unnamed (-59)	0.776531832	1.8269952
KDM5B	unnamed (-151)	0.86812509	1.828868572
FUS	unnamed (+247)	0.707600647	1.829277383
TRA2A	unnamed (+100)	0.953370049	1.829462176
SNRNP70	unnamed (+30)	1.110061918	1.833583359
MRPL4	unnamed (+181)	0.847457531	1.834241142
BBC3	unnamed (+6645)	#N/A	1.837321932
MAPKBP1	unnamed (+22)	#N/A	1.838260726
RAB8A	unnamed (-14)	0.971884226	1.842974859
MRPL17	unnamed (+138)	0.930169234	1.845010627
SKIL	unnamed (-1988)	0.896402322	1.845190715
ARMC6	unnamed (+265)	1.285654586	1.847052136
EEF1D	unnamed (+115)	1.144129888	1.854762668
ACVR2A	unnamed (-769)	1.338129036	1.859325558
AP4B1	unnamed (-403)	1.170308416	1.863410074
CIITA	unnamed (+67263)	#N/A	1.866250254
CHIC2	unnamed (-66)	1.003442204	1.866341678
GTPBP3	unnamed (-2685)	0.898174664	1.869193258
PSMB2	unnamed (+235)	0.963430821	1.871341354
UQCRQ	unnamed (-33)	0.934101802	1.87182132
MAPKAPK2	unnamed (+966)	1.12168584	1.87232462
NETO2	unnamed (+105932)	0.937407163	1.879033345
BID	unnamed (-226908)	0.91787543	1.880161458
CDK10	unnamed (+13617)	0.696963683	1.880551173
RPL7A	unnamed (-2)	0.716754722	1.882271516
USP4	unnamed (-141)	0.948464967	1.887038614
C19orf68	unnamed (+33246)	#N/A	1.887641299
IMP3	unnamed (+80)	0.817901557	1.888777754
SSNA1	unnamed (+16)	0.837147675	1.889696648
THAP8	unnamed (+320)	#N/A	1.893122697
CD320	unnamed (+165)	0.998693193	1.894187787
DOHH	unnamed (-144)	#N/A	1.894589524
POLR2I	unnamed (-12824)	0.667135652	1.897354097
AP2S1	unnamed (-9339)	0.953770076	1.897440591
B2M	unnamed (-142), unnamed (+17378)	1.137655106	1.897783651
RFC3	unnamed (-275138), unnamed (+43)	1.076977973	1.899010034
WASF1	unnamed (-82)	0.798114753	1.901065702
CKS1B	unnamed (-319)	0.660992255	1.906325236
ZNF697	unnamed (-867)	2.410171283	1.907151969
RPS9	unnamed (+190)	0.640489092	1.910187964
MAGOH	unnamed (+32)	0.945156335	1.91295633
MAU2	unnamed (-111)	1.565732894	1.913281592
NR1H2	unnamed (+212)	0.86377576	1.914216746
KIAA2013	unnamed (+18067)	0.985269466	1.914216781
PSENEN	unnamed (-336)	1.092667588	1.917096108
C1orf43	unnamed (+103)	0.779820221	1.921893808

CAPN10	unnamed (-447)	#N/A	1.927343869
UBE2W	unnamed (-263)	0.712628625	1.928701855
IFT46	unnamed (+72)	1.294959396	1.934882308
CDC37	unnamed (+4)	1.097825129	1.937215048
CLPTM1	unnamed (-177)	0.926074326	1.939982157
THAP2	unnamed (+127)	#N/A	1.946461588
PSME1	unnamed (+5259)	0.96384403	1.947463071
RAB27A	unnamed (-19732)	0.818956017	1.948327358
HAUS8	unnamed (-1)	#N/A	1.95019932
TLE3	unnamed (-68)	1.011375974	1.95968261
WTAP	unnamed (-90)	0.782980595	1.961481927
SSR4	unnamed (+247)	1.118749513	1.965052369
COPE	unnamed (+227)	1.190990623	1.965678818
PSMD2	unnamed (-277)	1.297436627	1.965768
STX18	unnamed (-21)	1.228193727	1.967823401
SPTSSA	unnamed (-272)	#N/A	1.968815656
MCL1	unnamed (+583)	0.954601758	1.969350752
MED29	unnamed (-384)	1.494529126	1.979795043
EIF1AD	unnamed (-75)	0.875580015	1.98477734
SKP1	unnamed (-48974)	0.923094984	1.987369173
TMEM79	unnamed (-1649)	#N/A	1.989990567
IDH2	unnamed (-80)	0.90136557	1.996950109
ATMIN	unnamed (-125)	0.827822081	1.999576017
PHF8	unnamed (+901)	#N/A	2.004493573
TRAF4	unnamed (+536)	1.174941553	2.005418515
PIK3R1	unnamed (-605)	#N/A	2.005555059
CABIN1	unnamed (+145384)	1.042631224	2.007138425
MARCKS	unnamed (+1788)	0.960789871	2.009804606
ZNHIT2	unnamed (-4539), unnamed (-114)	#N/A	2.019295425
TRIM26	unnamed (+360)	1.82080721	2.020659534
EWSR1	unnamed (+337)	1.060119269	2.026752039
RAD23A	unnamed (+227)	0.777979508	2.027760569
TAP2	unnamed (-112)	1.570724283	2.030786301
MEF2BNB	unnamed (+245)	#N/A	2.03279205
MFSD8	unnamed (+751)	1.071391665	2.035862409
SH3BGRL	unnamed (+133)	0.666705463	2.03680393
MRPS12	unnamed (-394)	1.243618056	2.046260635
NDUFA11	unnamed (+191)	0.772588349	2.052650102
CEP89	unnamed (-197)	#N/A	2.061855976
UBE2H	unnamed (-51)	1.182496191	2.066926871
MLEC	unnamed (-447)	0.763131606	2.070718943
ADAMTS4	unnamed (-3380)	#N/A	2.07781709
PIGC	unnamed (-76)	1.105847827	2.079005983
ERVMER34-1	unnamed (+29208)	#N/A	2.081976683
MTCH1	unnamed (-103)	1.279867644	2.090151439
RPL10	unnamed (+34)	0.991856863	2.094445196
FADD	unnamed (+152)	1.059837944	2.096408657
HSPA8	unnamed (-521)	1.145037282	2.097804369
SUGP1	unnamed (-198)	0.970114597	2.101438574
DEM1	unnamed (-21)	#N/A	2.117371307
ANKRD11	unnamed (-281)	0.955222501	2.122459727

RAB35	unnamed (+565)	0.771696496	2.125028175
RAB35	unnamed (+565), unnamed (+29681)	0.771696496	2.125028175
RPS19	unnamed (-8)	1.015219549	2.131801392
CAMK2D	unnamed (-788)	#N/A	2.135925866
TIMM50	unnamed (+438)	0.925659023	2.138139172
RPS2	unnamed (-69)	0.537897563	2.140697361
ZNF473	unnamed (-698)	1.017503052	2.14335353
RCC1	unnamed (-11993), unnamed (-83)	0.711177157	2.145147227
SLC12A7	unnamed (-62466)	1.313643274	2.145470622
RPL18	unnamed (-188)	1.059230426	2.147853019
AHCY	unnamed (+84)	0.937433724	2.167651358
DUOX1	unnamed (+68723)	#N/A	2.170176135
SUPT5H	unnamed (+57)	1.087125951	2.173809642
HNRNPU	unnamed (+90)	0.754989301	2.176639366
RFXANK	unnamed (+147)	0.849293356	2.177060947
ENSA	unnamed (+260)	0.467389817	2.180985449
ADSS	unnamed (-72)	0.596853083	2.181633456
BCR	unnamed (+101659)	1.047887079	2.186743854
EFCAB2	unnamed (+1190)	0.495778836	2.188109294
ZNF576	unnamed (-182)	0.829364892	2.188602485
SOCS1	unnamed (+311721)	#N/A	2.195738857
DNAJC12	unnamed (-11354), unnamed (+73604)	1.537431568	2.205698029
MMP14	unnamed (+16593)	#N/A	2.206349104
SCLT1	unnamed (+121), unnamed (+282884)	#N/A	2.206389101
NDUFB7	unnamed (-77)	0.850078697	2.216907105
PSMB8	unnamed (-741)	#N/A	2.217708894
PNKP	unnamed (-2225)	0.575282902	2.226389361
C12orf44	unnamed (-297)	1.21564888	2.22803721
GRWD1	unnamed (-158)	0.616640091	2.24923128
MAPRE2	unnamed (+62940)	1.162182849	2.249550951
BCAP31	unnamed (-351)	0.975505118	2.255394444
SLC3A2	unnamed (+25151)	1.122504898	2.264216784
TMEM183B	unnamed (-104)	#N/A	2.286170247
ARHGAP35	unnamed (-58391)	#N/A	2.2882047
NAGK	unnamed (+159)	1.436563703	2.293213249
NIPAL3	unnamed (-273)	#N/A	2.296347397
CALML3	unnamed (+98331)	#N/A	2.320752536
HUWE1	unnamed (+2652)	0.833467894	2.327095883
TRIM25	unnamed (-69)	1.12083864	2.328454446
SDF2L1	unnamed (+99)	1.259637714	2.331531198
PFKFB3	unnamed (+52)	1.923254857	2.342817319
CARD8	unnamed (+45909)	0.726829196	2.345079559
CDC42	unnamed (+69)	1.204038839	2.362221969
FUNDC2	unnamed (+351)	1.041668465	2.363867595
UBAP2L	unnamed (-155)	0.922917353	2.364126442
PAF1	unnamed (+100)	1.163752723	2.374435968
RPL18A	unnamed (-398)	#N/A	2.376273965
C12orf62	unnamed (-71)	1.054930161	2.377495043
TXNIP	unnamed (+94)	1.350523947	2.385848016
STAT1	unnamed (-6149)	1.040016651	2.386774864
BANP	unnamed (-177)	0.957019946	2.432029115

PIH1D1	unnamed (-1635), unnamed (+47)	0.631088801	2.438139867
TMPRSS11E	unnamed (-97285)	#N/A	2.449048455
CENPN	unnamed (+805)	0.664247427	2.453672381
ALDH16A1	unnamed (-1405), unnamed (+277)	#N/A	2.45397714
EIF3K	unnamed (-18)	0.689214094	2.458376805
NUCKS1	unnamed (+1046)	0.681168339	2.473687014
ST6GALNAC2	unnamed (+28254)	#N/A	2.491041614
ZNF416	unnamed (-197)	1.280420727	2.491216699
STK25	unnamed (-246)	0.861162802	2.506155489
KPTN	unnamed (+151)	#N/A	2.509805917
PACS1	unnamed (+152491)	0.791081477	2.520960594
PTGES2	unnamed (+10383)	1.02129832	2.540538468
SLC39A7	unnamed (-156)	#N/A	2.557763396
SEPT2	unnamed (+178)	0.674374367	2.568968224
NANS	unnamed (-183)	1.259086524	2.571435502
PDE7A	unnamed (-605)	0.607095244	2.573707128
G6PD	unnamed (-574)	0.725979421	2.594102533
IRF9	unnamed (+735)	1.699728746	2.600780255
TFPT	unnamed (+155)	0.815280534	2.610145421
UFC1	unnamed (+293)	0.981584914	2.639780944
PTMA	unnamed (-95868), unnamed (+6081)	0.856772015	2.641171335
RPS11	unnamed (+68)	0.852830932	2.654780051
DDX49	unnamed (-512)	1.018400481	2.666326127
COPS8	unnamed (+417)	0.881237823	2.668109323
IRF6	unnamed (+21670)	#N/A	2.692001173
MNT	unnamed (+1146)	0.853635119	2.692218417
TMPRSS13	unnamed (-17633)	#N/A	2.717064314
PIP5K1P1	unnamed (+78431)	#N/A	2.719994589
KIAA0513	unnamed (+218013)	#N/A	2.722071185
PHF5A	unnamed (-251)	0.84728864	2.738882319
PRDX1	unnamed (+989)	1.075827417	2.757005265
HLA-E	unnamed (-144329)	1.133503632	2.76884915
HLA-C	unnamed (-94906)	1.188111404	2.770088711
SLC39A1	unnamed (+137), unnamed (+3900)	1.331863385	2.775593863
PRPF19	unnamed (-233)	0.993022617	2.785043675
RAD23B	unnamed (-257)	1.11744347	2.856124233
UBE2S	unnamed (-94)	0.651320903	2.88452381
TRAPPC3	unnamed (-41)	1.132898582	2.887200198
C9orf3	unnamed (-32959)	1.394967658	2.908442045
CBLL1	unnamed (-319)	1.121083345	2.937550173
ERF	unnamed (-13249)	1.109177885	2.965929873
IFI30	unnamed (-5543)	0.943156062	3.037746602
CD3EAP	unnamed (+303)	0.791498085	3.121251159
IFNGR1	unnamed (+434)	1.158701341	3.133398292
CSGALNACT2	unnamed (-32833)	1.073248293	3.180506237
SLC2A12	unnamed (-126068)	#N/A	3.182672648
CALM3	unnamed (-405)	0.913568806	3.184634046
TAF15	unnamed (-341)	0.91279518	3.186928482
MRPL42	unnamed (+251)	0.503114896	3.202869665
KLRG2	unnamed (+124152)	#N/A	3.234625022
ATP1A1	unnamed (+45426)	0.926398741	3.302282033

BPTF	unnamed (-334)	0.784165375	3.319161062
NGFR	unnamed (+72774), unnamed (+74992)	#N/A	3.379452895
S100A2	unnamed (-2895)	1.454259078	3.396488952
MR1	unnamed (+156627)	1.436793483	3.493944006
CCDC47	unnamed (+49)	0.540485809	3.5024719
USP46	unnamed (-63097)	0.813935851	3.517557364
BIRC7	unnamed (-178707)	#N/A	3.612963221
GCLC	unnamed (-2874), unnamed (-94)	1.183671341	3.673127925
NDUFA10	unnamed (-60)	1.106535274	3.69187719
DTYMK	unnamed (-341)	0.88320209	3.791687015
CAB39L	unnamed (-42363)	1.020157207	3.906965957
CKAP4	unnamed (-54800)	1.378699899	4.18777361
HNRNPA0	unnamed (-234)	1.270926182	4.237285013
PIK3R3	unnamed (-191)	0.521727163	4.261931103
ANK1	unnamed (+68025)	#N/A	4.339218659
HIST1H1A	unnamed (+25157)	#N/A	4.382749496
GLDC	unnamed (-35765)	#N/A	4.495179934
SEPW1	unnamed (+2)	1.332236756	4.566168785
ATF5	unnamed (+706)	1.030103723	4.573471061
POP1	unnamed (+62)	1.271689186	4.59199336
NMB	unnamed (+4062)	#N/A	4.744044724
SOD2	unnamed (-267)	1.33308831	5.334034639
RELB	unnamed (-158)	1.675425029	5.45186136
SLC12A2	unnamed (-787)	0.983240724	6.236767116
CHODL	unnamed (-425314)	#N/A	6.626341395
EPB41L2	unnamed (-126)	0.877262773	7.213376882
GPER	unnamed (+50280)	1.019785325	7.771619046
ALCAM	unnamed (+43)	#N/A	8.061643461
ST8SIA1	unnamed (-209835)	#N/A	9.46297885
IFI6	unnamed (-53379)	1.401050742	11.21594546
SGK1	unnamed (+139339)	1.182912762	14.21097432
GENE	BRCA1-common-binding-position relative to TSS	GSE22259	GSE30822
ABCC6P1	unnamed (+218080)	#N/A	#N/A
AK300656	unnamed (-165)	#N/A	#N/A
APITD1-CORT	unnamed (+271)	#N/A	#N/A
ARMC12	unnamed (-4924)	#N/A	#N/A
ASUN	unnamed (+39)	#N/A	#N/A
AX746903	unnamed (-173084)	#N/A	#N/A
AX747191	unnamed (-16198)	#N/A	#N/A
B7H6	unnamed (-116)	#N/A	#N/A
BIVM-ERCC5	unnamed (+38644)	#N/A	#N/A
C14orf176	unnamed (-6260)	#N/A	#N/A
C1QTNF9B-AS1	unnamed (+44)	#N/A	#N/A
CCDC64	unnamed (+97314)	#N/A	#N/A
CEP170P1	unnamed (+75351)	#N/A	#N/A
CHURC1-FNTB	unnamed (+65)	#N/A	#N/A
COL28A1	unnamed (-104942)	#N/A	#N/A
DCDC2B	unnamed (-3474)	#N/A	#N/A
DEFB132	unnamed (+32955)	#N/A	#N/A

DEGS2	unnamed (-33465)	#N/A	#N/A
FAM156B	unnamed (+22248), unnamed (+35940)	#N/A	#N/A
FAM187A	unnamed (-3229)	#N/A	#N/A
FAM210A	unnamed (-57)	#N/A	#N/A
GPR148	unnamed (+69132)	#N/A	#N/A
HSPE1-MOB4	unnamed (+38)	#N/A	#N/A
ISY1-RAB43	unnamed (-200)	#N/A	#N/A
KDM4B	unnamed (+79300)	#N/A	#N/A
KIAA1245	unnamed (+39319)	#N/A	#N/A
KLLN	unnamed (+116)	#N/A	#N/A
LCN10	unnamed (+14735)	#N/A	#N/A
LOC100506422	unnamed (+826214)	#N/A	#N/A
LOC150776	unnamed (+29)	#N/A	#N/A
LOC283922	unnamed (-102)	#N/A	#N/A
LRCH4	unnamed (-192)	#N/A	#N/A
MSH5	unnamed (-114)	#N/A	#N/A
NDUFC2-KCTD14	unnamed (+275)	#N/A	#N/A
NEDD8-MDP1	unnamed (+18889)	#N/A	#N/A
NOTO	unnamed (-24776)	#N/A	#N/A
NREP	unnamed (-183950)	#N/A	#N/A
NT5C1B-RDH14	unnamed (-777358)	#N/A	#N/A
OR2F2	unnamed (-33146)	#N/A	#N/A
OR4F16	unnamed (-92054)	#N/A	#N/A
OR5AU1	unnamed (+51931)	#N/A	#N/A
PARPBP	unnamed (+192)	#N/A	#N/A
PMF1-BGLAP	unnamed (+162)	#N/A	#N/A
PPIAL4B	unnamed (-329363)	#N/A	#N/A
PPIAL4F	unnamed (+417625)	#N/A	#N/A
RNF223	unnamed (+4881), unnamed (+10223)	#N/A	#N/A
RPL17-C18ORF32	unnamed (-835)	#N/A	#N/A
SEC1	unnamed (+415)	#N/A	#N/A
SKOR2	unnamed (-500121)	#N/A	#N/A
SLC7A5P2	unnamed (-79064)	#N/A	#N/A
SPATA25	unnamed (-3661)	#N/A	#N/A
SRL	unnamed (-12078)	#N/A	#N/A
TAS2R30	unnamed (-37453)	#N/A	#N/A
TCF24	unnamed (-66009), unnamed (+37030)	#N/A	#N/A
TMEM242	unnamed (+666)	#N/A	#N/A
TMEM82	unnamed (-878)	#N/A	#N/A
TRIM39-RPP21	unnamed (+15766)	#N/A	#N/A
TTLL9	unnamed (+9228)	#N/A	#N/A
URI1	unnamed (-96854)	#N/A	#N/A
ZBTB42	unnamed (+15528)	#N/A	#N/A
ZNF286B	unnamed (-131)	#N/A	#N/A
ZNF460	unnamed (-428)	#N/A	#N/A
ZNF487P	unnamed (+18651)	#N/A	#N/A
ZNF837	unnamed (+87)	#N/A	#N/A

ZSWIM4	unnamed (+51659)	#N/A	#N/A
SLC35E2B	unnamed (-194), unnamed (+33680)	1.426273681	#N/A
RNF165	unnamed (-160450)	1.146736954	#N/A
GOLGA8B	unnamed (-52372)	0.910937069	#N/A
SYPL2	unnamed (+17736)	0.793119645	#N/A
ZNF260	unnamed (-337)	0.740764212	#N/A

Supplementray Table 2. Expression correlation with BRCA1 frorm TCGA data		
1	BRCA1	1
2	C17orf53	0.507202373
3	TUBG1	0.481826735
4	DTL	0.474004952
5	VPS25	0.468765479
6	TOP2A	0.46160064
7	PSME3	0.45974753
8	TUBG2	0.458916057
9	NBR2	0.449332042
10	TIMELESS	0.447644644
11	CENPK	0.435977786
12	UHRF1	0.434323706
13	C16orf59	0.432517586
14	CDC25C	0.431206817
15	CCDC43	0.429186706
16	CDC6	0.428765508
17	CCDC56	0.427173795
18	GCN5L2	0.425435547
19	ZWINT	0.420987532
20	SPAG5	0.41855074
21	RDM1	0.417986587
22	WDR51A	0.41594053
23	KIAA0101	0.405385746
24	TRAIP	0.402986525
25	MLX	0.402135012
26	NUSAP1	0.393076183
27	MLF1IP	0.389929891
28	CENPM	0.389723383
29	WDR76	0.38931094
30	LIG1	0.38838626
31	ASF1B	0.387669588
32	CENPQ	0.386507361
33	RAD51	0.386464452
34	C17orf75	0.386002162
35	BUB1B	0.384827313
36	KIF4B	0.384444088
37	KNTC1	0.38226372
38	NEIL3	0.381176201

39	HMMR	0.379977597
40	UBE2T	0.378038631
41	SUZ12	0.3768383
42	PKMYT1	0.376136786
43	EFTUD2	0.37531983
44	OIP5	0.375195886
45	SPC25	0.375045354
46	C12orf48	0.373109436
47	TUBA1B	0.372996344
48	NEK2	0.372343732
49	KIF4A	0.37185128
50	COASY	0.371149209
51	ATAD5	0.367311229
52	CENPE	0.367015756
53	CCNB1	0.364602461
54	AARSD1	0.363478658
55	NMT1	0.363224806
56	ESCO2	0.362822581
57	NSF	0.361998445
58	POLQ	0.361249491
59	CDC2	0.357124689
60	RACGAP1	0.356439065
61	MCM6	0.353178908
62	HMGB2	0.351879529
63	PBK	0.351498646
64	MXD3	0.351096818
65	LOC283871	0.350734177
66	COX6A1	0.35035567
67	UBE2C	0.35003888
68	TMPO	0.349820023
69	NCAPH	0.349799095
70	SLC25A39	0.348189987
71	NPEPPS	0.34795749
72	KIF11	0.34761503
73	C16orf75	0.347604456
74	ERCC6L	0.347376833
75	GINS1	0.347312584
76	MCM2	0.346031856
77	ESPL1	0.344443724
78	DEPDC1B	0.343893377

79	H2AFZ	0.343615281
80	XRCC2	0.34357606
81	NKIRAS2	0.343045109
82	CENPI	0.342597505
83	CIT	0.341776973
84	DTYMK	0.341173042
85	DBF4B	0.34099503
86	TMEM97	0.33967961
87	PCNA	0.339536867
88	DHFR	0.338031718
89	TPX2	0.336367003
90	NFS1	0.336164068
91	BECN1	0.335885029
92	KIF15	0.335375964
93	PRIM1	0.334432891
94	PTTG3	0.333447456
95	DARS2	0.333304833
96	AURKA	0.332569766
97	CDC45L	0.332272413
98	MYOHD1	0.331099244
99	KIF20A	0.330919482
100	TRIP13	0.329946751
101	DSN1	0.329597009
102	MRM1	0.328969057
103	RNASEH2A	0.328560381
104	PTTG1	0.327116655
105	AURKB	0.326891576
106	E2F8	0.325981694
107	LSM12	0.325929817
108	RAD51L3	0.324761972
109	ACLY	0.324356309
110	RRM1	0.322049532
111	TROAP	0.321856648
112	MND1	0.321789077
113	LOC81691	0.32134116
114	BIRC5	0.321245455
115	PLK1	0.320339983
116	COQ5	0.320314256
117	PTTG2	0.319130228
118	MPHOSPH1	0.318639606

119	ZNHIT3	0.318608234
120	CASC5	0.317909106
121	RFC5	0.316890346
122	CENPF	0.316597771
123	PSMD11	0.315280959
124	ALKBH6	0.315058272
125	GSG2	0.315039807
126	ANKRD32	0.314937573
127	FEN1	0.314197715
128	CHTF18	0.314082135
129	LIG3	0.314035629
130	CEP55	0.31402539
131	CDCA5	0.313697656
132	NUF2	0.313603162
133	FAM134C	0.313513084
134	EXO1	0.313110801
135	ASPM	0.312561703
136	TMUB2	0.312547072
137	CENPH	0.31236476
138	PXMP2	0.311445267
139	LOC91431	0.311247309
140	HAGH	0.311242715
141	AATF	0.310408624
142	CKAP2L	0.310051828
143	E2F1	0.309205905
144	PRC1	0.309015078
145	TK1	0.308874946
146	ARHGAP11A	0.308771967
147	C12orf43	0.308428404
148	RRM2	0.307864153
149	KIFC1	0.30727655
150	KIF14	0.306616172
151	TYMS	0.305739426
152	FAM64A	0.305558032
153	CLSPN	0.304463481
154	IQGAP3	0.303544758
155	NTHL1	0.303150968
156	LRRC20	0.302654414
157	C17orf32	0.302209928
158	RAB11FIP4	0.301485863

159	UNG	0.301302059
160	ORC6L	0.300889568
161	MPP2	0.300336327
162	PLK4	0.300179991
163	C6orf129	0.299879456
164	BUB1	0.299699609
165	SFRS1	0.299692646
166	GINS2	0.299580458
167	HCN3	0.298634036
168	DKFZp762E1312	0.298496997
169	DEPDC4	0.298460868
170	C17orf65	0.297742124
171	DHRS13	0.297706559
172	C17orf79	0.297336181
173	MKI67	0.296746977
174	MCM8	0.296351991
175	DSCR6	0.296233708
176	LSM4	0.296154293
177	KIAA0286	0.295647678
178	TADA2L	0.295566821
179	CCNA2	0.295416523
180	C1orf135	0.295398617
181	DHX8	0.294828297
182	G6PC3	0.294745986
183	SHCBP1	0.294614467
184	MAD2L1	0.294435395
185	GLO1	0.294156827
186	POLE	0.293833252
187	MPHOSPH9	0.293758607
188	NT5C3L	0.293743941
189	PLEKHH3	0.292712359
190	MCM10	0.292262281
191	C9orf100	0.291969606
192	WDHD1	0.291927607
193	CDCA3	0.291714874
194	APITD1	0.291313118
195	CHAF1A	0.291247469
196	RACGAP1P	0.290917904
197	ACACA	0.29085841
198	PAFAH1B3	0.290838604

199	CKS1B	0.289390039
200	LAS1L	0.289358251
201	E2F7	0.288261058
202	DLG7	0.288154284
203	CCNE2	0.287820505
204	C13orf3	0.287397526
205	VDAC1	0.287343299
206	FOXM1	0.28687949
207	SGOL2	0.286256422
208	DNA2L	0.286169875
209	POLR2K	0.285657429
210	NCAPG2	0.285133322
211	TIMM17B	0.284692833
212	ZNF695	0.284399374
213	CDKN3	0.284352375
214	MELK	0.284230894
215	HELLS	0.284103358
216	NUDT1	0.283245215
217	KPNB1	0.282658741
218	FAM83D	0.282428808
219	SGOL1	0.282172226
220	MRPL24	0.282154472
221	PIGW	0.28202618
222	MRPL10	0.281844297
223	WDR62	0.281676116
224	PIGU	0.281675445
225	KIF18A	0.281152714
226	CACYBP	0.280530834
227	FLJ35767	0.279685625
228	DNAJC7	0.279622698
229	KIF23	0.278973826
230	DDX52	0.278609337
231	C1orf112	0.278508342
232	MYBL2	0.278476719
233	HSPA9	0.278432754
234	CDC23	0.277776202
235	GSS	0.277755589
236	RUNDC1	0.27730037
237	FLJ10241	0.277151097
238	MCM3	0.277147465

239	COPS6	0.277018676
240	NFKBIL2	0.276807917
241	LRRC45	0.276779801
242	C1orf35	0.276691506
243	UQCC	0.276442358
244	WDR67	0.276091626
245	C18orf24	0.275949859
246	ANKRD13B	0.275739374
247	KCTD13	0.275660053
248	RAD54B	0.275567099
249	NUP37	0.275255929
250	RBL1	0.274633348
251	ANAPC11	0.274533175
252	FAM54A	0.274202274
253	TUBA1C	0.274171333
254	NBR1	0.273999703
255	MCM7	0.273590206
256	ARL6IP1	0.273552323
257	GOSR2	0.273277692
258	CCNB2	0.272816524
259	ANLN	0.271981767
260	CDK2	0.271798006
261	ECT2	0.271350539
262	JUP	0.271309399
263	ATP6V0A1	0.271071724
264	ARS2	0.270885045
265	WDR79	0.27084566
266	ANAPC5	0.270669515
267	KIF22	0.270565372
268	BRI3BP	0.270496368
269	RAB5C	0.270439228
270	ATXN7L3	0.268941824
271	MASTL	0.268825623
272	WHSC1	0.268648977
273	EBP	0.268489371
274	CKAP5	0.268442696
275	KLHL11	0.267591121
276	15E1.2	0.267194567
277	STIL	0.266859895
278	POLE2	0.26659811

279	RAD51AP1	0.266495485
280	CENPL	0.266403289
281	SLC5A10	0.266305486
282	CEP250	0.266234072
283	HYPK	0.265975413
284	MRPL28	0.265786086
285	FANCI	0.265585769
286	CBX3	0.265116214
287	TSSC1	0.265039248
288	FAM104A	0.264904327
289	GMNN	0.264755603
290	SAE1	0.264661065
291	POLA2	0.264516905
292	PSMB3	0.264406724
293	GTSE1	0.263795897
294	SUDS3	0.263699598
295	CDC7	0.26348029
296	VAT1	0.263085072
297	FANCB	0.263077263
298	SC65	0.262251523
299	C16orf33	0.261908834
300	KIF2C	0.2615201
301	PTGES3	0.260805974
302	BLM	0.260699584
303	HISPPD2A	0.260594451
304	CENPA	0.260413118
305	RUVBL1	0.260089722
306	RIBC2	0.260030816
307	AP3M1	0.259580819
308	TMEM118	0.259019161
309	RHOT1	0.258351826
310	CEP152	0.258180227
311	YBX2	0.257924183
312	DNMT1	0.257455934
313	MTHFD1	0.257044483
314	CHCHD2	0.256940867
315	C1orf182	0.256871473
316	TIGD3	0.256836185
317	SLC25A10	0.25669645
318	C20orf52	0.256312521

319	ORAOV1	0.256245082
320	TTK	0.256219612
321	RFFL	0.255692283
322	KHDRBS1	0.255645297
323	MRPL17	0.255591284
324	RPAP1	0.255510142
325	POLG	0.255429999
326	C10orf35	0.255055938
327	UBE2S	0.255029895
328	NDRG3	0.25413542
329	NDUFB10	0.254089387
330	POLDIP2	0.254026354
331	DCC1	0.253761592
332	CSE1L	0.253588343
333	SNX11	0.25357137
334	C19orf40	0.253526195
335	ATAD2	0.253517009
336	RAD21	0.253257837
337	FANCG	0.253157952
338	C12orf60	0.252208807
339	MRPS12	0.252125267
340	NDC80	0.252019428
341	TUBGCP4	0.252009265
342	DEPDC1	0.251931734
343	LOC201725	0.251627308
344	RAN	0.251556062
345	MTX1	0.251247306
346	OBFC2B	0.251227044
347	LMNB1	0.250778385
348	POLR3K	0.250605444
349	CDK5	0.250212836
350	SFRS7	0.250037646
351	GTPBP5	0.24981806
352	GOT2	0.24951088
353	KIAA0841	0.249397098
354	PASK	0.249341753
355	TCF19	0.249330092
356	CCNF	0.249176959
357	TMPRSS4	0.249055074
358	RAD54L	0.249034431

359	AP2B1	0.248853549
360	ORC1L	0.248693836
361	UTP6	0.248514665
362	GGNBP2	0.248360475
363	CHCHD1	0.247800951
364	GOSR1	0.247728264
365	BTBD12	0.247527399
366	UBE3B	0.247482472
367	C10orf76	0.247080794
368	FAM77C	0.24663466
369	CENPO	0.246218601
370	AP3M2	0.246083636
371	C21orf45	0.245842903
372	SPINK5L3	0.245787769
373	C6orf125	0.245560003
374	RECQL4	0.245550078
375	SLC25A22	0.245495103
376	ELL3	0.245412417
377	KPNA2	0.24511683
378	IQCD	0.245070458
379	IARS2	0.244176388
380	C12orf32	0.244162448
381	FLJ10292	0.24414009
382	FADD	0.243835993
383	SLC46A1	0.243616949
384	UBTF	0.243357263
385	CHEK2	0.242885329
386	MATR3	0.242877637
387	RAPGEFL1	0.242676281
388	FAM57B	0.242648061
389	VPS33A	0.242369979
390	RBBP7	0.242279009
391	APOA1BP	0.242276087
392	CDK5RAP3	0.241679177
393	PPIL5	0.241586766
394	TACC3	0.241534933
395	ORMDL2	0.241524336
396	CDC25A	0.241322008
397	APBA2BP	0.241212087
398	USP30	0.241206113

399	MED24	0.240497922
400	B3GNTL1	0.24043086
401	CDK5R1	0.240386077
402	ZNF207	0.240244321
403	AIFM1	0.240059347
404	NDUFA2	0.239974307
405	DDX54	0.239967806
406	ACAD10	0.239788814
407	MRPL22	0.239787661
408	ANAPC7	0.239722044
409	TP53I13	0.239285962
410	GIT1	0.239174078
411	BLVRB	0.239089547
412	DMBX1	0.239003623
413	DNAJA3	0.23893224
414	TBCA	0.238712338
415	FLJ39660	0.238075135
416	OR2T11	0.23762828
417	CDCA8	0.236994178
418	Magmas	0.236164002
419	COX17	0.236127859
420	SFRS9	0.235528216
421	SFXN4	0.235384084
422	CCDC99	0.23536709
423	HSPD1	0.235330577
424	PPFIA1	0.235326087
425	C7orf44	0.235129218
426	MCRS1	0.235036969
427	DYNLL1	0.234971782
428	UNC119	0.234609043
429	NEK8	0.234442788
430	NMRAL1	0.234436422
431	NY-SAR-48	0.234240684
432	MRPS34	0.234166421
433	ERAL1	0.234094052
434	EARS2	0.234052684
435	RDH16	0.234003043
436	TALDO1	0.233858726
437	MRPL55	0.233747853
438	SMARCE1	0.233705338

439	RELL2	0.233654847
440	PACSIN1	0.233600907
441	DUSP13	0.233418996
442	EIF2S2	0.233291118
443	KLHDC9	0.233267328
444	CCDC21	0.233206638
445	NLE1	0.233184766
446	PSMD8	0.232285878
447	SMC1A	0.231814091
448	MGC4172	0.231766931
449	HN1L	0.231751913
450	CNP	0.231562714
451	TMEM145	0.231523966
452	NAP1L4	0.231449333
453	XPO1	0.231429822
454	C17orf42	0.231427222
455	GPSM2	0.231246841
456	C15orf42	0.230964415
457	PPIL1	0.230661746
458	C11orf48	0.230641166
459	CCDC138	0.230607182
460	MRPS16	0.230231444
461	PAQR4	0.230087868
462	ANKRD5	0.230075177
463	GTF2H3	0.229786346
464	RAD9A	0.229645855
465	TMEM106C	0.229423922
466	CSTL1	0.229298846
467	CTTN	0.229183134
468	KIAA1524	0.229167179
469	CCDC51	0.229112483
470	PIP4K2C	0.228653031
471	LARS2	0.228611382
472	KIAA0226	0.2284639
473	CHEK1	0.228319063
474	WIPF2	0.228267888
475	TRA16	0.227912602
476	LRDD	0.227685808
477	YWHAZ	0.227517498
478	ZNF587	0.227360092

479	WBSCR28	0.227259814
480	C1orf75	0.226360299
481	HEXIM2	0.226325159
482	NF1	0.226099688
483	C6orf107	0.225394378
484	BRIP1	0.225329646
485	ITGB3BP	0.225306225
486	TBCD	0.225197755
487	RFC4	0.224448636
488	RMI1	0.22423512
489	SMC4	0.223825709
490	KCNG3	0.223816604
491	PRIM2	0.223744761
492	BUB3	0.22368123
493	TMCO6	0.223661448
494	FAM128A	0.223632544
495	IPO9	0.223551615
496	TCEB2	0.223541173
497	NOX1	0.223482825
498	IGHMBP2	0.223262742
499	MYBL1	0.223125042
500	TSEN54	0.222990609
501	RPA3	0.222828304
502	MAPKAPK5	0.221993962
503	FAM128B	0.221945968
504	TMEM93	0.221939842
505	MSI2	0.221833549
506	PLA2G4F	0.221395893
507	CASC3	0.221148811
508	PNPO	0.221035575
509	CCDC44	0.220855154
510	C11orf82	0.220701866
511	CPSF6	0.220626246
512	PALB2	0.22048056
513	HMGN2	0.220437173
514	RNF135	0.220371902
515	DIAPH3	0.220172156
516	CDC20	0.219755223
517	GPRIN1	0.219632123
518	CAPN10	0.219509024

519	THOC3	0.219348199
520	MAPRE1	0.219075601
521	CKMT1A	0.218935444
522	CIB3	0.218862322
523	RPL27	0.218835922
524	SLC25A44	0.21866989
525	MRPS26	0.218588326
526	GCN1L1	0.217881546
527	C9orf140	0.217775135
528	C17orf37	0.217682822
529	FFAR2	0.217446538
530	EPN3	0.217441315
531	POLD1	0.217305646
532	DHX30	0.2171391
533	MRPL45	0.217102469
534	EP400	0.217042313
535	RUVBL2	0.217035238
536	COX6C	0.216883687
537	CDKN2D	0.216846285
538	NEURL	0.216760937
539	MRPS35	0.216589772
540	DHX58	0.216543387
541	SLC26A6	0.216526438
542	MNAT1	0.216410221
543	HS1BP3	0.216384304
544	TIMM50	0.216380996
545	TMEM68	0.216287943
546	AZIN1	0.216273982
547	SFRS14	0.216164615
548	NOLA2	0.216044967
549	PIGX	0.2157492
550	INTS2	0.215498192
551	ITPKA	0.215363117
552	NKAP	0.21535384
553	CHAF1B	0.215212867
554	DHX9	0.214943501
555	SYPL2	0.214878841
556	LOC200810	0.214858714
557	TCHP	0.214823125
558	NUP210	0.214799554

559	DDX46	0.214331326
560	GINS3	0.214282191
561	LIN7B	0.214123113
562	H2AFX	0.214068995
563	DHX35	0.214008658
564	DYNLRB1	0.213864061
565	PPP1R14D	0.213817073
566	FLJ14803	0.21380783
567	FAM111B	0.213764479
568	DIABLO	0.213668728
569	DCAKD	0.213508315
570	ACOT7	0.213474607
571	PAICS	0.213376525
572	NDUFS6	0.213277852
573	TOR2A	0.213275531
574	WBP11	0.213148399
575	HSPB9	0.213046538
576	C11orf17	0.213033318
577	SSX2	0.212994039
578	IFI35	0.212834797
579	TRAP1	0.212774277
580	PAXIP1	0.212513312
581	NOC4L	0.21246606
582	C1orf104	0.212454213
583	KRT33A	0.212310323
584	GTPBP3	0.212288748
585	NUP85	0.212261281
586	MSL-1	0.211899818
587	REEP4	0.211857571
588	CHCHD6	0.211825666
589	PSMC4	0.211816842
590	HSH2D	0.211684783
591	CEP72	0.211554544
592	CCND1	0.211378521
593	TBCE	0.211337486
594	MSH5	0.211304799
595	ADCK4	0.211270237
596	HDAC6	0.211220908
597	FRAG1	0.211195047
598	ZG16	0.211113974

599	CDAN1	0.210648569
600	PLEKHK1	0.210643113
601	C17orf61	0.210614637
602	MNS1	0.21051528
603	DHFRL1	0.210409155
604	CDCA2	0.210371049
605	SLC25A19	0.210347595
606	KIAA0406	0.210324241
607	TIMM23	0.210260375
608	TUBA1A	0.210203335
609	NARG1	0.210202942
610	SYNGR4	0.210182614
611	YTHDC2	0.210032283
612	C18orf56	0.209960194
613	ACOT8	0.209824299
614	KIAA1975	0.209684235
615	NDUFA7	0.20945738
616	MRPS23	0.209417775
617	E2F2	0.20919395
618	LIN9	0.209093011
619	ZNF367	0.209041906
620	MTX2	0.208831039
621	MCM4	0.208756253
622	TTC9B	0.208731832
623	ZRANB3	0.20865526
624	CDK5RAP1	0.208570647
625	C11orf42	0.20841026
626	TMEM177	0.208326514
627	CSTF3	0.208313842
628	NAGLU	0.208305228
629	PSMD3	0.208125798
630	PTPN1	0.208122419
631	BARD1	0.208008334
632	SLC25A17	0.207971472
633	HIF1AN	0.207843596
634	TP73	0.207447797
635	C5orf34	0.207417012
636	TRIAP1	0.207313167
637	H1FX	0.207195373
638	HSD17B10	0.207109551

639	POLA1	0.207090601
640	SUV39H1	0.207025459
641	COX7A2	0.20700106
642	FBXO21	0.206931172
643	FLJ35848	0.206865021
644	AZU1	0.206671489
645	NPEPL1	0.206629937
646	TPM3	0.206583648
647	FTSJ2	0.206578704
648	LSMD1	0.206547156
649	MFSD3	0.206505843
650	LARP4	0.206385413
651	ZNRF3	0.206154168
652	TGIF2	0.205639923
653	PDS5A	0.20560961
654	FDXR	0.205406086
655	C19orf55	0.205098255
656	GAS2L3	0.20502979
657	HES6	0.204730643
658	KLHL12	0.204535241
659	ZFYVE19	0.204457891
660	PTCD3	0.204190255
661	SLC19A1	0.204085978
662	DQX1	0.20395993
663	C20orf4	0.203900796
664	BNIP1	0.203897584
665	ARL6IP4	0.203404252
666	HRSP12	0.203067814
667	TATDN3	0.203024779
668	CST11	0.202940217
669	hCG_2028557	0.202912734
670	SMCR8	0.202903046
671	RFC2	0.202707686
672	RHBG	0.202494061
673	THOC2	0.202374413
674	NUP62CL	0.202345023
675	INSIG1	0.202328263
676	MOCS3	0.201990993
677	CCDC34	0.201778254
678	C14orf122	0.201588665

679	FBXO5	0.201440976
680	SNRPF	0.201395428
681	GOT1	0.201321642
682	SLMO2	0.201237196
683	COMMD4	0.201226674
684	NUP205	0.20115542
685	H2AFY	0.201112336
686	HINT1	0.20104033
687	NUBP2	0.201025461
688	PAGE1	0.201012259
689	SR140	0.200856077
690	NCOA5	0.200801955
691	SPAG1	0.200777535
692	NME1	0.200776395
693	DENR	0.200671006
694	COX7C	0.200541408
695	MAPBPIP	0.200447277
696	COQ7	0.200220728
697	SMC2	0.200187276
698	RDBP	0.200080641
699	CDC25B	0.200057617
700	DNAJB11	0.199930833
701	CRABP2	0.199881588
702	TRAF4	0.19986031
703	RNF213	0.199810392
704	NDUFAB1	0.199782489
705	TNRC4	0.199667284
706	LOC284352	0.199423998
707	IDE	0.199382743
708	LRRC46	0.19930627
709	C15orf21	0.199286682
710	RRN3	0.199266532
711	FDX1L	0.199204369
712	IRGC	0.199188157
713	RPN2	0.199071555
714	POFUT1	0.199032232
715	LOC441251	0.198960761
716	MRPS7	0.198735447
717	C12orf52	0.198575694
718	C3orf21	0.198565267

719	UCKL1	0.198546869
720	PSMD14	0.198512333
721	CCDC95	0.198396856
722	SLC9A3R1	0.198179487
723	SPATS2	0.198132813
724	DDX11	0.198078205
725	SENP1	0.197982089
726	PET112L	0.19793921
727	HARS2	0.19785991
728	ALG12	0.197859663
729	PDCD2L	0.197854374
730	NDUFS8	0.19780397
731	LOC144305	0.19770494
732	CTNNBL1	0.197704179
733	NOLC1	0.197581534
734	BAIAP2	0.197534141
735	PSMB6	0.19733703
736	DUT	0.197328943
737	SSX9	0.197310982
738	PIF1	0.197165293
739	KRT19	0.197011044
740	C20orf24	0.196978694
741	KIAA0692	0.196975797
742	MGC34821	0.196881592
743	CCDC22	0.196872029
744	BCAS4	0.196639497
745	COIL	0.196502973
746	C16orf14	0.19647965
747	LOC400506	0.196476379
748	ACBD6	0.19645283
749	POLR2G	0.196434554
750	TCEB1	0.196232826
751	PHKA1	0.196205656
752	PIP4K2B	0.196098238
753	EIF2B1	0.196003849
754	PSEN2	0.195972121
755	C16orf53	0.195956411
756	LOC116236	0.195733718
757	XRCC3	0.195721118
758	PRKAG1	0.195663276

759	MTBP	0.195597374
760	CBX1	0.195407923
761	VPS41	0.195199267
762	WNT3	0.195161239
763	TIPIN	0.195154246
764	PYCRL	0.195141155
765	FIGNL1	0.195119046
766	TPD52	0.195062786
767	HLTF	0.195057113
768	NLK	0.195006565
769	C20orf165	0.194916124
770	EIF4G2	0.194791141
771	MAGIX	0.194721104
772	EZH2	0.19457163
773	ALDH5A1	0.194569914
774	ACTR6	0.194510989
775	CISD2	0.194467413
776	C2orf52	0.194384566
777	EIF4A1	0.194197231
778	TMCO7	0.194054956
779	TIMM13	0.194045821
780	SOD1	0.194016741
781	LCE3B	0.193937006
782	RTTN	0.193834394
783	CACNA2D2	0.193773212
784	RUNDC3A	0.193579277
785	RCE1	0.193347499
786	SCO1	0.193256025
787	DDX39	0.193038414
788	PDZD11	0.193026297
789	ITPK1	0.192964244
790	RPAP3	0.192859271
791	SMPD4	0.192841225
792	TRMT5	0.192768074
793	LOC100125556	0.192570019
794	LOC606495	0.192329892
795	RBM39	0.192321514
796	RECQL5	0.192256493
797	PDRG1	0.192230403
798	MEA1	0.192175564

799	HMOX2	0.192052949
800	HMGCR	0.192033116
801	DHTKD1	0.191953348
802	ITGA2B	0.191757764
803	SUPT6H	0.191720793
804	MGC26597	0.191612378
805	SYMPK	0.191592736
806	C10orf83	0.191478965
807	SSX7	0.191408924
808	B3GALNT1	0.191372343
809	MSH2	0.19129352
810	FAM44C	0.191270849
811	FSD1L	0.191205617
812	LOC440356	0.191158724
813	HNRPM	0.191103875
814	SLC4A5	0.191078564
815	FAM98C	0.190984835
816	CKS2	0.190807799
817	PRKAR2A	0.19080315
818	CCT7	0.190709958
819	ATRIP	0.190662644
820	DNASE1	0.190550225
821	SFXN5	0.190474119
822	UBE2F	0.190426167
823	SOCS7	0.190326822
824	CDT1	0.19019611
825	COMTD1	0.190140084
826	OCRL	0.190110239
827	SIGIRR	0.19007979
828	GTF2IRD1	0.190077823
829	PGS1	0.189968125
830	PLEKHJ1	0.189951409
831	ZNF335	0.189950062
832	BCAT2	0.189948767
833	LONP1	0.18982925
834	RPL26L1	0.189583178
835	MTDH	0.189409593
836	FCMD	0.189404605
837	TMEM9	0.189378214
838	CTNNA1	0.189237922

839	LRRC23	0.189231535
840	CPD	0.189199761
841	PCK2	0.189167998
842	CCDC49	0.189088302
843	ALG6	0.189039309
844	ATPBD4	0.188789198
845	ICT1	0.188761956
846	SULT6B1	0.188670637
847	SLC28A2	0.188655288
848	CCT3	0.188579114
849	STMN1	0.18838869
850	RSAD1	0.1882087
851	SNRPD2	0.188187854
852	TUFM	0.188102744
853	NOL7	0.188077292
854	DPP3	0.188035508
855	EXOSC2	0.187925693
856	DDX55	0.187842418
857	ATPBD1C	0.187817482
858	FLJ30092	0.187795965
859	COG2	0.187789357
860	C20orf20	0.187781184
861	DLG3	0.187759449
862	IFT81	0.187743215
863	GNPDA1	0.187733771
864	LLGL2	0.187693799
865	GDAP1	0.18761533
866	DCI	0.187599542
867	PTCD1	0.187463641
868	MVK	0.187451795
869	HAX1	0.187428767
870	ABCB6	0.187343004
871	ACBD4	0.186716797
872	C20orf142	0.186567836
873	HIRIP3	0.186420757
874	TTC26	0.186413888
875	DONSON	0.186371099
876	XPO5	0.186325082
877	C14orf68	0.1862972
878	PARD6B	0.186241019

879	WDR18	0.186239094
880	LETM1	0.18621887
881	RHEBL1	0.186037289
882	GINS4	0.186018052
883	WDR72	0.186008558
884	ATG4D	0.186008225
885	MMAB	0.185850637
886	FLOT2	0.185767964
887	C6orf108	0.18571699
888	CRKRS	0.185601505
889	PAQR9	0.185526881
890	SAPS3	0.185466427
891	OR11A1	0.185434659
892	CXorf34	0.185398639
893	CNGB1	0.185267171
894	PYY	0.185210256
895	INHBC	0.185108133
896	MTFR1	0.184956311
897	PRDX3	0.1849279
898	FADS1	0.184901682
899	POP7	0.184881296
900	LOC150223	0.184874807
901	SDF2	0.184753613
902	GEMIN5	0.184631888
903	DHX15	0.184630627
904	COPS5	0.184616426
905	CCT8	0.184573312
906	COQ2	0.184515723
907	ABCB9	0.184498084
908	KHK	0.184482327
909	ALOXE3	0.184468789
910	GRIPAP1	0.184436806
911	YIF1B	0.184335625
912	SLC27A4	0.184281633
913	GRHL2	0.184193872
914	GRPEL2	0.184155052
915	TDG	0.184122623
916	SCAMP3	0.183908513
917	NRM	0.183796142
918	DNMT3B	0.183714995

919	TYSND1	0.183679479
920	NDUFAF1	0.183665393
921	GBL	0.183635091
922	TAZ	0.18363468
923	LRRC61	0.183605471
924	ARF3	0.183575516
925	HIST1H4C	0.183468288
926	PRPF31	0.183383564
927	DGKE	0.183255858
928	DNASE1L2	0.183237708
929	HIST1H1B	0.182786755
930	COX10	0.18275506
931	KBTBD4	0.182609233
932	HSPE1	0.182587478
933	EGLN2	0.182196729
934	IL28A	0.182158048
935	MGC33212	0.182140131
936	SLC35B1	0.182115995
937	C11orf77	0.182090989
938	XRCC6BP1	0.181857365
939	GABPB2	0.181728234
940	PI4KAP2	0.1816265
941	SNRPA	0.181445469
942	SAP130	0.181431804
943	FOXN1	0.181337211
944	FLVCR1	0.18133344
945	CSRP2BP	0.181208694
946	MKKS	0.181152581
947	DDHD1	0.181136193
948	SSX3	0.18107656
949	WDR54	0.18085753
950	RNF157	0.180781447
951	BTBD10	0.180740985
952	ARHGAP19	0.180603467
953	MLL2	0.180576362
954	MAT1A	0.180561084
955	ILVBL	0.180501785
956	ECH1	0.180448079
957	ATPAF2	0.180298752
958	ATP6V1C1	0.180282468

959	GPR77	0.180236004
960	APOBEC3B	0.180226304
961	RMND1	0.180217974
962	METTL1	0.18019461
963	FBXL20	0.180174583
964	UTP18	0.180153435
965	GCDH	0.180018065
966	SDSL	0.179970126
967	MGC99813	0.179951369
968	NCAPD3	0.179921981
969	E2F6	0.179917943
970	INTS7	0.179892576
971	ACTR5	0.179844011
972	C16orf13	0.179802647
973	KIF3B	0.179796869
974	CXXC5	0.179787266
975	OR1D4	0.179725486
976	C17orf80	0.179708339
977	KSR1	0.179644543
978	UBFD1	0.179632782
979	MTMR15	0.179581735
980	NUP98	0.179351727
981	NDE1	0.179302131
982	CLPP	0.179252539
983	USP7	0.179202503
984	C1orf25	0.179159522
985	EZH1	0.179089192
986	SSX5	0.179022438
987	PDCL3	0.17900952
988	CNOT1	0.178996043
989	WRB	0.178992208
990	PGAM1	0.178902365
991	SFXN1	0.178861036
992	IQCC	0.178818124
993	SNRPD1	0.178805156
994	CCDC117	0.178693485
995	COX15	0.178587291
996	C1orf111	0.178541026
997	THOC4	0.178509072
998	SSX1	0.178502388

999	ARFGEF1	0.17850077
1000	TIGD5	0.178464452
1001	PSMA7	0.178461713
1002	NAP1L1	0.178338838
1003	TELO2	0.178227508
1004	PPIA	0.178155786
1005	MED18	0.17815012
1006	HPRT1	0.178079559
1007	UBB	0.178033552
1008	NEDD1	0.178018505
1009	PIGL	0.177999828
1010	POLR2D	0.177910942
1011	CCDC97	0.177909332
1012	CLPTM1L	0.17789211
1013	PSMD9	0.177804603
1014	SSH2	0.177797689
1015	ENO2	0.177734282
1016	CUEDC2	0.177664582
1017	SULT1A2	0.177651895
1018	FBXO41	0.177519051
1019	FUT1	0.17749621
1020	EAF1	0.177259822
1021	FLJ40869	0.177166536
1022	SLC16A13	0.177136508
1023	N-PAC	0.177018083
1024	CABLES2	0.176986941
1025	HSP90AB1	0.176968879
1026	LOC124512	0.176963107
1027	LIN54	0.176959084
1028	SMUG1	0.176904756
1029	SNAPAP	0.176630012
1030	GSG1	0.176626822
1031	ATP5H	0.176567953
1032	NCOA6	0.176513554
1033	SMG7	0.176410285
1034	NIF3L1	0.176404568
1035	RAG1AP1	0.176320232
1036	TUBA3D	0.17626349
1037	PCYOX1L	0.176189318
1038	GPS1	0.176142509

1039	RTEL1	0.176135552
1040	SULT1C2	0.176081509
1041	ATP5G1	0.175937434
1042	ZDHHC24	0.175894061
1043	MFAP1	0.17583667
1044	FLYWCH2	0.175757416
1045	FN3KRP	0.175745691
1046	NUP43	0.175705395
1047	LSG1	0.175694618
1048	ASPSCR1	0.175629591
1049	ZKSCAN2	0.175547803
1050	APOBEC3D	0.175506831
1051	GHDC	0.175474467
1052	USP32	0.175421177
1053	FAM86A	0.175419392
1054	NR2F6	0.175354873
1055	DNAJC5	0.175339432
1056	MGC24125	0.175244956
1057	LARP1	0.175231622
1058	KIAA1727	0.175231235
1059	KRT37	0.175227257
1060	PSMA6	0.175214576
1061	OAS3	0.17503923
1062	DOLK	0.174969553
1063	PIGO	0.174964343
1064	LRFN2	0.174949447
1065	ZNF534	0.174882568
1066	PPP1CA	0.174839638
1067	PARD6A	0.174808781
1068	C16orf68	0.174784118
1069	ELAVL3	0.174774771
1070	FKBP4	0.174749872
1071	EXOSC5	0.17471731
1072	SRXN1	0.174568896
1073	SLC6A18	0.174523131
1074	C3orf37	0.174471711
1075	C18orf54	0.174470132
1076	SLC25A11	0.17446588
1077	YWHAB	0.174460749
1078	PUS1	0.174358779

1079	XTP3TPA	0.174319762
1080	C19orf46	0.174112189
1081	FOXRED2	0.174091185
1082	TMEM161A	0.173842878
1083	SNRPB	0.173795136
1084	C6orf211	0.173575846
1085	CPSF3	0.173572048
1086	TIMM17A	0.173562502
1087	USP37	0.173434338
1088	C17orf71	0.173433214
1089	ERCC3	0.173424559
1090	RBM33	0.17331756
1091	RBM35A	0.173306949
1092	CBLN3	0.173299829
1093	SRP68	0.173222268
1094	CENPN	0.173057228
1095	C6orf167	0.173034985
1096	ZNF664	0.173007855
1097	NSUN2	0.172952923
1098	IL17RB	0.172866455
1099	PSMA5	0.17284538
1100	HNRPF	0.172799345
1101	C16orf42	0.17268992
1102	ATXN2	0.172672348
1103	SLC10A5	0.172670547
1104	ABCC1	0.172659654
1105	CYC1	0.172637087
1106	KPTN	0.172590922
1107	NDUFA13	0.172438621
1108	TEX264	0.172422652
1109	BLOC1S1	0.172326575
1110	LRBA	0.172266588
1111	CCNE1	0.172229471
1112	LINGO4	0.17218536
1113	PDDC1	0.171907195
1114	L2HGDH	0.171895794
1115	NPM1	0.1718023
1116	AP4M1	0.171774431
1117	WFDC10B	0.171680263
1118	SFRS2	0.171580716

1119	PIGS	0.171562712
1120	VRK1	0.171544248
1121	NUDC	0.171489233
1122	PIGM	0.171407559
1123	UROS	0.171346997
1124	IAH1	0.171327891
1125	DNAJC14	0.171293522
1126	KRTAP11-1	0.171271757
1127	KIAA0082	0.17123981
1128	P117	0.171210668
1129	BSN	0.171135269
1130	MSTO1	0.17111741
1131	LOC283345	0.171066417
1132	C16orf79	0.171064396
1133	RPS6KC1	0.171022534
1134	SNF8	0.170956155
1135	MIF4GD	0.170881037
1136	PCYT1A	0.170879006
1137	HNRNPR	0.170678917
1138	RBM14	0.170668584
1139	PANK3	0.170611183
1140	THAP8	0.170577202
1141	WIBG	0.170577002
1142	FAM104B	0.170570805
1143	DDX23	0.170563365
1144	AP3B1	0.170551941
1145	FAM40B	0.170542249
1146	HSPB1	0.170474506
1147	USP9Y	0.170464059
1148	EXOSC9	0.170374322
1149	SYP	0.170354793
1150	COQ9	0.170302248
1151	PRPF19	0.17022841
1152	LYK5	0.17013387
1153	RNPS1	0.170053178
1154	DKFZp434K1815	0.170020294
1155	MRPL38	0.170018349
1156	TOPBP1	0.169972186
1157	GSK3A	0.169929786
1158	HSP90AA1	0.169854979

1159	DUSP14	0.169821417
1160	GHITM	0.169754158
1161	PPID	0.169747766
1162	CDKN2AIPNL	0.169736282
1163	KIAA1467	0.169726029
1164	NUP62	0.169709337
1165	SC4MOL	0.169674446
1166	TAF15	0.16960545
1167	MED1	0.169599795
1168	C12orf30	0.169587762
1169	TSPAN17	0.169568747
1170	IFT122	0.169292901
1171	PAK6	0.169225637
1172	LRTM2	0.169204522
1173	NCL	0.169195839
1174	CEP27	0.169181927
1175	VPS37C	0.169148051
1176	CA14	0.169121745
1177	KIAA0319	0.169080837
1178	ZCRB1	0.168991006
1179	RHEB	0.16883492
1180	ZDHHC13	0.168831016
1181	APOOL	0.168828013
1182	FBXW9	0.168818869
1183	SNRPC	0.168756101
1184	PSRC1	0.16874251
1185	NECAP1	0.168733653
1186	FAM50A	0.168706084
1187	SAE2	0.168673423
1188	PRRT3	0.16861392
1189	WDR53	0.168339918
1190	HEATR2	0.168313063
1191	JTV1	0.168005169
1192	STAT5B	0.168004159
1193	PPP2R5D	0.167921292
1194	NUDCD2	0.167920499
1195	RABL4	0.167919037
1196	MORG1	0.167913615
1197	POLR2H	0.167883309
1198	MTL5	0.167842926

1199	PRTN3	0.167830916
1200	VAPB	0.167748792
1201	OPRD1	0.167718328
1202	EP400NL	0.167587139
1203	PTPLAD1	0.167522633
1204	CHRNA9	0.167482844
1205	HMGB3	0.167453575
1206	PRMT3	0.167419452
1207	LRRCC1	0.167415438
1208	FLAD1	0.167414979
1209	WDR51B	0.167383946
1210	MRPL14	0.167375027
1211	SH2D6	0.167358437
1212	HSD11B2	0.166957793
1213	COPZ1	0.166937261
1214	UNQ1887	0.16690524
1215	TBC1D7	0.16686943
1216	PTDSS1	0.166855335
1217	LRRC57	0.166801385
1218	DKFZp779O175	0.166706971
1219	C2orf29	0.166700567
1220	RNF215	0.166672969
1221	NSUN5	0.166665335
1222	NIT2	0.166609692
1223	AZI1	0.166605809
1224	SLC39A3	0.166560439
1225	FBXL10	0.166484566
1226	TAF5	0.16646905
1227	MORN2	0.166360832
1228	SLC25A3	0.166344572
1229	ARL3	0.166332131
1230	YARS2	0.16628958
1231	LRRC37B2	0.166220316
1232	ZMYND19	0.166177146
1233	SPAG4	0.166169063
1234	LRRIQ2	0.16613415
1235	ELSPBP1	0.16600566
1236	SDF2L1	0.165995124
1237	SLC25A35	0.165994815
1238	TRAPPC6A	0.165973288

1239	THOP1	0.165956102
1240	PNPT1	0.16594305
1241	XPNPEP3	0.165942435
1242	FANCA	0.165934871
1243	C12orf36	0.16592091
1244	CCHCR1	0.165854951
1245	CNTD1	0.165830401
1246	STK16	0.16580947
1247	NAGPA	0.165802974
1248	HUWE1	0.16577448
1249	PCBD2	0.165770908
1250	RBM10	0.165627509
1251	UBL5	0.165577281
1252	CRP	0.165560727
1253	ATXN10	0.165482596
1254	LOC90835	0.165376029
1255	EIF1	0.165329634
1256	SLC35A2	0.165327254
1257	RPS6KL1	0.165323276
1258	GALK1	0.165247855
1259	BCDIN3D	0.165193602
1260	BRD8	0.165112513
1261	NME3	0.165094506
1262	RBM45	0.165053571
1263	LRRC37B	0.164791742
1264	PYCR2	0.164790612
1265	CCT5	0.164788683
1266	TMEM16J	0.164768053
1267	PTBP1	0.164703724
1268	SORD	0.164682221
1269	METTL2B	0.164553151
1270	DAK	0.164524595
1271	SCAND1	0.16445167
1272	NIPA1	0.164371556
1273	C11orf80	0.164315036
1274	USP39	0.164280599
1275	CCDC55	0.164180131
1276	TMEM143	0.164063924
1277	ATP5G2	0.16400443
1278	MB	0.163898657

1279	KIAA0391	0.163810981
1280	C7orf50	0.163804559
1281	C6orf26	0.163699351
1282	POP5	0.163659662
1283	ZNF76	0.163627101
1284	FARSA	0.163443435
1285	POLR3A	0.163440712
1286	C12orf34	0.163409187
1287	EMP2	0.163403942
1288	RLTPR	0.163378047
1289	SART3	0.163349046
1290	TOMM40	0.163239084
1291	LRP11	0.163237824
1292	ACPT	0.163204933
1293	RABGGTA	0.163157171
1294	TMEM54	0.163142565
1295	SYT2	0.163127285
1296	MKS1	0.163045292
1297	PFAS	0.163040355
1298	CBX8	0.163023096
1299	ENOPH1	0.163022115
1300	C19orf10	0.162984453
1301	TPCN2	0.162975709
1302	SLIT1	0.162951467
1303	GDPD3	0.162925188
1304	FAM33A	0.162882853
1305	XRCC4	0.162808327
1306	MRPL43	0.162789696
1307	NDUFV2	0.162767724
1308	RDH12	0.162759419
1309	RFC3	0.16270567
1310	MTERFD1	0.162691136
1311	DUS4L	0.162686621
1312	CELSR3	0.162664668
1313	TRMT6	0.162586989
1314	DMRTB1	0.162579853
1315	LOC51233	0.162540198
1316	ATP8B1	0.162433234
1317	HDAC8	0.162229301
1318	HIST1H2AJ	0.162142794

1319	MTCH2	0.162135914
1320	ROD1	0.162013258
1321	C17orf89	0.161970518
1322	SMARCD1	0.161968459
1323	SLC16A14	0.161967999
1324	TSNAX	0.161918539
1325	PFDN2	0.161752639
1326	PSMD4	0.161731166
1327	BCL2L1	0.161657188
1328	HLCS	0.161641024
1329	DNTTIP1	0.161639735
1330	C1orf124	0.161593446
1331	SNRPD3	0.161568396
1332	PKP3	0.161542248
1333	TUBA3C	0.161537169
1334	SLC29A3	0.161501123
1335	C14orf179	0.161489775
1336	MAD2L1BP	0.161472904
1337	RG9MTD2	0.161451282
1338	TUBB	0.161393577
1339	TAAR5	0.161336671
1340	C20orf151	0.161333809
1341	C9orf43	0.16117829
1342	FLJ21865	0.161168631
1343	P2RX3	0.161056342
1344	ELMOD2	0.160939075
1345	PRKDC	0.160913632
1346	POLRMT	0.160868871
1347	GAPVD1	0.160765287
1348	FAM122B	0.160729726
1349	TUBGCP5	0.16070966
1350	HNF1B	0.160689403
1351	PSMB5	0.160651998
1352	MRPL21	0.160320888
1353	ATG9B	0.160270996
1354	C8orf55	0.16026848
1355	C12orf56	0.160222617
1356	YEATS4	0.160199517
1357	SHARPIN	0.160139196
1358	PIGP	0.16013509

1359	MTNR1A	0.160126432
1360	BRP44	0.160083204
1361	BLVRA	0.160050377
1362	TMEM80	0.160016106
1363	CPT1A	0.160006671
1364	TM9SF4	0.159929082
1365	SAP30L	0.159795158
1366	PLAC1	0.159778832
1367	NAPB	0.159581002
1368	CLCN2	0.159558107
1369	CCBL1	0.159533917
1370	C21orf58	0.159473896
1371	TCTN2	0.159446025
1372	MPDU1	0.159401128
1373	PLAC4	0.159351623
1374	RNF181	0.159188531
1375	EXOC2	0.159144548
1376	ASTL	0.159111651
1377	C12orf10	0.15902507
1378	MYT1L	0.158846294
1379	NT5M	0.158845491
1380	EXDL1	0.15884126
1381	TKT	0.158826395
1382	ANKRD39	0.158782568
1383	C6orf81	0.15876158
1384	HIST1H4D	0.158746916
1385	CHMP5	0.158743332
1386	C3orf22	0.158741262
1387	KIAA1530	0.158735916
1388	DDEF2	0.158596965
1389	WDSOF1	0.158577139
1390	ARMC6	0.158551695
1391	NCKIPSD	0.158536773
1392	PRDX1	0.158508463
1393	SLC4A1	0.158506319
1394	TMEM161B	0.158491524
1395	TMEM70	0.158466169
1396	TOM1L1	0.158452784
1397	TARDBP	0.158449924
1398	TMEM186	0.158438664

1399	RARA	0.158284199
1400	HAL	0.15823505
1401	RAE1	0.158219531
1402	FLJ11506	0.158200309
1403	GPKOW	0.15812493
1404	SPRR2G	0.158122267
1405	WDR24	0.158100878
1406	OTP	0.158068062
1407	KIFC2	0.158063902
1408	IFRD2	0.157969297
1409	NCAPH2	0.157924776
1410	MANEAL	0.157889369
1411	MRPL12	0.157797483
1412	CRYGA	0.157782607
1413	GPR19	0.157757568
1414	PEX1	0.157687849
1415	NSUN5C	0.157580345
1416	SHMT1	0.157502282
1417	C1orf43	0.157474882
1418	CNTD2	0.15746025
1419	C11orf58	0.157430892
1420	YTHDF1	0.157365153
1421	NCAPD2	0.157319504
1422	XRCC6	0.157315369
1423	DOC2A	0.157122065
1424	TMEM62	0.157075195
1425	TRPC4AP	0.156936597
1426	NDUFA12	0.156804358
1427	CST8	0.15673726
1428	NSFL1C	0.156663681
1429	MGC34796	0.156656215
1430	HEXDC	0.156635781
1431	SPR	0.156620425
1432	AHCY	0.156505413
1433	HEBP1	0.15649987
1434	GSDMDC1	0.156436096
1435	KCNK9	0.156435989
1436	TRIM37	0.15641644
1437	USP6	0.156358127
1438	MECR	0.156347613

1439	MRPL13	0.156276344
1440	OR2T34	0.15624146
1441	NSL1	0.156234889
1442	CCDC137	0.156202782
1443	RABIF	0.156194402
1444	RAB11FIP3	0.156181549
1445	OR2B11	0.156149341
1446	USP18	0.156111842
1447	CWF19L1	0.15610252
1448	ZNF341	0.155965333
1449	IMPA1	0.155936563
1450	TOMM70A	0.155792914
1451	FLJ32658	0.155728639
1452	MED14	0.155717769
1453	ATP5G3	0.155698007
1454	LOC541469	0.15566373
1455	C14orf80	0.155604345
1456	FLJ32549	0.155602616
1457	DCLRE1A	0.155541411
1458	STRC	0.155454652
1459	DNM1L	0.155442343
1460	MGST2	0.155415853
1461	OR51G1	0.155410256
1462	MAPK8IP3	0.155395741
1463	KIAA1542	0.155350534
1464	ANKRD40	0.155336225
1465	CARHSP1	0.1552723
1466	MLLT6	0.155264013
1467	GRWD1	0.155260851
1468	CPNE6	0.155214017
1469	SULT2B1	0.155149674
1470	MMP24	0.155147016
1471	TANC2	0.155109241
1472	CYP2R1	0.155107705
1473	ALG10	0.155083755
1474	TMEM103	0.155052869
1475	CORO7	0.15498838
1476	LSDP5	0.154861296
1477	RPGRIP1L	0.154826563
1478	HIST1H4L	0.154823118

1479	EIF4A3	0.154811153
1480	GMEB2	0.154647536
1481	PSMD2	0.154435292
1482	XCR1	0.154321994
1483	HARS	0.154206793
1484	TMEM14A	0.154126841
1485	HSP90B1	0.154110535
1486	RAD50	0.154067375
1487	PHF20	0.153955873
1488	CECR2	0.1539379
1489	LOC339229	0.153918242
1490	HIST1H4B	0.15390124
1491	TCTN1	0.15386911
1492	PTCD2	0.153718045
1493	AP4E1	0.153667953
1494	CATSPER2	0.153640355
1495	GFM2	0.153601837
1496	DUSP3	0.153478328
1497	OCEL1	0.153421674
1498	GNPNAT1	0.153383628
1499	POLR1B	0.153377584
1500	NOM1	0.153343792
1501	ZMAT2	0.153302229
1502	RTN4RL2	0.153262545
1503	SLC19A2	0.153190897
1504	EPB41L5	0.153149014
1505	LOC92345	0.153132683
1506	CBX4	0.153069348
1507	SP2	0.153060839
1508	FIBP	0.153028786
1509	C8orf33	0.15302807
1510	ULK1	0.152989871
1511	2'-PDE	0.152979959
1512	NUP155	0.15297449
1513	OSBP2	0.152969818
1514	TMEM189	0.15294047
1515	GRIK3	0.152920514
1516	SLC39A11	0.152773573
1517	SPATA2	0.152772683
1518	KCTD5	0.152726129

1519	C20orf152	0.152720353
1520	HNRNPL	0.152681357
1521	SLCO6A1	0.152659202
1522	RAD9B	0.152631865
1523	ARPC3	0.152511829
1524	FLJ10815	0.152479344
1525	C16orf24	0.152463882
1526	KIAA1787	0.152390969
1527	HNRPAB	0.152378958
1528	IL8RA	0.152332668
1529	H2AFJ	0.152276433
1530	UBAP2L	0.152199699
1531	CST9L	0.152167878
1532	RRM2B	0.15215161
1533	SPACA4	0.152149085
1534	JPH1	0.15209899
1535	TSHB	0.151978312
1536	CETN2	0.151962938
1537	ELOVL6	0.151951003
1538	FLJ23356	0.151944081
1539	KIAA0146	0.151940091
1540	LOC402164	0.151939409
1541	PCLO	0.151894498
1542	ASCL1	0.151770125
1543	MRPL36	0.151760867
1544	SFRS3	0.151733973
1545	LETM2	0.151731255
1546	PRRT2	0.151666364
1547	TUBB2C	0.151641521
1548	MRPS18A	0.151571762
1549	SLC35D3	0.151559215
1550	AIFM3	0.151531238
1551	NONO	0.151521853
1552	COG7	0.151514192
1553	CRADD	0.15146966
1554	SEMA4G	0.151366404
1555	TPI1	0.151310607
1556	SCNN1B	0.15129834
1557	DCK	0.151282347
1558	AKAP1	0.151233008

1559	LMNB2	0.151136972
1560	NTSR2	0.15105463
1561	DOLPP1	0.151010714
1562	NIT1	0.151007212
1563	TH1L	0.151003166
1564	KIAA0196	0.150968772
1565	RBM35B	0.150867711
1566	HNRPH1	0.150864656
1567	CDC27	0.150852061
1568	REPS2	0.150841527
1569	TMEM63B	0.150790364
1570	TMED9	0.150757019
1571	ABHD12	0.150744816
1572	MRPL27	0.150731539
1573	APTX	0.150618806
1574	SEMA3F	0.150607198
1575	ADAM7	0.150605848
1576	E4F1	0.150578673
1577	SUV420H2	0.150474843
1578	SLBP	0.150416144
1579	CCDC123	0.150411351
1580	CHRNG	0.150291711
1581	APOO	0.1502851
1582	FAM83H	0.150193673
1583	SLC7A11	0.150133593
1584	LYCAT	0.150128947
1585	ADAR	0.15005759
1586	LMBRD2	0.150015025
1587	IL19	0.14995749
1588	MGC35295	0.149951649
1589	FABP6	0.14994666
1590	AP1S1	0.149930633
1591	LYZL2	0.149923174
1592	DDX41	0.149914082
1593	ARL16	0.14988939
1594	C17orf70	0.149887512
1595	C14orf73	0.149840632
1596	GPRC5D	0.149715356
1597	PRG2	0.149714197
1598	ACTBL1	0.149639618

1599	FGD1	0.14962684
1600	LYG2	0.149583428
1601	METTL3	0.14955916
1602	C3orf1	0.149489331
1603	DNAJC5G	0.149438138
1604	TRIM7	0.149427152
1605	MTP18	0.149304154
1606	SSNA1	0.149224891
1607	SLC8A2	0.149113774
1608	BPNT1	0.149107978
1609	PDHX	0.149094766
1610	ISOC2	0.149056536
1611	CD300LB	0.149053996
1612	TUSC4	0.148977498
1613	UBQLN4	0.148902228
1614	SYT7	0.148896278
1615	SYS1	0.148893118
1616	PFKFB4	0.148859698
1617	TARBP2	0.148858946
1618	CALM3	0.148802919
1619	KIAA1666	0.14876916
1620	RAB26	0.148747132
1621	NT5C3	0.148717232
1622	ABCC5	0.148704458
1623	ZNF428	0.148650538
1624	KRT18	0.148617842
1625	RTF1	0.148594516
1626	OSGIN2	0.148594199
1627	RANBP1	0.148576093
1628	PEO1	0.148559913
1629	MCM5	0.148506663
1630	CECR5	0.148442403
1631	C19orf52	0.148437144
1632	SPANXD	0.14840814
1633	ZNF589	0.148335198
1634	NDUFB9	0.14832074
1635	ARMC1	0.148188762
1636	EDG3	0.148165891
1637	SLC24A5	0.148151759
1638	CEACAM8	0.148138904

1639	BDH1	0.148107741
1640	MLSTD2	0.148090253
1641	ILF3	0.148026462
1642	GMPS	0.148001282
1643	C6orf136	0.147996845
1644	ZSWIM1	0.147978814
1645	SPNS1	0.147902138
1646	ZNF507	0.147894023
1647	UQCRQ	0.147881419
1648	OTUB2	0.147780238
1649	OAS1	0.147681165
1650	HTR1E	0.147658188
1651	CEACAM5	0.147579829
1652	MDC1	0.147549414
1653	UTP15	0.147546646
1654	UCHL5IP	0.147507943
1655	SLC38A6	0.147409888
1656	MRPL40	0.147328193
1657	WWP1	0.147298814
1658	FAM44B	0.147235251
1659	RTBDN	0.147178164
1660	LASS4	0.14711412
1661	VPS29	0.147060344
1662	DAZAP1	0.147037544
1663	ORC5L	0.147026364
1664	SH3BP5L	0.147012121
1665	WDR90	0.146932017
1666	DEFB119	0.14691673
1667	SYT1	0.146887111
1668	CCDC77	0.146830548
1669	LOC653314	0.146816258
1670	LOC340069	0.146678506
1671	TCTA	0.146648568
1672	EML2	0.14663063
1673	PDE6D	0.146599686
1674	SNRPB2	0.146596297
1675	DIS3L2	0.146535792
1676	NFE2	0.146532727
1677	OSGEPL1	0.146461316
1678	GDF15	0.146391571

1679	GBA	0.146364505
1680	TAC3	0.146277864
1681	MRGPRX2	0.146231375
1682	DDX51	0.146210295
1683	PSMA1	0.146166814
1684	MGC88374	0.146043363
1685	PROCA1	0.146028039
1686	H3F3A	0.146017892
1687	C9orf165	0.145968828
1688	ORMDL3	0.145964388
1689	PSMD1	0.145961481
1690	ZNF318	0.145954043
1691	SLC27A2	0.145934839
1692	EIF2S1	0.145923135
1693	LMTK2	0.14590406
1694	PPCDC	0.145894883
1695	ABHD11	0.145851643
1696	ULK2	0.145816606
1697	OMA1	0.145713314
1698	MPP3	0.145709619
1699	PH-4	0.145672766
1700	FLYWCH1	0.145637434
1701	FBXW11	0.145631712
1702	HMBS	0.145631475
1703	VPS8	0.145601624
1704	DYX1C1	0.145593668
1705	CLDN7	0.145591365
1706	HN1	0.145588201
1707	MRTO4	0.145525222
1708	LYSMD1	0.145515797
1709	DVL2	0.145484405
1710	CD2AP	0.145456165
1711	ZNF706	0.145452734
1712	UBE2A	0.145448358
1713	OR4C3	0.145419204
1714	VDAC3	0.145418669
1715	CCT2	0.145414372
1716	CEL	0.145376894
1717	FZD2	0.145287348
1718	PWP1	0.145202659

1719	GEMIN6	0.145193924
1720	ALG3	0.145193371
1721	RAD17	0.145146349
1722	HBG2	0.145145045
1723	LOC388969	0.145139336
1724	MORN3	0.145128856
1725	HSD17B7	0.145040633
1726	LRRC37A2	0.145036334
1727	LACTB2	0.145014389
1728	WDR92	0.144964306
1729	RANBP9	0.144943932
1730	FBXO24	0.144939787
1731	C19orf57	0.144926105
1732	SUSD4	0.144909075
1733	C10orf6	0.144877025
1734	ADAM21	0.144857937
1735	HYLS1	0.144851831
1736	ATP2A2	0.144803931
1737	NOL10	0.144792326
1738	NUBP1	0.144791299
1739	TADA1L	0.144787632
1740	PDCD11	0.144748096
1741	tcag7.1015	0.144734155
1742	GDPD1	0.144729179
1743	WDR8	0.144728521
1744	OR52H1	0.144629763
1745	PEX5L	0.144618271
1746	KLC2	0.144586379
1747	TNFRSF18	0.144534305
1748	EPS15L1	0.144518792
1749	C10orf28	0.14448894
1750	HNRPD	0.144457481
1751	GLE1L	0.144441049
1752	CYB561	0.144408614
1753	SRPK1	0.144291324
1754	KCNQ2	0.144237083
1755	C3orf60	0.144235661
1756	MED29	0.14420887
1757	TRH	0.144160426
1758	HSPB8	0.144135375

1759	TAF10	0.144133027
1760	LEMD2	0.144120017
1761	SKP1A	0.144076442
1762	TRAPPC5	0.144008793
1763	C1orf71	0.143876034
1764	HIST1H2AI	0.143854092
1765	ADCY6	0.14382442
1766	P15RS	0.143816265
1767	B9D1	0.143816222
1768	EMID1	0.14378245
1769	CRNKL1	0.143719338
1770	NAT10	0.143689562
1771	FASTKD1	0.14366167
1772	LENG8	0.143657702
1773	SS18L1	0.143632443
1774	ASB8	0.143610694
1775	CPEB4	0.143586703
1776	RMND5B	0.143562681
1777	NSMCE1	0.143547772
1778	HDAC2	0.143540824
1779	ITLN2	0.143510066
1780	KIAA0701	0.143508431
1781	PMVK	0.143501949
1782	DARS	0.143460326
1783	C2orf44	0.143430449
1784	JMJD1B	0.143407354
1785	PDYN	0.143393233
1786	DDX49	0.143366321
1787	C1QBP	0.143348211
1788	FAM113A	0.143346907
1789	CD320	0.143326564
1790	PPP1CC	0.143244325
1791	PIN1L	0.143234907
1792	FLJ11184	0.143197146
1793	VPS13B	0.143160391
1794	DBF4	0.143157555
1795	FMR1	0.143155041
1796	ELMO2	0.14310926
1797	HSPA8	0.142992119
1798	DOHH	0.142882097

1799	CCS	0.142838699
1800	C12orf41	0.142827888
1801	TLE6	0.142795577
1802	FRMPD3	0.142781947
1803	SEC61A2	0.142781248
1804	HIST2H2AC	0.142769507
1805	DIP2B	0.14276481
1806	HDAC1	0.14275034
1807	PFDN4	0.142738285
1808	ASB10	0.142736877
1809	FXR2	0.14266869
1810	NDUFB11	0.142655575
1811	PPM1G	0.142622939
1812	GNGT1	0.142619002
1813	PIN4	0.142569629
1814	ALOX15	0.142564477
1815	C19orf30	0.142534566
1816	MRPL47	0.142491742
1817	UBE2O	0.142463167
1818	NAT5	0.142426313
1819	PARG	0.142416729
1820	PI4KA	0.142354683
1821	RBM41	0.142349858
1822	LOC400590	0.14233573
1823	GRAMD1A	0.142235235
1824	CELSR1	0.142215252
1825	SRPK2	0.142188733
1826	PAIP2	0.142178036
1827	AXIN1	0.142145846
1828	ACBD5	0.142126695
1829	AGBL2	0.142093959
1830	hCG_16001	0.142051712
1831	KIAA1799	0.142033255
1832	PEBP1	0.142003908
1833	KIAA1303	0.141994111
1834	RBBP9	0.141952377
1835	MAGEA11	0.141946857
1836	DHX34	0.141878218
1837	OR4A15	0.141825182
1838	CXorf40B	0.141795727

1839	FECH	0.141759382
1840	UBE2N	0.141734038
1841	ACVR1B	0.141732766
1842	DPM2	0.14168911
1843	SLCO1B1	0.141667393
1844	MRPL49	0.141605156
1845	PIK3R4	0.141604721
1846	METT5D1	0.14157049
1847	CHRM1	0.141560719
1848	TEGT	0.141548515
1849	IHPK3	0.141516264
1850	TARS2	0.141494496
1851	SARS2	0.141485382
1852	ZFY	0.141455075
1853	GRINA	0.141453652
1854	AAMP	0.141421708
1855	MCCC2	0.141405778
1856	CCDC86	0.141352206
1857	LOC653319	0.141349421
1858	SLC1A2	0.141325797
1859	PIP5K1A	0.141319134
1860	JMJD6	0.141318231
1861	F12	0.141306743
1862	TNNT1	0.14126599
1863	TRPM5	0.141226432
1864	ORC3L	0.141164848
1865	FLNB	0.14114471
1866	PGK1	0.140994713
1867	PRPF6	0.140973628
1868	FAM20B	0.140927481
1869	CXorf27	0.140852629
1870	SIX4	0.140822275
1871	NDUFS3	0.140795021
1872	OR4M1	0.140727312
1873	ZFYVE27	0.140677755
1874	TTF2	0.140627556
1875	HINT2	0.14058084
1876	C20orf12	0.140471786
1877	C2orf15	0.14042754
1878	C10orf88	0.1403785

1879	MYEF2	0.14037435
1880	FAM130A1	0.140368564
1881	GTF3C5	0.140336621
1882	CPNE3	0.140335072
1883	FAM83A	0.140199793
1884	FKBPL	0.140175407
1885	KIAA0195	0.140149359
1886	UQCRC1	0.140127004
1887	AS3MT	0.140088785
1888	XRCC5	0.14005967
1889	FARSB	0.140045627
1890	SRP54	0.140028802
1891	SLC9A4	0.139975344
1892	ZSWIM7	0.139939692
1893	PARP1	0.13987937
1894	ADAD1	0.139858832
1895	RBBP4	0.139844698
1896	MAGEA1	0.139832136
1897	PRDX2	0.13976349
1898	FLJ32065	0.139730794
1899	POLR3F	0.139642741
1900	TMEM16G	0.139635221
1901	HSPC152	0.139611133
1902	TXNRD1	0.139516726
1903	FLJ40504	0.139516677
1904	GSTCD	0.13951394
1905	LRFN4	0.139472945
1906	PFN4	0.139443896
1907	RP11-11C5.2	0.139440641
1908	MLN	0.13943645
1909	CKAP2	0.139376704
1910	PLA2G12B	0.139359957
1911	BCS1L	0.139298115
1912	TRIM25	0.139228985
1913	ZBTB9	0.139223437
1914	SLC26A11	0.139202042
1915	XPO6	0.139104628
1916	NPFFR1	0.139095847
1917	PARN	0.139078961
1918	C2orf48	0.139040779

1919	CDC42EP4	0.139009719
1920	CCDC5	0.139006835
1921	H1F0	0.138952073
1922	FBXW8	0.138922192
1923	FASTKD3	0.138909019
1924	EIF2AK1	0.138899072
1925	ALDH16A1	0.138895516
1926	PSMA2	0.138797119
1927	RDH14	0.138784689
1928	PCGF6	0.138740762
1929	KIAA1267	0.138712741
1930	CHCHD3	0.138643279
1931	DHCR24	0.138636328
1932	ELA2B	0.138627696
1933	FNIP1	0.138593756
1934	SPINT2	0.138588019
1935	LYRM1	0.138469677
1936	CC2D1A	0.138395658
1937	C14orf140	0.1383203
1938	GLT8D3	0.138243726
1939	IDH3B	0.138224174
1940	ACCN3	0.138198756
1941	C6orf49	0.138180802
1942	DAB1	0.138157583
1943	CAPRIN2	0.138156266
1944	SULT1A1	0.138149316
1945	C12orf25	0.138134721
1946	RPL19	0.138115274
1947	RIC8B	0.138069581
1948	TUBB8	0.138013617
1949	COX5B	0.137992103
1950	CHST5	0.137953002
1951	ATIC	0.137869997
1952	QTRT1	0.137786461
1953	AMZ2	0.137746848
1954	POTE15	0.137708902
1955	N6AMT1	0.137695028
1956	MGC13008	0.137686775
1957	ZMYM1	0.137671022
1958	IFIT1L	0.137649439

1959	TP53BP1	0.137621744
1960	LAPTM4B	0.137502681
1961	METTL2A	0.137500489
1962	ANXA9	0.137475494
1963	LCE3D	0.137471089
1964	ERCC8	0.13739621
1965	SCLY	0.137367151
1966	XPOT	0.13732158
1967	ZNF771	0.13731572
1968	SBK1	0.13726305
1969	CLC	0.137248989
1970	DHDH	0.137245933
1971	SFI1	0.137155603
1972	C3orf26	0.137125174
1973	DHX16	0.137087648
1974	MKRN3	0.137064027
1975	C5orf37	0.137047967
1976	PARP2	0.137015479
1977	RHBDD3	0.136965798
1978	IL1F9	0.13696196
1979	FAM82B	0.136873176
1980	REEP2	0.13679608
1981	SLITRK1	0.13669804
1982	RNF123	0.136694065
1983	TMEM99	0.136691804
1984	CMAS	0.136669506
1985	CNOT6	0.136653749
1986	SFRP5	0.136612945
1987	HSP90AB6P	0.136594212
1988	CGN	0.136544189
1989	ADIPOR2	0.136513551
1990	WBSCR27	0.136481292
1991	FUSIP1	0.136475798
1992	PIN1	0.136368333
1993	APAF1	0.136302485
1994	FGFR3	0.136289151
1995	ZNF165	0.136288367
1996	C11orf49	0.136283927
1997	ZC3H12D	0.136236591
1998	MRPS36	0.136226073

1999	C20orf175	0.136226033
2000	MRPL42	0.136193223
2001	SNAPC3	0.136164679
2002	BRMS1	0.136160195
2003	DERL1	0.136128367
2004	RARS	0.136109556
2005	STK36	0.13607288
2006	DHX33	0.13599583
2007	SLC20A1	0.135991809
2008	ONECUT2	0.135988582
2009	GPHN	0.135985309
2010	ZYG11A	0.135950091
2011	YIF1A	0.135928904
2012	CYB5R4	0.135922069
2013	CHD6	0.13590676
2014	IL1RAPL2	0.135865822
2015	CCDC58	0.135796928
2016	HSPBP1	0.135771424
2017	FANCC	0.135699245
2018	MCCD1	0.135685441
2019	TMEM147	0.135678167
2020	PTGES2	0.135507431
2021	HRAS	0.135455232
2022	PPP1R10	0.135441016
2023	RUSC1	0.135256347
2024	RBBP5	0.135253101
2025	KIAA1618	0.135230225
2026	WDR34	0.135227576
2027	MRPS15	0.135197822
2028	ST3GAL1	0.135146701
2029	IPP	0.135121507
2030	HMGN1	0.135077187
2031	CCDC101	0.135046725
2032	NEDD8	0.135023571
2033	FLJ25006	0.135011286
2034	APEX2	0.135010411
2035	FBXO6	0.135000594
2036	RAB40C	0.134997847
2037	MAGEC2	0.134886161
2038	MC2R	0.134859765

2039	PRKAB1	0.134855015
2040	PELP1	0.134803887
2041	ARL8A	0.134784439
2042	INSM1	0.134746553
2043	C6orf64	0.134672574
2044	RFXANK	0.134614083
2045	RPS26	0.134532508
2046	STRBP	0.134510436
2047	MRPL51	0.134447411
2048	SEC23B	0.13444164
2049	MAGEA3	0.134418106
2050	DAG1	0.134417577
2051	KRT8	0.134365766
2052	FAM49B	0.13430051
2053	SENP3	0.13427007
2054	SPIN3	0.134245739
2055	SUV39H2	0.134242841
2056	KCNJ3	0.13420013
2057	RAD51C	0.134192821
2058	MAGEA4	0.134175055
2059	GREB1	0.134160299
2060	TUSC2	0.134157435
2061	NFKBIB	0.13414516
2062	TTC35	0.134145154
2063	CYB561D2	0.134066436
2064	ACTL6A	0.134038204
2065	DEFB126	0.133998441
2066	MANBAL	0.133953301
2067	C5orf22	0.133937991
2068	ZNF273	0.133929215
2069	KIF21A	0.133901104
2070	CCDC87	0.133852099
2071	PIH1D1	0.133838967
2072	SNCG	0.133819412
2073	ANKIB1	0.133698107
2074	AHCTF1	0.13368964
2075	RP11-529I10.4	0.133686555
2076	AKT2	0.13362446
2077	MRPL53	0.133613268
2078	KLRG2	0.133591939

2079	CYP2D7P1	0.133585321
2080	DCTN2	0.133482684
2081	NDUFA11	0.133434609
2082	KIAA1407	0.133419209
2083	UCRC	0.133403756
2084	ZNF703	0.133378667
2085	IFI6	0.133370857
2086	CHCHD5	0.133353935
2087	POLD2	0.133340748
2088	ZFP64	0.133328778
2089	CACNB3	0.133305595
2090	ANAPC1	0.133226366
2091	HNRNPA2B1	0.133201923
2092	FLJ33360	0.133141628
2093	LINGO1	0.133129388
2094	NARFL	0.133081787
2095	SNX4	0.133040922
2096	KIAA1751	0.133032877
2097	POLG2	0.133008859
2098	TMEM83	0.132993361
2099	FSHB	0.132974007
2100	KATNB1	0.1329713
2101	TMEM67	0.132968917
2102	ALDH18A1	0.132924582
2103	USP52	0.132814489
2104	ZC3HAV1	0.132761887
2105	HIST4H4	0.132747952
2106	C3orf63	0.132726567
2107	KIAA0100	0.13271732
2108	MGMT	0.132705863
2109	SLC23A1	0.132698859
2110	POU2F1	0.132636695
2111	VPS16	0.132590764
2112	PRIC285	0.132566756
2113	THEG	0.132534932
2114	MGC2752	0.132514588
2115	NUP93	0.132472682
2116	IMMT	0.132422741
2117	ATXN2L	0.132403709
2118	LOC441601	0.132329225

2119	TCOF1	0.132240907
2120	STMN3	0.13220582
2121	CTSA	0.132192963
2122	FAHD1	0.132185355
2123	AGPAT6	0.132176693
2124	G6PC2	0.132172212
2125	SUPT16H	0.132154872
2126	TSSC4	0.132146054
2127	ATG12	0.132079689
2128	FAM96B	0.132051254
2129	MGC16703	0.13204403
2130	SLC39A6	0.132038162
2131	SF3B2	0.131987676
2132	LOC554207	0.131966112
2133	DAND5	0.131951554
2134	CNTNAP2	0.131883354
2135	LRRC10	0.13188309
2136	EFHC1	0.131856592
2137	C19orf21	0.131844624
2138	C14orf145	0.13181363
2139	PLEKHA8	0.131810002
2140	SHROOM2	0.131793799
2141	AATK	0.131792415
2142	METTL5	0.13178704
2143	PRDX6	0.131696675
2144	ZNF28	0.131640828
2145	C11orf79	0.131608367
2146	MAPK8IP2	0.131510956
2147	MRPL11	0.131485635
2148	TBK1	0.13147425
2149	POP4	0.131472687
2150	TOMM34	0.131468239
2151	C16orf63	0.131458515
2152	RAD52	0.131422327
2153	GPATCH1	0.131414869
2154	SETD1B	0.131349475
2155	SAMD4B	0.131294754
2156	MC5R	0.131249804
2157	C9orf37	0.131236487
2158	KRT35	0.131231235

2159	MRPS33	0.131214466
2160	SYCP2	0.131207853
2161	SMARCA4	0.131184283
2162	TRUB2	0.131101765
2163	SLC4A8	0.131075397
2164	KCTD3	0.131036652
2165	PAH	0.131003261
2166	CUTA	0.130986216
2167	SLC10A7	0.130909865
2168	OR10A5	0.130883721
2169	TXNDC17	0.130876492
2170	EXDL2	0.130836224
2171	API5	0.130821035
2172	TMEM110	0.130749569
2173	CDKL3	0.130743843
2174	PRR13	0.130666593
2175	SRP14	0.130664879
2176	PEX5	0.130640125
2177	HPCAL1	0.130631279
2178	C10orf65	0.130623472
2179	OR6M1	0.130577732
2180	FANCD2	0.130540968
2181	PPFIA3	0.130517711
2182	HSP90B3P	0.130467071
2183	KRT24	0.130464753
2184	TMCC1	0.130459355
2185	QSOX2	0.130412835
2186	HMX2	0.130407067
2187	VWA3A	0.130295362
2188	TSR1	0.130223332
2189	LOC389517	0.130222872
2190	SEZ6	0.130209756
2191	MRPS27	0.130194874
2192	AGXT2L1	0.130176122
2193	LLGL1	0.130073028
2194	ZFP30	0.130044166
2195	TSFM	0.130038098
2196	ITFG3	0.130025751
2197	RPA1	0.13002015
2198	ATAD4	0.130002458

2199	FCRLB	0.13000217
2200	RALGPS1	0.129901018
2201	MGC3771	0.129785259
2202	OASL	0.129758667
2203	PFDN6	0.129682276
2204	RRS1	0.129670998
2205	CCDC112	0.12963022
2206	C3orf62	0.12959219
2207	EDG4	0.129526447
2208	LRRTM1	0.129515415
2209	HTR3E	0.129484829
2210	VAV2	0.129460328
2211	AMN1	0.129304119
2212	FH	0.12924686
2213	LOC285398	0.129211755
2214	CEP76	0.129159557
2215	PRDM15	0.129120022
2216	SQLE	0.129110604
2217	EVPL	0.129092118
2218	SCAMP1	0.129085854
2219	SNAP29	0.129081847
2220	E2F5	0.129037849
2221	ELAVL1	0.12901308
2222	CNOT10	0.128972369
2223	HSP90AB3P	0.128969484
2224	MAP7D2	0.128917824
2225	C11orf35	0.128911797
2226	TMPRSS6	0.128906719
2227	RPL23A	0.128884412
2228	DNAJB13	0.128875877
2229	EIF6	0.128874684
2230	PPFIA4	0.128853595
2231	R3HDM1	0.128839485
2232	KBTBD5	0.128808754
2233	MRPS24	0.128770186
2234	TMED10	0.128754581
2235	SFTPA1	0.128745867
2236	RBM12	0.128723549
2237	GMPR	0.128673813
2238	HSD17B7P2	0.12862564

2239	ITPR3	0.128616926
2240	PSME1	0.128615686
2241	C10orf82	0.128584252
2242	SELI	0.1285746
2243	MRPS10	0.128562611
2244	ZC3HC1	0.128524299
2245	COQ3	0.128505875
2246	UBE2L3	0.12847413
2247	INTS3	0.128470813
2248	PDIA4	0.128463262
2249	RNF34	0.128429483
2250	SPG11	0.128416527
2251	LOC137886	0.128395672
2252	KLF17	0.128331431
2253	FLJ12716	0.128300543
2254	GTF3C1	0.128300058
2255	14-Sep	0.128287648
2256	FADS2	0.128222737
2257	CACNG7	0.128201545
2258	TTC27	0.128156088
2259	HSBP1	0.128116677
2260	NDUFA6	0.127989664
2261	USMG5	0.127966446
2262	SCD	0.127942767
2263	B3GAT3	0.127931975
2264	CCDC45	0.127927255
2265	PRELID1	0.127904756
2266	CEMP1	0.127898392
2267	HSPC111	0.127886975
2268	HIST2H2AB	0.127842368
2269	CALB1	0.12781061
2270	MKL2	0.127758728
2271	WWOX	0.127673819
2272	TRAF2	0.127640283
2273	C19orf62	0.127636711
2274	MGC35440	0.127629977
2275	PLCG1	0.127579616
2276	COMMD5	0.127545282
2277	THEM4	0.127525441
2278	CNNM2	0.127406943

2279	NT5C	0.127371465
2280	DRG2	0.127280679
2281	SCAP	0.127253925
2282	SYAP1	0.127194199
2283	LOC149837	0.127154092
2284	TOR3A	0.127074641
2285	STAT3	0.12703948
2286	PUS7	0.127020759
2287	VTN	0.126967629
2288	ISL2	0.126946649
2289	TP53RK	0.126905805
2290	G6PC	0.126891363
2291	PTCH2	0.126757931
2292	COX6B1	0.126750732
2293	TMEM101	0.126749139
2294	TMEM16H	0.126744449
2295	PIR	0.126738782
2296	KIAA1219	0.126709332
2297	GML	0.126695463
2298	DNAI1	0.12667617
2299	GPR89A	0.126652288
2300	BRCA2	0.126634283
2301	PPP5C	0.126628908
2302	ALMS1L	0.126614867
2303	PPOX	0.12660367
2304	EEF1E1	0.126584105
2305	GPATCH4	0.12655111
2306	TRGV5	0.126514795
2307	RIMS4	0.126510766
2308	hCG_2001000	0.126509919
2309	OR5M8	0.126506299
2310	FASTK	0.126499715
2311	IMMP1L	0.126495081
2312	NFATC2IP	0.126397669
2313	ROS1	0.126380902
2314	DLG1	0.126304286
2315	KIF5B	0.126301332
2316	FLJ13195	0.12627002
2317	SMPD3	0.126246722
2318	STK35	0.126214416

2319	RTN4IP1	0.126157301
2320	CLTC	0.126123392
2321	C1orf66	0.126112764
2322	ATP6V1H	0.126076339
2323	GNAZ	0.126066562
2324	TMEM57	0.125971254
2325	RNPEP	0.125964923
2326	TCP10	0.125955385
2327	EDEM2	0.125885443
2328	SCRN2	0.125875955
2329	NEK4	0.125824163
2330	03-Sep	0.125821376
2331	UBE2NL	0.125809039
2332	LDHAL6B	0.125805986
2333	R3HDM2	0.125774924
2334	SGTA	0.125754527
2335	TBCCD1	0.125726493
2336	C10orf57	0.1257245
2337	VRK3	0.125675973
2338	OR10H1	0.125652271
2339	POLR3E	0.125627319
2340	SFXN2	0.125581378
2341	C1orf65	0.125543715
2342	RBM4B	0.125519641
2343	CLCN1	0.125515504
2344	TAF4	0.125475145
2345	RNASE8	0.125453712
2346	UNQ501	0.125437278
2347	UBN1	0.125404325
2348	MFSD5	0.125394603
2349	SPDYA	0.125375784
2350	ACOT4	0.125360844
2351	TMEM112	0.125360004
2352	ANKRD13D	0.125305975
2353	LOC134145	0.125286309
2354	LOC285636	0.125241362
2355	RNUXA	0.125240918
2356	CASP3	0.125235531
2357	CDK4	0.125186159
2358	ARID3A	0.125179993

2359	ALMS1	0.125143303
2360	FUT11	0.125120295
2361	PSMC3	0.125113394
2362	HTR1D	0.125075416
2363	C20orf121	0.125040714
2364	PRMT1	0.125019009
2365	WFDC13	0.125002057
2366	SIVA1	0.124997854
2367	RGS11	0.124994317
2368	TTTY10	0.124947577
2369	XYLB	0.124941248
2370	CRYBA2	0.124921999
2371	C6orf154	0.124798273
2372	UBE2W	0.124791645
2373	RRP15	0.124741215
2374	DHX37	0.12462626
2375	LOC93622	0.124568399
2376	SMC3	0.124547193
2377	ZADH1	0.124493059
2378	NARF	0.124406931
2379	MAGEA5	0.124403312
2380	ANGEL2	0.124344837
2381	MAGEA12	0.124343176
2382	NDUFS7	0.124317052
2383	CNGA4	0.124141386
2384	ZNF346	0.124127791
2385	TIMM44	0.124127664
2386	RBM4	0.124119277
2387	PSMD12	0.12407612
2388	RNASEH2C	0.124047303
2389	NIBP	0.123980571
2390	CCDC142	0.123969977
2391	C20orf11	0.123933369
2392	MSH6	0.123899178
2393	CANT1	0.123801787
2394	KIAA1712	0.123755486
2395	OR9K2	0.123617545
2396	SLC25A41	0.12361101
2397	TRPV1	0.123607327
2398	PRPS2	0.123508982

2399	C7orf27	0.123447718
2400	TBCB	0.123420913
2401	PSMB7	0.123387023
2402	ZC3H8	0.123382253
2403	LRRC59	0.123379963
2404	PPIL2	0.123334834
2405	TIMM10	0.123329144
2406	WDR57	0.123328759
2407	ELAC2	0.123258748
2408	CSTF2	0.123257088
2409	PIGQ	0.123216111
2410	ZNF607	0.123203745
2411	STRN4	0.123186717
2412	U1SNRNPBP	0.123150513
2413	MORF4L2	0.123139926
2414	UBE2D2	0.123126608
2415	ZMYND10	0.123061689
2416	GRB2	0.123048426
2417	OR7D4	0.123021326
2418	SEPHS2	0.122950528
2419	LYPLA1	0.122900867
2420	UMPS	0.122900642
2421	CACNA1B	0.122893419
2422	GPR135	0.122821004
2423	DNMT3L	0.122817425
2424	OR4D5	0.122776834
2425	LOC51252	0.122754478
2426	ALDH3A2	0.122655391
2427	CTDSPL2	0.122652583
2428	LCMT1	0.122639125
2429	ANAPC10	0.122614162
2430	TSKU	0.122589748
2431	RALY	0.122588932
2432	RHPN1	0.122582452
2433	ANKRD27	0.122551649
2434	TGS1	0.122545972
2435	WDR35	0.122506425
2436	TEX14	0.122489401
2437	MRPS14	0.122475372
2438	KLHL17	0.122453823

2439	LY6G6C	0.122431592
2440	AMHR2	0.122430242
2441	PSMC5	0.122353866
2442	STAM	0.122348259
2443	SLC27A3	0.122319385
2444	CANX	0.122305823
2445	LOC644186	0.12230187
2446	SIRT6	0.122123584
2447	METTL4	0.122115073
2448	hCG_1995786	0.122109505
2449	RP3-402G11.5	0.122057631
2450	C10orf99	0.122052538
2451	UCK2	0.122015023
2452	EEFSEC	0.121968568
2453	SERTAD3	0.121942399
2454	ATP5B	0.121919666
2455	LRRTM4	0.121912526
2456	ADAMTS13	0.121909461
2457	NDUFS2	0.121905435
2458	SHKBP1	0.121884566
2459	ZNF764	0.12185049
2460	ACCN2	0.121813807
2461	MORC2	0.121797913
2462	MYT1	0.121795966
2463	NFE2L1	0.121764404
2464	EPB41L1	0.121750578
2465	OR52E6	0.121722513
2466	C20orf27	0.121709728
2467	HTR7P	0.12168321
2468	RAB35	0.121641776
2469	LASP1	0.12162381
2470	UBE2I	0.121612863
2471	UCN	0.121591543
2472	ACD	0.121582295
2473	UBAC1	0.121555334
2474	SMS	0.121518202
2475	TMEM180	0.121450178
2476	LOC222699	0.121429387
2477	EMG1	0.121418576
2478	BYSL	0.121294637

2479	OTUD6A	0.12128732
2480	C10orf91	0.12126317
2481	RRP12	0.121261561
2482	CCRN4L	0.12125785
2483	COPS7B	0.121228352
2484	KPNA1	0.121139231
2485	PRR7	0.121069604
2486	SEC11C	0.12102599
2487	PSMD13	0.121021813
2488	HRH3	0.121007661
2489	STOML2	0.121004596
2490	SRMS	0.121004002
2491	STRAP	0.120967987
2492	C14orf143	0.120960661
2493	PIAS3	0.120912954
2494	YWHAG	0.1208609
2495	C16orf74	0.120825
2496	ZDHHC16	0.120810196
2497	NASP	0.120785747
2498	RABL2A	0.120761091
2499	RAP1GDS1	0.120758564
2500	PTDSS2	0.120726462
2501	UNC93B1	0.120723711
2502	NVL	0.120660843
2503	GSTO2	0.120564403
2504	SCAMP5	0.120544518
2505	MAP2K7	0.120524057
2506	TCEA1	0.120503934
2507	ACTG1	0.120447961
2508	RET	0.12039726
2509	NAGS	0.120365303
2510	SQSTM1	0.120302174
2511	INVS	0.120253243
2512	C3orf44	0.120226883
2513	TMEM106A	0.120224441
2514	NNT	0.120199561
2515	PAIP1	0.120194177
2516	TTC31	0.120174038
2517	DDX47	0.120164465
2518	SPRR2D	0.120161883

2519	C20orf185	0.120149601
2520	SRCRB4D	0.120099871
2521	SURF1	0.120075228
2522	STAMBP	0.120058657
2523	LAGE3	0.120028197
2524	RHOB	0.119968692
2525	SPSB2	0.119896914
2526	HAGHL	0.119859956
2527	HDAC10	0.119852426
2528	ZMAT5	0.119823491
2529	TFAP4	0.119811629
2530	ZNF174	0.119805693
2531	FAM27L	0.119765902
2532	RIBC1	0.119765032
2533	CEACAM6	0.119741278
2534	IRF5	0.119740722
2535	DUSP21	0.119684269
2536	MRO	0.119669908
2537	PLXNA3	0.119651698
2538	CGA	0.119613573
2539	MGC61571	0.119601929
2540	MRPS30	0.119540197
2541	C8orf30A	0.119530116
2542	PHTF2	0.119498596
2543	NP	0.119475852
2544	STX16	0.119472675
2545	KIAA1598	0.119411951
2546	GRN	0.119405712
2547	TCAP	0.119342262
2548	SSB	0.119338904
2549	UTP20	0.119211889
2550	MYCBPAP	0.119201302
2551	CREBL1	0.119177349
2552	GPX5	0.119171542
2553	FXYD4	0.119151273
2554	PLCD4	0.119063626
2555	SAC3D1	0.119055005
2556	NFYB	0.119043072
2557	BCMO1	0.119041961
2558	FAM134A	0.119020585

2559	RASGRP4	0.118990094
2560	POLD4	0.118957944
2561	FUK	0.118954339
2562	B4GALT7	0.118950853
2563	ATP6AP1	0.118939737
2564	ITPA	0.118883022
2565	EIF5B	0.118791039
2566	C1orf57	0.118779487
2567	SSH1	0.118730686
2568	C1orf215	0.11870456
2569	STIP1	0.118686297
2570	TRUB1	0.118603092
2571	MRPS17	0.118580045
2572	RB1CC1	0.118570482
2573	AYTL2	0.118561876
2574	HEXIM1	0.118554228
2575	NELL2	0.118498141
2576	ACYP1	0.118487677
2577	GSPT1	0.118453976
2578	DNPEP	0.118427852
2579	TXNRD2	0.118418872
2580	NEU1	0.118400379
2581	ZNF682	0.118388647
2582	RFX1	0.118349013
2583	TRIM11	0.118348473
2584	GPR25	0.118320629
2585	SLC25A5	0.118307476
2586	POLR1A	0.118292232
2587	MDH2	0.118253426
2588	LEO1	0.118253379
2589	TRAFD1	0.118228506
2590	PIB5PA	0.118146932
2591	C1orf211	0.118093728
2592	TESK2	0.11808881
2593	ISG15	0.118056206
2594	GLOD4	0.118006962
2595	LASS6	0.117979795
2596	NUDCD1	0.117889909
2597	POLR3H	0.117882914
2598	CHFR	0.117878508

2599	FAM24B	0.117840842
2600	ZNF337	0.117651948
2601	CHMP6	0.11765005
2602	LOC494150	0.117637071
2603	EIF3B	0.117624705
2604	KIAA1688	0.117575295
2605	CHUK	0.11748379
2606	SRRM2	0.11747997
2607	COQ6	0.117426958
2608	CXorf56	0.117354342
2609	MYLK2	0.11733665
2610	NUDT16L1	0.117328728
2611	OR4P4	0.117267316
2612	KIAA1841	0.117259679
2613	LELP1	0.117064902
2614	NIP30	0.117034389
2615	ANKRD24	0.117020315
2616	FKSG24	0.117009023
2617	SLC17A2	0.117003443
2618	STC1	0.116968018
2619	UBL4A	0.116963657
2620	SPTBN2	0.116944651
2621	CENPJ	0.116928218
2622	LRRC3	0.116900998
2623	AGBL5	0.116895925
2624	KCNK15	0.116859816
2625	ANKMY1	0.116851172
2626	OR8A1	0.116819152
2627	CEP135	0.116798931
2628	SPERT	0.116762026
2629	UEVLD	0.1167255
2630	TSR2	0.116720118
2631	ASB16	0.116707828
2632	ZMYM3	0.11660766
2633	EIF4E	0.116586742
2634	LOC57228	0.116575313
2635	PCSK1	0.116568503
2636	C8B	0.116557694
2637	FLJ38482	0.116555863
2638	C18orf2	0.116498761

2639	OR5P2	0.116412006
2640	SLC3A2	0.11637911
2641	KCNH2	0.116378691
2642	ERLIN1	0.11637644
2643	MCM3AP	0.116354664
2644	UGCGL1	0.116334785
2645	FAM80B	0.116314437
2646	EIF3J	0.116099384
2647	C1orf161	0.116058367
2648	LGALS3BP	0.116028152
2649	ADAM11	0.116019782
2650	RETNLB	0.115979164
2651	MYOZ3	0.115954003
2652	CACNG8	0.115943777
2653	HMG4L	0.115941235
2654	METAP1	0.115929398
2655	C17orf55	0.115891299
2656	FAM123C	0.115843292
2657	TMTC3	0.115800112
2658	C20orf77	0.115786997
2659	SCNN1D	0.115737972
2660	SLC16A6	0.115730085
2661	MC4R	0.115719969
2662	C10orf12	0.115716002
2663	FOXK2	0.115697655
2664	SCCPDH	0.115694024
2665	FLJ31033	0.115684038
2666	CSTF1	0.115682345
2667	OR8S1	0.115661513
2668	FOXD2	0.115618337
2669	BEX1	0.115593771
2670	ARFGEF2	0.115582638
2671	DHODH	0.115519848
2672	TMEM185A	0.115484922
2673	GGA2	0.11539596
2674	TUBB1	0.115375079
2675	HEATR4	0.115338785
2676	TMEM134	0.115335453
2677	OMG	0.115333496
2678	LOC643905	0.115317095

2679	SPIRE2	0.11531225
2680	RHBDD1	0.11530191
2681	ZNF169	0.115287749
2682	MAD1L1	0.115255151
2683	TGM3	0.115248108
2684	PUF60	0.115202645
2685	OR11G2	0.115180612
2686	VIL2	0.115175503
2687	C20orf117	0.11516801
2688	C8orf76	0.115155297
2689	ADAMTS15	0.115112738
2690	SSTR3	0.115089093
2691	CAMK1G	0.115084309
2692	CYorf15A	0.115079539
2693	ALS2CR2	0.115052903
2694	SHQ1	0.115047055
2695	RAB3A	0.115020853
2696	GK2	0.114913784
2697	SDCCAG10	0.114896605
2698	OR51S1	0.114865441
2699	TMEM184A	0.114845085
2700	UBE2V2	0.114830659
2701	KIAA0265	0.114817703
2702	MYEOV2	0.114817587
2703	ADRM1	0.114809009
2704	ETV2	0.114796738
2705	CHKA	0.114774845
2706	RTDR1	0.114747842
2707	DMC1	0.114733137
2708	FRS3	0.114707208
2709	SMYD4	0.114699326
2710	SH2B1	0.114654636
2711	DPM1	0.114623692
2712	PLA2G2E	0.114606454
2713	NOSIP	0.114582923
2714	DKFZp564N2472	0.114512528
2715	CCDC18	0.114496834
2716	OR8K1	0.114496337
2717	CCDC113	0.114480125
2718	10-Mar	0.114451484

2719	ADCY9	0.114421295
2720	SLC45A2	0.114418763
2721	GPHA2	0.11435388
2722	OLA1	0.114352491
2723	SF3B4	0.114341766
2724	MDK	0.114263065
2725	GPX4	0.114205374
2726	CCT6A	0.11415238
2727	LRRC14	0.114134147
2728	ABCF1	0.114125696
2729	POU4F2	0.114107119
2730	PRKCD	0.114086049
2731	EIF3A	0.114078231
2732	DISP2	0.11407207
2733	HAT1	0.114054704
2734	C1orf67	0.114027037
2735	KNG1	0.113997013
2736	PA2G4	0.113952155
2737	TSGA14	0.113913373
2738	FNDC8	0.113880619
2739	HADH	0.113871045
2740	MFSD9	0.113862839
2741	DLG5	0.11384447
2742	CHMP2A	0.113797722
2743	C22orf13	0.113643986
2744	LOC162632	0.113584615
2745	RND2	0.113578508
2746	ZCCHC10	0.113544632
2747	HD	0.113528872
2748	PRAMEF1	0.113475772
2749	HGS	0.113475086
2750	FANCM	0.11347281
2751	GPR172A	0.113440834
2752	CCDC114	0.113436501
2753	RNASEN	0.11334707
2754	LANCL1	0.113307895
2755	DUS2L	0.113298573
2756	ENPP7	0.113201905
2757	PIWIL3	0.113130031
2758	CPLX2	0.113074213

2759	TRAPPC4	0.113035873
2760	MLF2	0.113013746
2761	KIAA0182	0.112994627
2762	SASS6	0.112993154
2763	ATG4B	0.112919888
2764	GK	0.112887738
2765	KRT33B	0.112864407
2766	LOC201229	0.112856613
2767	DAGLA	0.11284395
2768	SOX2	0.112838908
2769	EHMT2	0.112756519
2770	ZFP41	0.112702196
2771	CHID1	0.112687333
2772	MACROD1	0.112644651
2773	ENTPD6	0.11261557
2774	NUCB2	0.112602183
2775	PDK3	0.112594335
2776	ALS2	0.112580242
2777	LCN8	0.112545096
2778	FXC1	0.112473589
2779	TCTN3	0.112458821
2780	AKAP10	0.112444555
2781	PPP2CA	0.112398805
2782	OR13A1	0.112372124
2783	UTY	0.112357968
2784	FKBP3	0.112325441
2785	VPS39	0.11231196
2786	FTSJ1	0.112271842
2787	DUS1L	0.112243484
2788	NUFIP2	0.112222769
2789	CCDC41	0.112190105
2790	CYP2F1	0.112164067
2791	TJAP1	0.112144285
2792	SSH3	0.112101346
2793	SLC37A4	0.112095213
2794	PARP12	0.112061889
2795	CYP46A1	0.112054814
2796	FXN	0.112021699
2797	KIAA0143	0.112009682
2798	PLEKHH1	0.112004219

2799	OR10P1	0.111987203
2800	C20orf149	0.111979328
2801	OCIAD2	0.111943372
2802	ACRV1	0.111943308
2803	OR52N4	0.111876868
2804	FANCE	0.111869253
2805	MGC16169	0.111864899
2806	GLTP	0.111855096
2807	TAS2R5	0.111829558
2808	AMZ1	0.111799409
2809	NPC1L1	0.111754381
2810	PLA2G10	0.111748228
2811	NOL5A	0.11174185
2812	FAM29A	0.111734697
2813	DDX56	0.111700427
2814	C6orf130	0.111678658
2815	HAP1	0.111673934
2816	NPY6R	0.111644535
2817	SKP2	0.111637072
2818	UBC	0.1115966
2819	LMBR1	0.111551347
2820	COL20A1	0.111542372
2821	MSMB	0.111541932
2822	WDR42A	0.111541594
2823	MAGED2	0.111534307
2824	AP1M2	0.111501143
2825	LIM2	0.111481637
2826	POU5F2	0.111455965
2827	C6orf153	0.111430363
2828	CTPS2	0.111376825
2829	RCCD1	0.11135991
2830	KRTAP19-1	0.111341167
2831	VCP	0.111288438
2832	SRA1	0.111275764
2833	DPH4	0.111240639
2834	CAMKV	0.11118722
2835	OTX2	0.11117956
2836	RPL23	0.111177806
2837	COQ10A	0.111115857
2838	TJP3	0.111094153

2839	KIAA0317	0.111085834
2840	CDK2AP2	0.111064394
2841	QPRT	0.111050565
2842	OTOS	0.111028882
2843	P2RXL1	0.111010108
2844	STARD3	0.110988046
2845	C17orf28	0.110948398
2846	DUSP12	0.110916109
2847	DEPDC5	0.110890122
2848	DHX57	0.110888983
2849	FBXL6	0.110880035
2850	CROP	0.110866455
2851	BCL2L12	0.11086438
2852	IFT140	0.110816601
2853	PLA2G12A	0.110791264
2854	IMPG2	0.110750647
2855	C7orf34	0.110748086
2856	AUP1	0.110720381
2857	SLC22A5	0.11069089
2858	ABCB8	0.110658304
2859	RABGGTB	0.11065347
2860	CCL16	0.110647759
2861	MBD3L1	0.110646821
2862	FAM9B	0.110634022
2863	CCDC40	0.110623179
2864	IFIT1	0.110608758
2865	ANKRD54	0.110591055
2866	C16orf35	0.110562812
2867	ALKBH2	0.110493706
2868	OR5D16	0.110483757
2869	MBD4	0.110476933
2870	CLCN7	0.110469507
2871	SLC30A10	0.110442232
2872	FAM136A	0.110419794
2873	MLANA	0.110419411
2874	HNRPK	0.110407986
2875	MAP3K7IP1	0.11036211
2876	FERD3L	0.110359837
2877	C11orf47	0.110287138
2878	SNX24	0.110278748

2879	PFN3	0.110253218
2880	PRMT7	0.110251826
2881	ZDHHC22	0.110250636
2882	HDPY-30	0.110249326
2883	MCOLN1	0.110203789
2884	KIFAP3	0.110195173
2885	SMARCD2	0.110192107
2886	OR5BF1	0.110106853
2887	ASH2L	0.110082107
2888	C8orf41	0.110024875
2889	C3orf34	0.110009726
2890	STARD7	0.110007782
2891	HUS1B	0.109987053
2892	GNPAT	0.109982312
2893	NOX5	0.109969079
2894	USP40	0.10996503
2895	SLC35B2	0.109964465
2896	ELMO3	0.109956193
2897	VDAC2	0.109943624
2898	CCDC16	0.109905853
2899	SAG	0.109899052
2900	IRAK1	0.109875713
2901	PEX12	0.109868531
2902	MRPL23	0.109857931
2903	NS3BP	0.109813583
2904	KIAA0133	0.109804272
2905	CYP2C18	0.109793036
2906	C1orf131	0.109765261
2907	MYADML	0.109747374
2908	NPAS1	0.109742711
2909	TRIM36	0.109737556
2910	XYLT2	0.109731383
2911	DECR2	0.109663062
2912	OR7A17	0.109656989
2913	C10orf122	0.109630365
2914	C3orf39	0.10962466
2915	HSPA1A	0.109590102
2916	LOC442582	0.109568193
2917	CCDC63	0.109511494
2918	SIP1	0.109508082

2919	NBEAL2	0.109507843
2920	JMJD4	0.109504369
2921	RAPSN	0.109501976
2922	TMEM49	0.109485738
2923	SNRPE	0.109483617
2924	OR52D1	0.1094739
2925	XRCC1	0.109419612
2926	CAPS	0.109419072
2927	MTG1	0.109406489
2928	C20orf7	0.109378532
2929	PPP1R7	0.109335479
2930	C14orf100	0.109327107
2931	CCDC52	0.109326061
2932	FOXRED1	0.109299806
2933	SENP5	0.10926451
2934	ZDHHC12	0.109259389
2935	BRD9	0.10925917
2936	ERGIC1	0.109252253
2937	C21orf87	0.109227565
2938	TCERG1	0.109220499
2939	ADCYAP1R1	0.109181569
2940	AARS	0.109166751
2941	MED12L	0.109129815
2942	PRDM8	0.109124273
2943	NDUFB2	0.109118521
2944	KIF3A	0.109080222
2945	PREB	0.109078089
2946	C7orf24	0.109060074
2947	TOP3A	0.109036955
2948	SPTBN4	0.109020251
2949	BCL7A	0.108982745
2950	ABHD1	0.108970214
2951	MAST2	0.108966048
2952	RERG	0.108841704
2953	METTL6	0.10881507
2954	SYCN	0.108798942
2955	ERCC4	0.108744101
2956	ABCA3	0.10871005
2957	SERF2	0.108652511
2958	ROGDI	0.108610293

2959	MAT2A	0.108596832
2960	GLDC	0.10859175
2961	COX7B	0.108572752
2962	OR1S1	0.108557872
2963	SKIV2L	0.108519172
2964	NOS1	0.108514819
2965	CARS	0.108493574
2966	CHAC1	0.108420873
2967	GRID2	0.10840133
2968	CEBPG	0.108334911
2969	PAPD5	0.108325468
2970	RNF14	0.108299348
2971	IFNA16	0.108298543
2972	RFXDC2	0.108259723
2973	HIST1H2AA	0.108230021
2974	ARFGAP1	0.108165257
2975	EXOC6	0.108162149
2976	AIFM2	0.108138516
2977	GTF2H4	0.108102641
2978	LASS5	0.108092905
2979	YWHAQ	0.108076967
2980	MED20	0.108064939
2981	TRMT12	0.108058548
2982	ALDOAP2	0.10803497
2983	CREB3L4	0.107998363
2984	OR4K14	0.107992825
2985	OR6C4	0.107948754
2986	TTMA	0.107923738
2987	RBKS	0.107900246
2988	FLJ13611	0.107896179
2989	UNC93A	0.107890576
2990	SCML2	0.107872949
2991	FRMD1	0.107854698
2992	HNRNPC	0.107833739
2993	PPM1E	0.107824037
2994	ZMYND8	0.107744333
2995	LRP8	0.107716007
2996	C20orf30	0.107681369
2997	TSPAN13	0.107647253
2998	HIST1H2AL	0.107632328

2999	SOHLH1	0.107610536
3000	NIPSNAP1	0.107583087
3001	TMEM141	0.107577627
3002	CCDC111	0.107552008
3003	NUP107	0.107524888
3004	FAM82C	0.107450148
3005	UBE2Z	0.107406409
3006	SDHC	0.107395801
3007	NUP188	0.107384319
3008	UNC13A	0.107364693
3009	AIM1L	0.107351201
3010	ZNF553	0.107348327
3011	RPGR	0.107337631
3012	ARMET	0.10731425
3013	MAS1L	0.107297813
3014	LOC728378	0.107270858
3015	RFX5	0.107266634
3016	ADCK5	0.107261559
3017	ZCCHC8	0.107254303
3018	TAF9	0.107247992
3019	FLJ46111	0.107245056
3020	NDUFC1	0.107219784
3021	SIRT7	0.107179599
3022	FLJ22222	0.107178481
3023	ZBTB2	0.107121134
3024	AIP	0.107103105
3025	NHEDC1	0.107100233
3026	ALOX12	0.107078868
3027	PMPCA	0.107054241
3028	OR6A2	0.107052733
3029	HIST1H3C	0.106995105
3030	SPCS1	0.10699079
3031	PRMT8	0.106942644
3032	C12orf51	0.106896219
3033	ABCE1	0.106884252
3034	PNN	0.106866359
3035	MDM1	0.106851329
3036	CDS2	0.106840328
3037	C20orf177	0.106839363
3038	NDUFB6	0.10680726

3039	SAR1B	0.106731182
3040	HEMK1	0.106715211
3041	SPINK4	0.106702561
3042	MED31	0.106700431
3043	CSMD3	0.106667589
3044	SLC25A28	0.106656306
3045	FANCL	0.106587928
3046	SPAR	0.106553327
3047	CST9	0.106520892
3048	GNRHR	0.106488826
3049	UNQ1940	0.106487514
3050	C1orf210	0.106475646
3051	CPSF4	0.106407133
3052	TMEM179	0.106401983
3053	C6orf141	0.106371356
3054	PTCHD2	0.106358586
3055	ACP2	0.106311766
3056	PERLD1	0.106272468
3057	PPP1R16A	0.106267691
3058	WNT3A	0.106261255
3059	UBXD2	0.106236611
3060	CRX	0.106204551
3061	C20orf135	0.106203454
3062	MGC52110	0.106156921
3063	SYNJ1	0.106120998
3064	PSMB4	0.106107193
3065	RAMP1	0.106101913
3066	C1orf96	0.106030275
3067	GRSF1	0.105957511
3068	IQWD1	0.105950777
3069	RBM12B	0.105941969
3070	CNNM4	0.105941718
3071	FAM120A	0.10592336
3072	MRPL4	0.105898358
3073	TSG101	0.105855221
3074	PAK4	0.105854136
3075	PSPN	0.105851327
3076	HSPA4	0.10584157
3077	FLJ38596	0.105840928
3078	C9orf116	0.105836609

3079	NOL11	0.105828415
3080	MAPK9	0.105825646
3081	FARS2	0.10581977
3082	IPO7	0.105809875
3083	NDUFB7	0.10578549
3084	OR1L3	0.105741203
3085	ERBB2	0.105708907
3086	LOC790955	0.105683621
3087	CENPB	0.105671946
3088	C5	0.105620531
3089	MARS	0.105614022
3090	PDSS1	0.105613274
3091	TRIB3	0.105600401
3092	PEX11G	0.105543048
3093	PAQR7	0.105502927
3094	GRHPR	0.105484375
3095	TRIM24	0.105464989
3096	SERF1A	0.105459954
3097	SMARCC1	0.10543596
3098	C10orf58	0.105418838
3099	TBRG4	0.105414762
3100	PCTK2	0.105409385
3101	C19orf51	0.105393072
3102	GFER	0.105377168
3103	ZNF552	0.105354946
3104	KCNH7	0.105290277
3105	ZSCAN16	0.105266014
3106	NXT1	0.10525245
3107	ILKAP	0.105249477
3108	OR51B6	0.105246665
3109	TM7SF3	0.105237344
3110	HOXC4	0.105211557
3111	PSMD6	0.105137906
3112	PAGE2	0.105126842
3113	CTF1	0.105124057
3114	ELK1	0.105104657
3115	DLL3	0.105064088
3116	ENY2	0.105035763
3117	ATAD2B	0.105026835
3118	GPATCH8	0.105002336

3119	KIAA1279	0.10500021
3120	RAB5B	0.104994547
3121	RAB8A	0.104992279
3122	MYOG	0.104979932
3123	ZNF263	0.104939126
3124	GFI1B	0.1049326
3125	PDIA2	0.104892984
3126	POLR2E	0.10487335
3127	KIAA0152	0.104850908
3128	TERF1	0.104824623
3129	CSAG3A	0.104802877
3130	HNRNPU	0.104791667
3131	SNX21	0.104789703
3132	ESR1	0.104778509
3133	DKFZp761E198	0.104768325
3134	AFF3	0.10474686
3135	C18orf8	0.104724116
3136	HEATR6	0.104722495
3137	RSL1D1	0.104722256
3138	GUCA2A	0.104708102
3139	MAG	0.104704751
3140	ACOT6	0.104695465
3141	C9orf7	0.104682875
3142	INTS8	0.104674668
3143	HYAL4	0.104664923
3144	PISD	0.104649525
3145	TMEM28	0.104633669
3146	TMEM41A	0.104602162
3147	C9orf31	0.104572374
3148	TMEM11	0.104532765
3149	ISCA2	0.104484524
3150	PARL	0.104466004
3151	LSM2	0.10444902
3152	CDCA4	0.104427905
3153	CES3	0.104369926
3154	OR51B5	0.104369621
3155	SLC7A9	0.104303405
3156	PGLYRP3	0.104286576
3157	NBN	0.104266789
3158	MS4A8B	0.104230056

3159	C2orf37	0.104206241
3160	DKC1	0.104156497
3161	TMEM137	0.104147011
3162	NOL1	0.104118441
3163	ARD1B	0.10410214
3164	CTPS	0.104063244
3165	SYNGR3	0.104062648
3166	CLN6	0.104048364
3167	CELP	0.104018314
3168	CTAGE1	0.103911584
3169	POLR3B	0.10389494
3170	CSAG1	0.10386435
3171	RAD1	0.103863093
3172	APPBP2	0.103827078
3173	DDX3Y	0.103812565
3174	SLC47A1	0.103784096
3175	SLC25A13	0.103779204
3176	MED11	0.103755583
3177	ATOX1	0.10372653
3178	OPRS1	0.103717472
3179	SAAL1	0.103715692
3180	PUS7L	0.103708716
3181	CYB5D1	0.103693422
3182	THOC1	0.103690366
3183	OR6W1P	0.1036753
3184	VKORC1L1	0.103661371
3185	NXF5	0.103616128
3186	RFWD2	0.103598199
3187	CTR9	0.103592128
3188	IVD	0.103569103
3189	NAT9	0.103556666
3190	NSMCE4A	0.103542071
3191	CAMKK2	0.103534574
3192	SNRP70	0.103523562
3193	OR10W1	0.10351898
3194	HSD17B8	0.103503705
3195	CALM2	0.103498266
3196	MLH3	0.103487254
3197	POLR2I	0.103440052
3198	TCEA3	0.103429364

3199	ALDOA	0.103403613
3200	AFM	0.103395983
3201	CCDC104	0.10338947
3202	ATP5J2	0.103370559
3203	KRTAP4-10	0.103362713
3204	CCDC74B	0.103347545
3205	C14orf169	0.103292913
3206	CEACAM3	0.103290701
3207	AARS2	0.103263733
3208	SCRIB	0.103255054
3209	TMED2	0.103251469
3210	ZCCHC4	0.10324045
3211	ATE1	0.103190116
3212	ZSCAN29	0.103184818
3213	KRTAP9-3	0.103171811
3214	ATP6V0C	0.103166104
3215	THEM2	0.103144482
3216	CRLS1	0.103068104
3217	D2HGDH	0.103043961
3218	FAM119A	0.102983534
3219	EXOSC3	0.102953097
3220	TH	0.102940832
3221	TMEM41B	0.102940749
3222	CRIP3	0.102933355
3223	PRR6	0.102927373
3224	PHB	0.102898551
3225	DHRS7B	0.102893836
3226	ASNA1	0.102845379
3227	RNF139	0.10283142
3228	CLIP1	0.102831029
3229	C1orf77	0.102791961
3230	SPACA5	0.102779613
3231	AP2A2	0.102775931
3232	LSM1	0.102764897
3233	OR4C46	0.102729764
3234	KLHDC5	0.102726466
3235	SLC5A12	0.102718742
3236	CASP6	0.102717094
3237	ELOVL3	0.102715647
3238	PIGN	0.102609205

3239	MTERFD3	0.102588134
3240	USF2	0.102585621
3241	EXOSC1	0.102578335
3242	UBE2M	0.102571031
3243	VANGL1	0.102498057
3244	WDR4	0.102496882
3245	KRT8P12	0.102475561
3246	C8ORFK29	0.102438352
3247	SIAH2	0.102436284
3248	ETFDH	0.102433093
3249	SPIRE1	0.102384055
3250	C17orf73	0.102383679
3251	RIMS1	0.102340728
3252	SEC23IP	0.102313131
3253	GLS2	0.102287841
3254	INHA	0.102254707
3255	NDUFB4	0.102237703
3256	MGC5590	0.102201035
3257	APBB3	0.102186621
3258	ALB	0.102174209
3259	WFDC9	0.102161817
3260	ZNF217	0.102127054
3261	C3orf15	0.102126207
3262	APC2	0.102095696
3263	TAC4	0.102076572
3264	MORC1	0.102061567
3265	CCDC15	0.102056588
3266	RABL5	0.101997105
3267	OR4B1	0.101969409
3268	OR6N1	0.101878288
3269	KRR1	0.101871669
3270	SNAP25	0.101852453
3271	VPRBP	0.101817382
3272	DHCR7	0.101815602
3273	OR5M10	0.1018135
3274	TBC1D3P2	0.101809488
3275	NOXO1	0.101794529
3276	C19orf61	0.101649079
3277	PSMB1	0.101643813
3278	CTAG2	0.101591286

3279	LDLRAD1	0.101580567
3280	PTPRA	0.101579661
3281	RHOT2	0.101560287
3282	SPHK2	0.101559781
3283	SMPD2	0.101550412
3284	MYST2	0.101542516
3285	MCHR1	0.101537929
3286	HSPA14	0.10148706
3287	LIMD1	0.101482827
3288	TNFAIP2	0.101468352
3289	BAIAP2L1	0.101447491
3290	PTPMT1	0.101421902
3291	DSCR3	0.101419718
3292	LSM14A	0.101385372
3293	SLC5A11	0.101357061
3294	KIR2DL3	0.101313367
3295	RQCD1	0.101280946
3296	SFPQ	0.101271249
3297	GNE	0.10125624
3298	CDCP2	0.101239782
3299	H3F3B	0.101231595
3300	HDAC5	0.101222132
3301	ARID2	0.101202516
3302	FLJ90709	0.101182061
3303	RAB24	0.101180204
3304	LCMT2	0.101169219
3305	OR52L1	0.101165461
3306	SLC2A4RG	0.101155527
3307	NCBP2	0.101134922
3308	VAMP8	0.101102912
3309	ANKRD22	0.101101064
3310	CARD14	0.101066651
3311	OR11H4	0.100988676
3312	C4orf17	0.10093504
3313	PSIP1	0.100847063
3314	LPIN3	0.100819857
3315	IKZF4	0.100818909
3316	HIST1H3I	0.100816766
3317	ZDHHC23	0.100803255
3318	DPY19L2P1	0.100786499

3319	RNF25	0.10071422
3320	SERPINA5	0.100700656
3321	WDR31	0.100682893
3322	OR5H1	0.100682316
3323	KIR3DL3	0.100661548
3324	C1orf74	0.10064517
3325	ONECUT1	0.100617658
3326	SCARB1	0.100569994
3327	PPIAL4	0.100568153
3328	RHOXF2B	0.100555777
3329	SLC13A4	0.100552166
3330	C11orf31	0.10054181
3331	AGRP	0.100535611
3332	GCAT	0.100528316
3333	ATPIF1	0.100524832
3334	PRCC	0.10051226
3335	KPNA6	0.100483999
3336	PVRL2	0.100467565
3337	SEC61A1	0.100455461
3338	PGLYRP2	0.100449725
3339	PLP2	0.100349988
3340	AGPAT1	0.100339661
3341	P2RX4	0.100322988
3342	FLCN	0.100320651
3343	C10orf4	0.100308647
3344	PCCB	0.100291312
3345	ZNF598	0.100274035
3346	C17orf62	0.100239912
3347	MAD2L2	0.10021431
3348	C15orf24	0.100202641
3349	IL22RA1	0.100142694
3350	FLJ20035	0.100102246
3351	PRDM4	0.100101499
3352	JMJD2B	0.100095894
3353	PDXDC1	0.100040679
3354	GALNT6	0.10003618

Supplementary Table 3. Putative BRCA1 target gene candidates

Gene Name	ENCODE data (CHIP seq)	BRCA1-KD/control_MCF7	BRCA1_overExp/Con_HCC1937	TCGA Correlation
ADSS	(-72)	0.597	2.182	-0.042
AHSA2	(-352)	0.327	0.418	0.042
CCDC47	(+49)	0.540	3.502	0.068
CENPE	(-76)	0.463	0.155	0.367
CENPN	(+805)	0.664	2.454	0.173
**CKS1B**	**(-319)**	**0.661**	**1.906**	**0.289**
COASY	(-12)	0.865	0.529	0.371
DARS2	(-11)	0.905	0.289	0.333
DTYMK	(-341)	0.883	3.792	0.341
EFCAB2	(+1190)	0.496	2.188	0.073
EIF3K	(-18)	0.689	2.458	-0.004
EIF5B	(-959)	0.547	0.442	0.119
ENSA	(+260)	0.467	2.181	0.098
**FADD**	**(+152)**	**1.060**	**2.096**	**0.244**
FYTTD1	(-8)	0.566	0.411	0.026
GRWD1	(-158)	0.617	2.249	0.155
HMGB2	(-1054)	1.137	0.368	0.352
HMGCS1	(-283)	1.677	0.338	0.093
IFNGR1	(+434)	1.159	3.133	-0.299
IRF9	(+735)	1.700	2.601	0.058
KIF15	(+76)	0.678	0.356	0.335
KNTC1	(-477)	0.777	0.528	0.382
**MEIS2**	**(+521171)**	**0.772**	**1.010**	**-0.318**
MRPL42	(+251)	0.503	3.203	0.136
NUCKS1	(+1046)	0.681	2.474	0.019
NUF2	(-383)	0.773	0.523	0.314
PCM1	(-54)	0.693	0.428	-0.012
PDE7A	(-605)	0.607	2.574	-0.013
PFKFB3	(+52)	1.923	2.343	-0.056
PIH1D1	(-1635), (+47)	0.631	2.438	0.134
PIK3R3	(-191)	0.522	4.262	-0.064
PNKP	(-2225)	0.575	2.226	0.089
RAP1GDS1	(-414)	0.400	1.521	0.121
RELB	(-158)	1.675	5.452	-0.143
RPS2	(-69)	0.538	2.141	0.088
SH3BGRL	(+133)	0.667	2.037	-0.022
STK38L	(-8)	2.833	1.625	-0.092
TAP2	(-112)	1.571	2.031	0.033
TBC1D5	(-102)	0.630	0.466	-0.131
TMPO	(+13)	0.608	1.202	0.350
TRIM26	(+360)	1.821	2.021	0.006
TUBA1B	(+1070)	1.035	0.456	0.373

**UBE2S**	**(-94)**	**0.651**	**2.885**	**0.255**
ZNF33B	(-125)	0.505	0.494	-0.114
ZNF697	(-867)	2.410	1.907	-0.049

Supplementary Table 4. Sequences for ChIP Primers

Gene name (length of DNA sequence)	Primer sequence
**CKS1B (292)**	F: 5’-GGGGGG AGAAAAACTGGGCGACAGGG
	R: 5’-GGGGGG TGGAGCGGTAACCTAAGCTG
**FADD (280)**	F: 5’-GGGGGG CTTTGCAAACAGGTGGACTCG
	R: 5’-GGGGGG CTCCGGTGCCTGATTCACTA
**MEIS2 (268)**	F: 5’-GGGGGG AGACATGGTTTCTCGGCAGG
	R: 5’-GGGGGG CGCCAGAGTTTCAGTAGGCA

Supplementary Table 5. Sequences for promoter PCR Primers

Gene name	Restriction Enzyme	Primer sequence
**CKS1B**	SacI	F: 5’-CCC**GAGCTC**GAAGTGAGGCTGGGAGTCTG
	KpnI	R: 5’-CCC**GGTACC**GAGGCGGGACACCTCTACTTTC
**FADD**	XhoI	F: 5’-CCC**CTCGAG**TTGAATGGTATGTGAATTATATC
	HindIII	R: 5’-CCC**AAGCTT**ACAGGGCTACTGCGAAAATTG
**MEIS2**	NheI	F: 5’-CCC**GCTAG**CAATGTCGACGAGCAACTCAGC
	XhoI	R: 5’-CCC**CTCGAG**AGGAAGTGAGGAGGCAGTCATC

Supplementary Table 6. Sequences for real time PCR Primers

Gene name	Primer sequence
**CKS1B**	F: 5’-GGGGGG TATTCGGACAAATACGACGACG
	R: 5’-GGGGGG CGCCAAGATTCCTCCATTCAGA
**FADD**	F: 5’-GGGGGG ATGGACCCGTTCCTGGTGC
	R: 5’-GGGGGG TCAGGACGCTTCGGAGGTAG
**MEIS2**	F: 5’-GGGGGG GAGACCACGATGATGCAACCT
	R: 5’-GGGGGG ACTGTTGTCTAAACCATCCCCTT
